# A Checklist of the Olethreutini Obraztsov, 1946 (Lepidoptera: Tortricidae) of Taiwan, with the Distribution in Mainland China

**DOI:** 10.3390/insects15080630

**Published:** 2024-08-22

**Authors:** Yinghui Sun, Houhun Li

**Affiliations:** 1 College of Life Sciences, Dezhou University, Dezhou 253023, China; sunyh8789@dzu.edu.cn; 2 College of Life and Geographic Sciences, Kashi University, Kashi 844000, China; 3 Xinjiang Key Laboratory of Biological Resources and Ecology of Pamirs Plateau, Kashi 844000, China; 4 College of Life Sciences, Nankai University, Tianjin 300071, China

**Keywords:** checklist, China, description, Taiwan Province, taxonomy, tortricid fauna

## Abstract

**Simple Summary:**

This study examined specimens of the tribe Olethreutini collected from more than 60 sites in Taiwan between the years 1921 and 2005, listing 102 species in 35 genera. The specimens are deposited in the National Museum of Natural History, Washington, DC, USA (USNM), and the McGuire Center for Lepidoptera and Biodiversity, Florida Museum of Natural History, University of Florida, Gainesville, Florida, USA (FSCA). At the same time, the distribution of these species in mainland China was examined, and the research specimens are deposited in the Insect Collection, Nankai University, Tianjin, China (NKU). This study makes up for the shortcomings of the previous research on the Chinese tortricids, which is conducive to the comprehensive completion of the systematic research of Chinese fauna and also provides basic data for the systematic research of the world’s leaf-roller moth.

**Abstract:**

Thirty-five genera and one hundred and two species of the tortricid tribe Olethreutini documented from Taiwan are listed, two genera and six species of which are newly recorded for China and two genera and eighteen species of which are newly recorded for Taiwan. Nine species are recorded in the mainland of China for the first time, which were endemic to Taiwan before this study. The local monographs of China and references are summarized with the examined specimens. The synonymies and geographic distribution in China are provided for each species, and a list of examined specimens is provided when applicable.

## 1. Introduction

The tribe Olethreutini belongs to the subfamily Olethreutinae in the family Tortricidae. Olethreutini was erected by Obraztsov in 1946 [1]. Currently, 58 genera and 541 species are described worldwide [2], a few of which are important pests of crops, fruit, and forest trees and of economic significance in agroforestry. Prior to this study, 78 species in 31 genera were recorded from Taiwan [3,4]. In recent years, several local monographs were published one after another, like Mt. Tianmu, Fujian, Hebei, and Mt. Qingling which recorded a lot of collection location information [5,6,7,8]. 

The purpose of this contribution is to provide a list of one hundred and two species of the tribe Olethreutini recorded from Taiwan, two genera and six species of which are newly recorded from China and two genera and eighteen species of which are newly recorded from Taiwan. It is found that nine Taiwan endemic species are distributed in the mainland of China in this study. The synonymies and geographic distributions are provided for each species, and a list of examined specimens is provided when applicable. The specimens of *Olethreutes meifengensis* Kawabe, 1993, *Sorolopha liochlora* (Meyrick, 1914), and *Sorolopha elaeodes* (Lower, 1908) collected from Taiwan were not examined in this study. Further research will be conducted in the future.

## 2. Materials

The studied specimens of Olethreutini from Taiwan are deposited in the National Museum of Natural History, Washington, DC, USA (USNM), the McGuire Center for Lepidoptera and Biodiversity, Florida Museum of Natural History, University of Florida, Gainesville, FL, USA (FSCA), and the Insect Collection, Nankai University, Tianjin, China (NKU). The specimens of Olethreutini from the mainland of China are deposited in the Insect Collection, Nankai University, Tianjin, China (NKU). The Institutional abbreviations see Table 1.

## 3. Results

The 102 species in 35 genera of Olethreutini recorded from Taiwan are listed below along with the collection records, including the following:

Two genera newly recorded for China: *Cimeliomorpha* Diakonoff, 1966, and *Megalota* Diakonoff, 1966; six species newly recorded for China: *Dicephalarcha herbosa* (Meyrick, 1909), *Dudua crossotoma* (Meyrick, 1931), *Megalota fallax* (Meyrick, 1909), *Neopotamia ioxantha* (Meyrick, 1907), *Olethreutes perexiguana* Kuznetzov, 1988, and *Cimeliomorpha cymbalora* (Meyrick, 1907). 

Two genera newly recorded for Taiwan: *Pseudohermenias* Obraztsov, 1960, and *Sycacantha* Diakonoff, 1959. 

Eighteen species newly recorded for Taiwan: *Archilobesia drymoptilia* (Meyrick, 1933), *Dudua dissectiformis* Yu et Li, 2005, *Eudemis porphyrana* (Hübner, [1796–1799]), *Hedya auricristana* (Walsingham, 1900), *Lobesia reliquana* (Hübner, [1825] 1816), *Olethreutes doubledayana* (Barrett, 1872), *Phaecasiophora diluta* Diakonoff, 1973, *P. leechi* Diakonoff, 1973, *P. obraztsovi* Diakonoff, 1973, *P. pyragra* Diakonoff, 1973, *P. walsinghami* Diakonoff, 1959, *Archilobesia drymoptilia* (Meyrick, 1933), *Pseudohermenias hercyniana* (Bechstein et Scharfenberg, 1804), *Rudisociaria velutinum* (Walsingham, 1900), *Sorolopha agana* (Falkovitsh, 1966), *S. camarotis* (Meyrick, 1936), *S. phyllochlora* (Meyrick, 1905), and *Stathrotmantis shicotana* (Kuznetzov, 1969).

Nine species are recorded on the mainland of China for the first time, which were endemic to Taiwan before this study: *Dactylioglypha tonica* (Meyrick, 1909), *Dicephalarcha herbosa* (Meyrick, 1909), *Hedya anaplecta* (Meyrick, 1909), *Neopotamia punctata* Kawabe, 1989, *Olethreutes meifengensis* Kawabe, 1993, *Proschistis marmaropa* (Meyrick, 1907), *Sorolopha elaeodes* (Lower, 1908), *S. herbifera* (Meyrick, 1909), and *S. liochlora* (Meyrick, 1914).

### 3.1. Apotomis *Hübner, [1825] 1816*

*Apotomis* Hübner, [1825] 1816: 380. Type-species: *Apotomis turbidana* Hübner, [1825] 1816.

*Aphania* Hübner, [1825] 1816: 386. Type-species: *Tortrix scriptana* Hübner, [1796–1799] [=*Tortrix lineana* Denis et Schiffermüller, 1775].

*Limma* Hübner, [1825] 1816: 381. Type-species: *Tortrix inundana* Denis et Schiffermüller, 1775.

*Antithesia* Stephens, 1829: 46. Type-species: *Tortrix corticana* Denis et Schiffermüller *sensu* Hübner [1796–1799] [=*Apotomis turbidana* Hübner, [1825] 1816].

*Apotomus* Agassiz, 1848: 87 [unjustified emendation of *Apotomis*].

*Brachytaenia* Stephens, 1852: 25. Type-species: *Tortrix semifasciana* Haworth, 1811 [1812]. 

#### 3.1.1. *Apotomis davisi* Kawabe, 1993

*Apotomis davisi* Kawabe, 1993: 228. TL: Taiwan, Nantou Hsien, Mei-feng, 30 km S Tayuling. Holotype: male, USNM. 

**Material examined.** Taiwan: holotype: ♂, Nantow Co. Mei-feng, 30 km S Tayuling, 2200 m, 1-8-VI-1980, D. R. Davis (USNM92895, A. Kawabe) (examined) [USNM].

1 ♂, Sikikun, 7-VIII-43, A. Mutuura (Sun-220 ♂); 1 ♀, Daibusan, 15-III-27, S. Issiki (Sun-338 ♀, abdomen broken) [USNM].

1 ♂, Taitung Co. Yakou, 2750 m, 25-X-1984, J. Heppner and H. Wang [FSCA].

**Distribution.** China (Taiwan).

#### 3.1.2. *Apotomis fuscomaculata* Kawabe, 1993 

*Apotomis fuscomaculata* Kawabe, 1993: 230. TL: Taiwan (Taipei Hsien, Chihsingshan, ca 10 air km N Taipei). Holotype: male, USNM.

**Material examined.** Taiwan: holotype: ♂, Taipei Co. Chihsingshan, ca. 10 air km N Taipei, 700 m, 28-VII-1980, D. R. Davis (USNM92897, A. Kawabe) (examined) [USNM].

3 ♂♂, 1 ♀, Taipei Co. Chihsingshan, ca. 10 air km N Taipei, 700 m, 28-VII-1980, D. R. Davis (Sun-221 ♂, USNM92896, A. Kawabe); 1 ♂, Taiwan, Sozan, 7-VII-1940, S. Issiki [USNM]. 

1 ♂, Hsinchu Co. Kuangwu For. Sta., 2000 m, 18-25-VIII-1988, J. Heppner and H. Wang; 1 ♂, Taipei Co. Wulai, 200 m, 17-19-VI-1985, J. Heppner and H. Wang (Gen. Sl. Bae UFL-040 ♂); 1 ♀, Nantou Co., Meifeng, 2100 m, 6-9-IX-1988, J. Heppner (TORT12013 ♀, FL198) [FSCA].

**Distribution.** China (Taiwan).

#### 3.1.3. *Apotomis platycremna* (Meyrick, 1935)

*Argyroploce platycremna* Meyrick, in Caradja and Meyrick, 1935: 61. TL: China, West Tienmushan. Holotype: male, BMNH. 

*Olethreutes platycremna*: Clarke, 1958: 536.

*Apotomis platycremna* (Meyrick): Diakonoff, 1973: 524.

**Material examined.** Taiwan: 1 ♂, Miaoli Hsien, Hernglong Lodge, 550 m, 8-VIII-1983, A. Kawabe; 1 ♂, Nantou Hsien, Nanshanchi, 25-26-VII-1983, A. Kawabe (Sun-222 ♂, USNM145132, YH Sun); 2 ♂♂, Nantou Hsien, Lushan spa, 1200 m, 27-29-VII-1983, A. Kawabe; 1 ♂, Nantou Hsien, Wushe, 1000 m, 6-7-VIII-1985, A. Kawabe; 6 ♂♂, Taiwan, Kansirei, 19-X-1934, S. Issiki [USNM].

Gansu: 23 ♂♂, 1 ♀, Bifenggou, Wen County, 860 m, 9~13-VII-2005, Haili Yu. Guizhou: 10 ♂♂, Fangxiang Village, Leishan County, 900 m, 13~14-IX-2005, Jialiang Zhang; 4 ♂♂, Xiaodan Village, Rongjiang County, 680 m, 15~16-IX-2005, Jialiang Zhang; 1 ♂, Heiwan River, Jiangkou County, 600 m, 28-VII-2001, Houhun Li and Xinpu Wang. Hainan: Mt. Limu: Forest Park (19.17° N, 109.73° E), 607 m: 1 ♀, 13-V-2015, Peixin Cong, Wei Guan, and Sha Hu; 1 ♂, 22-X-2016, Xia Bai, Shuonan Qian, and Wanding Qi. Mt. Yingge: 1 ♂, Nankai Station (19.07° N, 109.40° E), 210 m, 11-VIII-2016, Xia Bai, Shuonan Qian, and Wanding Qi; 5 ♂♂, 2 ♀♀, Yinggezu (19.05° N, 109.57° E), 599 m, 27~31-VII-2017, Xia Bai, Ping Liu, and Shuai Yu. Wuzhishan City: 1 ♀, Lizudadian (18.88° N, 109.67° E), Shuiman Town, 766 m, 5-VII-2015, Qingyun Wang, Suran Li, and Mengting Chen (BX15287); 1 ♂, Butterfly Ranch (18.87° N, 109.67° E), 680 m, 27-X-2016, Xia Bai, Shuonan Qian, and Wanding Qi (BX15396); 1 ♂, National Park (18.88° N, 109.67° E), 766 m, 9-I-2016, Kaijian Teng, Xia Bai, and Mengting Chen; 1 ♂, Wuzhishan Nature Reserves (18.88° N, 109.65° E), 742 m, 3-VII-2014, Peixin Cong, Linjie Liu, and Sha Hu. Henan: 2 ♂♂, Shuiliandong, Tongbai County, 300 m, 26-V-2000, Haili Yu. Fujian: 1 ♂, weather station, Jianning County, 350 m, 25-IX-2002, Xinpu Wang; 2 ♂♂, Mt. Daiyun, Dehua County, 850~1350 m, 2~15-IX-2002, Xinpu Wang. Hunan: 1 ♂, Cangxi Town, Xinhua County, 8-VIII-2004, Yunli Xiao. Zhejiang: 2 ♂♂, Mt. Tianmu, 350~1140 m, 15~17-VIII-1999, Houhun Li [NKU].

**Distribution.** China (Fujian, Gansu, Guizhou, Hainan, Henan, Hunan, Taiwan, Zhejiang) and Japan.

### 3.2. Arcesis *Diakonoff, 1983*

*Arcesis* Diakonoff, 1983: 49. 

Type-species: *Arcesis anax* Diakonoff, 1983.

#### 3.2.1. *Arcesis threnodes* (Meyrick, 1905)

*Platypeplus hemiopta* Meyrick, 1905: 586. TL: Sri Lanka. Ceylon [Sri Lanka] (Peradeniya). Holotype: male, BMNH. 

*Platypeplus threnodes* Meyrick, 1905: 585. TL: Sri Lanka, Ceylon [Sri Lanka] (Kandy). Holotype: male, BMNH [lost]. 

*Platypeplus hemiopta* (Meyrick): Meyrick, 1905: 586.

*Platypeplus tetracona* Meyrick, 1907: 731. TL: Sri Lanka, Ceylon [Sri Lanka] (Maskeliya). Holotype: male, BMNH. 

*Olethreutes threnodes* (Meyrick): Clarke, 1958: 555.

*Eucosma threnodes* (Meyrick): Diakonoff, 1982: 43.

*Statherotis threnodes* (Meyrick): Kawabe, 1982: 167.

*Arcesis threnodes* (Meyrick): Kuznetzov, 2001: 282.

**Material examined.** Taiwan: 1 ♂, 1 ♀, Chiai Hsien, Fenchihu, 1400 m, 11-12-VIII-1985, A. Kawabe (Sun-223 ♂; Sun-224 ♀); 1 ♀, Zhanghua Co., Dec. 20, 1995, Y. F. Hsu [USNM]. 

1 ♂, Taoyuan Co. Upper Palin, Lalashan, 1500 m, 11-18-VII-1996, J. Heppner and H. Wang; 1 ♂, Taoyuan Co. Balin, U., 1000 m, 12-VIII-1992, T.T.Lo; 3 ♂♂, Taipei Co. Wulai, 200 m, 17-19-VI-1985, J. Heppner and H. Wang; 2 ♂♂, 1 ♀, Nantou Co. Meifeng Hort. Sta., 2100 m, 6-10-IX-1988, J. Heppner and H. Wang [FSCA].

**Host plants.** Magnoliaceae: *Michelia* spp. and *Magnolia* spp.

**Distribution.** China (Fujian, Guangdong, Guizhou, Hainan, Taiwan, Yunnan), Japan, Russia, and Sri Lanka.

### 3.3. Archilobesia *Diakonoff, 1966*

*Lobophora* Turner, 1946: 214. Type-species: *Lobophora axiologa* Turner, 1946 [preoccupied].

*Archilobesia* Diakonoff, 1966: 45. Type-species: *Argyroploce drymoptila* Lower, 1920.

*Enveryucella* Kocak, 1981: 118 [replacement name for *Lobophora*]. 

#### 3.3.1. *Archilobesia drymoptilia* (Meyrick, 1933)

*Argyroploce drymoptila* Lower, 1920: 58. TL: Australia (Queensland, Cairns). Lectotype: male, SAMA. 

*Argyroploce crossoleuca* Meyrick, 1933: 420. TL: Indonesia (Java). Lectotype: female, BMNH.

*Olethreutes crossoleuca*: Clarke, 1958: 500.

*Archilobesia drymoptila*: Diakonoff, 1966: 45.

**Material examined.** Taiwan: 1 ♂, Taihoku, 27-VI-1933, S. Issiki (Sun-225 ♂, USNM145133, YH Sun); 1 ♂, Taihoku, 5-V-1946, S. Issiki; 1 ♀, Taihoku, 4-V-1946, S. Issiki (Sun-226 ♀, USNM145134, YH Sun); 1 ♂, Taipei Co. Chihsingshan, ca. 10 air km N Taipei, 700 m, 28-VII-1980, D. R. Davis; 1 ♂, (Wurai) Taipei Hsien, Formosa, 30-VII-1974, Y. Kishida; 1 ♂, Nantou Hsien, Lushan spa, 1200 m, 27-29-VII-1983, A. Kawabe; 1 ♂, Taiwan, Taihoku, 18-X-1923, S. Issiki [USNM].

**Host plant.** Phyllanthaceae: *Glochidion* sp.

**Distribution.** China (Fujian, Taiwan), Indonesia, and Australia.

**Remarks.** This species is recorded for the first time in Taiwan.

#### 3.3.2. *Archilobesia formosana* Diakonoff, 1973

*Archilobesia formosana* Diakonoff, 1973: 403. TL: Taiwan, Formosa [Taiwan] (Taihoku). Holotype: female, BMNH. 

**Distribution.** China (Taiwan).

### 3.4. Aterpia *Guenée, 1845*

*Aterpia* Guenée, 1845: 161. Type-species: *Aterpia anderreggana* Guenée, 1845. 

*Asaphistis* Meyrick, 1909: 590. Type-species: *Asaphistis praeceps* Meyrick, 1909. 

*Esia* Heinrich, 1926: 109. Type-species: *Olethreutes approximana* Heinrich, 1919. 

*Apeleptera* Diakonoff, 1973: 262. Type-species: *Argyroploce semnodryas* Meyrick, 1936. 

*Leptocera* Diakonoff, 1983: 307. Type-species: *Gnathmocerodes (Leptocera) microchlamys* Diakonoff 1983 [described as subgenus of *Gnathmocerodes*].

#### 3.4.1. *Aterpia semnodryas* (Meyrick, 1936)

*Argyroploce semnodryas* Meyrick, 1936: 613. TL: Taiwan, Formosa [Taiwan] (Taihoku). Holotype: male, BMNH. 

*Olethreutes semnodryas* (Meyrick): Clarke, 1958: 548.

*Apeleptera semnodryas* (Meyrick): Diakonoff, 1973: 263.

*Aterpia semnodryas* (Meyrick): Horak, 2006: 199.

**Material examined.** Taiwan: 1 ♀, Taipei Hsien, Wulai, 400 m, 14-VIII-1985, A. Kawabe (Sun-227 ♀, USNM145135, YH Sun) [USNM]. 

8 ♂♂, Taipei Co. Wulai, 200 m, 17-19-VI-1985, J. Heppner and H. Wang (TORT11980 ♂, FL138; Gen. Sl. Bae UFL-105 ♂); 1 ♂, Ilan Co. Chilan Nsy., Tuchan, 480 m, 15-17-X-1984, J. Heppner and H. Wang; 1 ♂, Ilan Co. 5 km N. Nanao, 100 m, 11-13-X-1984, J. Heppner and H. Wang [FSCA].

**Distribution.** China (Taiwan), Japan, and Russia.

### 3.5. Cephalophyes *Diakonoff, 1973*

*Cephalophyes* Diakonoff, 1973: 201. Type-species: *Cephalophyes porphyrea* Diakonoff, 1973.

#### 3.5.1. *Cephalophyes cyanura* (Meyrick, 1909)

*Cephalophyes cyanura* (Meyrick): Diakonoff, 1973: 204.

*Argyroploce cyanura* Meyrick, 1909: 598. TL: Malay Peninsula, Malay Peninsula (Gunong Ijan). Lectotype: male, BMNH. 

**Material examined.** Taiwan: 1 ♀, Hassenzan, 4-VI-1947, S. Issiki; 1 ♀, Mt. Kisisima,3-V-1929, S. Issiki; 11 ♂♂, 5 ♀♀, Chiai Hsien, Alishan, 2270 m, 8-10-VIII-1985, A. Kawabe (Sun-228 ♂, USNM122536, A. Kawabe; Sun-229 ♂; Sun-230 ♀, USNM145136, YH Sun); 1 ♂, Chiai Hsien, Fenchihu, 1400 m, 11-12-VIII-1985, A. Kawabe; 1 ♂, Hualien Hsien, Hohuanshan, 3100 m, 30-VII-1-VIII-1983, A. Kawabe; 1 ♀, Hualien Hsien, Tayuling, 2560 m, 12-VI-1988, K. Fujisawa; 1 ♂, Nantou Hsien, Wushe, 1150 m, 27-XII-1988, A. Kawabe; 1 ♀, Taiwan, Sinten, 17-III-26, S. Issiki; 1 ♀, Taiwan, Rarasan, 28-VI-43, S. Issiki; 1 ♀, (Alishan) Chiai Hsien, Formosa, 12-VIII-1974, Y. Kishida; 1 ♀, Taiwan, Rantaisan, 21-V-28, S. Issiki [USNM].

**Distribution.** China (Fujian, Taiwan), India, Japan, Malaysia, and Russia.

### 3.6. Cimeliomorpha *Diakonoff, 1966*

*Cimeliomorpha* Diakonoff, 1966: 50. Type-species: *Copromorpha cymbalora* Meyrick, 1907.

**Remarks.** No species had been recorded in China prior to this study.

#### 3.6.1. *Cimeliomorpha cymbalora* (Meyrick, 1907) 

*Copromorpha cymbalora* Meyrick, 1907: 152. TL: India, Assam, Khasi Hills. Lectotype: male, BMNH. 

*Cimeliomorpha cymbalora* (Meyrick): Brown, 2005: 177.

**Material examined.** Taiwan: 1 ♂, 2 ♀♀, Baibara, 25-III-43, S. Issiki (Sun-231 ♂, USNM145137, YH Sun; Sun-232 ♀, USNM145138, YH Sun) [USNM]. 

1 ♀, Nantou Co. 12 km E. Puli/cyn., 700 m, 24-25-III-1990, J. Heppner and H. Wang [FSCA].

**Distribution.** China (Taiwan) and India.

**Remarks.** This species is recorded for the first time in China.

### 3.7. Costosa *Diakonoff, 1968*

*Costosa* Diakonoff, 1968: 80. Type-species: *Costosa allochroma* Diakonoff, 1968.

*Rhodonympha* Diakonoff, 1968: 71. Type-species: *Eucosma rhodantha* Meyrick, 1907. 

#### 3.7.1. *Costosa rhodantha* (Meyrick, 1907)

*Eucosma rhodantha* Meyrick, 1907: 138. TL: India, Assam, Khasi Hills. Holotype: male, BMNH. 

*Olethreutes rhodantha* (Meyrick): Clarke, 1958: 543.

*Rhodonympha rhodantha* (Meyrick): Diakonoff, 1967: 74.

*Costosa rhodantha* (Meyrick): Diakonoff, 1973: 306.

**Material examined.** Taiwan: 1 ♀, Raisya, 23-XI-1934, S. Issiki, no abdomen; 1 ♂, Chaiyi Hsien, Fennchihhu, ca. 1400 m, 16-VII-1984, Y. Arita; 1 ♂, Nantou Hsien, Nanshanchi, 25-26-VII-1983, A. Kawabe (Sun-233 ♂, USNM122537, A. Kawabe); 1 ♂, Nantou Hsien, Lushan spa, 1200 m, 7-8-VI-1988, K. Fujisawa (Sun-234 ♂) [USNM]. 

1 ♂, Taitung Co. Bishan Hot Spgs., 720 m, 26-29-X-1984, J. Heppner and H. Wang (TORT12156 ♂) [FSCA].

**Distribution.** China (Taiwan), India, and Sri Lanka.

### 3.8. Dactylioglypha *Diakonoff, 1973*

*Dactylioglypha* Diakonoff, 1973: 188. Type-species: *Dactylioglypha avita* Diakonoff, 1973.

#### 3.8.1. *Dactylioglypha tonica* (Meyrick, 1909)

*Argyroploce tonica* Meyrick, 1909: 606. TL: Sri Lanka, Ceylon [Sri Lanka] (Kandy). Lectotype: male, BMNH. 

*Lipsotelus tonica* (Meyrick): Issiki, 1957: 68.

*Olethreutes tonica* (Meyrick): Clarke, 1958: 556.

*Dactylioglypha tonica* (Meyrick): Diakonoff, 1973: 190.

**Material examined.** Taiwan: 1 ♂, Sirin, 27-X-1933, S. Issiki (Sun-235 ♂); 1 ♂, Shiraki; 1 ♂, Taiwan, Taihoku, 6-XI-1923, S. Issiki; 1 ♂, Nantou Hsien, Lushan spa, 1200 m, 3-5-VIII-1985, A. Kawabe; 1 ♂, Nantou Hsien, Lushan spa, 1200 m, 27-29-VII-1983, A. Kawabe; 1 ♂, Taiwan, Heito, 24-VIII-1933, S. Issiki [USNM]. 

1 ♂, Kaohsiung Co. Dona, 16 km NE. Maolin, 500 m, 3-XI-1984, J. Heppner and H. Wang (TORT11834 ♂, FL108) [FSCA].

Hainan: 1 ♂, Mt. Limu (19.17° N, 109.73° E), 700 m, 13-IV-2008, Bingbing Hu and Haiyan Bai; 1 ♂, Nancha River, Mt. Bawang (19.22° N, 109.43° E), 600 m, 10-VI-2007, Zhiwei Zhang and Weichun Li; 1 ♂, Dongfang City, Datian Nature Reserves (19.11° N, 108.79° E), 100 m, 28-IV-2009, Qing Jin and Bingbing Hu (BX15431) [NKU].

**Distribution.** China (Hainan, Taiwan), India, Indonesia, Japan, Malaysia, and Sri Lanka.

### 3.9. Dicephalarcha *Diakonoff, 1973*

*Dicephalarcha* Diakonoff, 1973: 254. Type-species: *Dicephalarcha sicca* Diakonoff, 1973.

#### 3.9.1. *Dicephalarcha dependens* (Meyrick, 1922)

*Argyroploce dependens* Meyrick, 1922: 524. TL: China, Shanghai. Holotype: male, MNHN. 

*Dicephalarcha dependens* (Meyrick): Diakonoff, 1973: 256.

**Material examined.** Taiwan: 1 ♂, 1 ♀, Raisya, 23-XI-1934, S. Issiki; 1 ♂, 1 ♀, Taiwan, Taihoku, 20-XI-1940, S. Issiki (Sun-299 ♂); 1 ♂, Chiai Hsien, Alishan, 2270 m, 8-10-VIII-1985, A. Kawabe; 1 ♂, Chiai Hsien, Fenchihu, 1400 m, 11-12-VIII-1985, A. Kawabe; 1 ♂, Nantow Co. Lienhuachi Exp. For. Sta. 15 km SW Puli, 750 m, 22-26-V-1980, D. R. Davis [USNM]. 

2 ♀♀, Taichung Co. Chingshan, 1100 m, 31-VIII/4-IX-1988, J. Heppner and H. Wang (TORT11999 ♀, FL189); 1 ♀, Taipei Co. Wulai, 200 m, 17-19-VI-1985, J. Heppner and H. Wang; 3 ♀♀, Taoyuan Co. Upper Palin, 1500 m, 7-9-VII-1985, J. Heppner and H. Wang; 1 ♀, Kaohsiung Co. Liukuei, Shanpin For., 750 m, 16-23-III-1990, J. Heppner and H. Wang (TORT12000 ♀); 1 ♀, Ilan Co. Tuchan, 480 m, 1-2-VII-1982, J. Heppner (Gen. Sl. Bae UFL-012 ♀); 1 ♀, Ilan Co. Fushan Botanical Sta., 650 m, 20-24-VII-1996, J. Heppner [FSCA].

Anhui: 2 ♂♂, Tanqiao Town, Huangshan City, 4~6-VIII-2004, Jiasheng Xu and Jialiang Zhang; 2 ♀♀, Mozitan Town, Huoshan County, 12-VIII-2004, Jiasheng Xu and Jialiang Zhang; 1 ♀, Mt. Tianzhu, 10-VIII-2004, Jiasheng Xu and Jialiang Zhang; 1 ♀, Ke Village, Mt. Jiuhua, 8-VIII-2004, Jiasheng Xu and Jialiang Zhang. Hainan: 1 f #, Mt. Limu (19.17° N, 109.74° E), 700 m, 13-IV-2008, Bingbing Hu and Haiyan Bai; 5 ♂♂, 2 ♀♀, Mt. Limu Nature Reserves (19.17° N, 109.74° E), 640‒700 m, 1~4-V-2014, Tengteng Liu, Wei Guan, and Xuemei Hu (BX15241 ♀, BX15242 ♂); 1 ♂, Forest Park, Mt. Limu (19.17° N, 109.73° E), 607, 15-V-2015, Peixin Cong, Wei Guan, and Sha Hu; 1 ♀, Mt. Yingge (19.08° N, 109.50° E), 620 m, 21-VI-2010, Bingbing Hu and Jing Zhang; 1 ♂, 1 ♀, Mt. Jianfeng (18.75° N, 108.87° E), 810 m, 13~15-VI-2018, Ping Liu, Xia Bai, and Shuai Yu; 1 ♂, Tianchi (18.74° N, 108.84° E), 1050 m, 29-IV-2013, Yinghui Sun, Wei Guan, and Tengteng Liu. Hubei: 1 ♂, Mahe Town, Xianfeng City, 400 m, 24-VII-1999, Houhun Li. Fujian: 24 ♂♂, 9 ♀♀, Sangang, Mt. Wuyi, 740 m, 20~24-V-2004, Haili Yu. Yunnan: 1 ♂, Tropical Botanical Garden, Menglun Town, 570 m, 13-VIII-2005, Yingdang Ren; 1 ♂, 3 ♀♀, Suoluo National Nature Reserve, Chishui City, 240 m, 21~23-IX-2000, Haili Yu; 3 ♀♀, Suoluo National Nature Reserve, Chishui City, 390 m, 29-V-2000, Yanli Du [NKU].

**Distribution.** China (Anhui, Hainan, Fujian, Guizhou, Jiangsu, Shanghai, Sichuan, Taiwan, Yunnan).

#### 3.9.2. *Dicephalarcha herbosa* (Meyrick, 1909) 

*Argyroploce herbosa* Meyrick, 1909: 600. TL: India, Assam, Khasi Hills. Lectotype: male, BMNH. 

*Argyroploce mniopyrrha* Meyrick, 1931: 134. TL: Indonesia. Java, Buitenzorg. Holotype: female, BMNH. 

*Dicephalarcha herbosa* (Meyrick): Brown, 2005: 246.

**Material examined.** Taiwan: 1 ♀, Taihoku, 5-VI-1946, S. Issiki (Sun-330 ♀); 1 ♀, Taiwan, Raisya, 23-XI-1934, S. Issiki (Sun-331 ♀); 1 ♀, Taiwan, Taihoku, 23-X-1934, S. Issiki; 1 ♂, Chiai Hsien, Alishan, 2270 m, 8-10-VIII-1985, A. Kawabe (Sun-237 ♂, USNM122461, A. Kawabe); 1 ♀, Chiai Hsien, Fenchihu, 1400 m, 11-12-VIII-1985, A. Kawabe (Sun-238 ♀, USNM122462, A. Kawabe) [USNM]. 

1 ♀, Miaoli Co. Taian Hot Spr., 600 m, 27-28-VI-1985, J. Heppner and H. Wang (TORT11983 ♀, FL128); 1 ♀, Ilan Co. Mingchyr Rec. Area, 1250 m, 9-10-VII-1996, J. Heppner and H. Wang [FSCA].

Hainan: 1 ♂, Mt. Wolong, Tunchang County (19.46° N, 110.12° E), 126 m, 20-VI-2018, Xia Bai, Ping Liu, and Shuai Yu; 1 ♂, 1 ♀, Forest Park, Mt. Limu (19.17° N, 109.73° E), 607 m, 12~13-V-2015, Peixin Cong, Linjie Liu, and Sha Hu (BX15270 ♀); 1 ♂, Hongxin Village, Yuanmen Town, Baisha County (19.07° N, 109.52° E), 445 m, 17-IV-2014, Tengteng Liu, Wei Guan, and Xuemei Hu (BX16432); 1 ♂, Mt. Diaoluo (18.74° N, 109.84° E), 100 m, 18-IV-2008, Bingbing Hu and Haiyan Bai; 1 ♀, Mt. Jianfeng Nature Reserves (18.74° N, 108.84° E), 1050 m, 27-IV-2014, Tengteng Liu, Wei Guan, and Xuemei Hu (BX16491); 1 ♂, Tianchi (18.73° N, 108.87° E), 787 m, 9-III-2016, Qingyun Wang, Suran Li, and Shengnan Zhao (BX15537) [NKU].

**Distribution.** China (Hainan, Taiwan), India, and Indonesia.

**Remarks.** This species is recorded for the first time in China.

#### 3.9.3. *Dicephalarcha sicca* (Diakonoff, 1973)

*Dicephalarcha sicca* Diakonoff, 1973: 256. TL: Indonesia, West Java, Gede−Pangrango Mountains, Tjibodas. Holotype: male, RMNH. 

*Dicephalarcha sicca* (Diakonoff): Kawabe, 1992: 106.

**Material examined.** Taiwan: 1 ♀, Raisya, 23-XI-1934, S. Issiki (Sun-236 ♀) [USNM]. 

1 ♀, Kaohsiung Co. Liukuei, Shanpin For., 750 m, 16-23-III-1990, J. Heppner and H. Wang (TORT12059 ♀, FL241) [FSCA].

**Distribution.** China (Taiwan) and Indonesia.

### 3.10. Dudua *Walker, 1864*

*Dudua* Walker, 1864: 1000. Type-species: *Dudua hesperialis* Walker, 1864.

*Platypeplus* Walsingham, 1887: 495. Type-species: *Eccopsis aprobola* Meyrick, 1886. 

*Platypeplum* Walsingham, 1899: 105 [unjustified emendation *Platypeplus*]. 

#### 3.10.1. *Dudua aprobola* (Meyrick, 1886)

*Eccopsis aprobola* Meyrick, 1886: 275. TL: Tonga. Holotype: male, BMNH. 

*Platypeplus aprobola* (Meyrick): Walsingham, 1887: 495.

*Temnolopha metallota* Lower, 1901: 73. TL: Australia. Queensland, Cocktown. Syntype: male, SAMA. 

*Argyroploce aprobola* (Meyrick): Meyrick, 1911: 275.

*Hedya (Platypeplus) aprobola* (Meyrick): Diakonoff, 1968: 46. 

*Dudua aprobola* (Meyrick): Diakonoff, 1971: 191.

*Dudua aprobola kusaiensis* Clarke, 1976: 86. TL: Micronesia. Micronesia (Kusaie, Mutunlik). Holotype: male, USNM. 

**Material examined**. Taiwan: 1 ♂, Raisya, 23-XI-1934, S. Issiki; 1 ♂, Raisya, 25-XI-1934, S. Issiki; 1 ♂, Kansirei, 17-X-1934, S. Issiki; 1 ♂, Kansirei, 18-X-1934, S. Issiki; 1 ♂, Heito, 8-IV-1935, S. Issiki; 1 ♂, Heito, 26-XII-1934, S. Issiki (Sun-241 ♂); 1 ♀, Kagi, 13-IV-1935, S. Issiki (Sun-242 ♀); 1 ♀, Hassenzan, 25-X-1929, S. Issiki; 3 ♂♂, Chiai Hsien, Fenchihu, 1400 m, 11-12-VIII-1985, A. Kawabe; 2 ♂♂, 4 ♀♀, Nantou Hsien, Lushan spa, 1200 m, 3-5-VIII-1985, A. Kawabe; 1 ♂, Hualien Hsien, Hungyeh spa, 200 m, 3-I-1989, A. Kawabe (Sun-240 ♂); 1 ♂, Hualien Hsien, Hungyeh spa, 200 m, 29-30-III-1984, A. Kawabe (Sun-239 ♂, USNM122538, A. Kawabe); 3 ♂♂, 1 ♀, Chihpen, Taitung Hsien, Formosa, 16-VIII-1974, H. Nakajima; 3 ♂♂, 1 ♀, Nantow Co. Sunmoon Lake, 760 m, 20-25-VI-1980, D. R. Davis; 1 ♀, Nantou Hsien, Lushan spa, 1200 m, 27-29-VII-1983, A. Kawabe; 1 ♀, TaiNanCo. 2-3 km S Kwantzuling, 26-28-VI-1980, D. R. Davis; 1 ♂, (Wurai) Taipei Hsien, Formosa, 30-VII-1974, Y. Kishida; 1 ♂, (Lushan spa) Nantow Hsien, Formosa, 6-VIII-1974, Y. Kishida; 1 ♂, Kao Hsiung Co., 10-11 km NE Chiahsien, 300 m, 3-8-VII-1980, D. R. Davis; 1 ♂, Oomura, Chichi-jima, 28-IV-1978, Y. Kusui; 1 ♂, Chiai Hsien, Fenchihu, 1400 m, 11-12-VIII-1985, A. Kawabe; 1 ♂, Taiwan, Haisya, 23-XI-1934, S. Issiki [USNM]. 

1 ♂, Ilan Co. Fushan For. Sta., 650 m, 4-11-IV-1990, J. Heppner; 2 ♂♂, 1 ♀, Kaohsiung Co. Liukuei, Shanpin For., 750 m, 16-23-III-1990, J. Heppner and H. Wang (Gen. Sl. Bae UFL-015 ♂); 1 ♂, Taitung Co. Bishan Hot Spgs., 720 m, 26-29-X-1984, J. Heppner and H. Wang (Gen. Sl. Bae UFL-025 ♂); 1 ♂, 1 ♀, Taitung Co. Bishan Hot Spgs., 720 m, 26-29-X-1984, J. Heppner and H. Wang; 1 ♂, 2 ♀♀, Taipei Co. Wulai, 200 m, 17-19-VI-1985, J. Heppner and H. Wang; 1 ♀, Ilan Co. 5 km N. Nanao, 100 m, 11-13-X-1984, J. Heppner and H. Wang; 1 ♀, Nantou Co. 15 km E of Puli, 700 m, 6-V-1989, J. Heppner and H. Wang; 1 ♀, Nantou Co. Lo Farm, 8 km E. Puli, 750 m, 10-11-VII-2005, J. Heppner; 2 ♂♂, Pingtung Co. Kenting Park, 50 m, 31-X/1-XI-1984, J. Heppner and H. Wang; 4 ♂♂, Nantou Co.5 km N. Chitou, 500 m, 29-VI-1985, J. Heppner and H. Wang (Gen. Sl. Bae UFL-016 ♂; Gen. Sl. Bae UFL-018 ♂); 1 ♂, Pingtung Co. Kenting Park, 50 m, 1-5-IX-1983, J. Heppner; 1 ♂, Taitung Co., Chihpen Hot Springs, 9-VIII-1992, J. Heppner; 1 ♂, Ilan Co. 6 km S. Suao, 100 m, 14-X-1984, J. Heppner; 1 ♂, Kaohsiung Co. Dona, 16 km NE. Maolin, 500 m, 3-XI-1984, J. Heppner and H. Wang; 3 ♂♂, 2 ♀♀, Ilan Co. 5 km N. Nanao, 100 m, 11-13-X-1984, J. Heppner and H. Wang; 1 ♂, Ilan Co. Tuchan, 480 m, 1-2-VII-1982, J. Heppner; 1 ♂, Nantou Co. Tungpu, 1150 m, 1-VII-1985, J. Heppner and H. Wang (Gen. Sl. Bae UFL-017 ♂) [FSCA].

Guangdong: 4 ♂♂, 4 ♀♀, Mt. He, 5-IX~17-XII-2002, Guilin Liu and Binglan Zhang; 1 ♂, Pinglang Town, Heng County, 18-VII-2002, Yanli Du. Hainan: Haikou City: 1 ♂, Jinniuling Park (20.00° N, 110.31° E), 23 m, 31-V-2018, Ping Liu, Xia Bai, and Shuai Yu. Tunchang County: 1 ♀, Mt. Wolong (19.46° N, 110.12° E), 126 m, 21-VI-2018.VI.21, Ping Liu, Xia Bai, and Shuai Yu. Ligao County: 4 ♂♂, 18 ♀♀, Mt. Gaoshan (19.93° N, 109.64° E), 156 m, 30-XII-2017~3-VI-2018, Mujie Qi et al. (BX15838 ♂, BX15839 ♀, BX15840 ♀, BX16399 ♀). Mt. Limu: 3 ♂♂, 1 ♀, Forest Park (19.17° N, 109.73° E), 607 m, 25-VII-2014~23-X-2016, Peixin Cong et al. (BX15748); 1 ♂, Mt. Limu Nature Reserves (19.17° N, 109.74° E), 640~700 m, 1-V-2014, Tengteng Liu, Wei Guan, and Xuemei Hu. Mt. Bawang: 3 ♂♂, 4 ♀♀, Yajia Secondary Hydropower Station (19.08° N, 109.10° E), 261 m, 19~21-VII-2015, Qingyun Wang, Suran Li, and Mengting Chen (BX15393 ♀); 2 ♀♀, Nancha River (19.22° N, 109.43° E), 600 m, 9-VI-2007, Zhiwei Zhang and Weichun Li (BX15757). Dongfang City: 4 ♂♂, 29 ♀♀, Datian Nature Reserves (19.11° N, 108.79° E), 25~100 m, 26-IV-2009~6-VI-2018, Qingjin et al. (BX16401 ♀); 6 ♀♀, Lemei Village (19.08° N, 108.84° E), 81 m~124 m, 3-I~8-VI-2018, Mujie Qi et al. (BX16400). Mt. Yingge: 2 ♀♀, Mt. Yingge (19.08° N, 109.50° E), 620 m, 23-V-2010, Bingbing Hu and Jing Zhang; 9 ♂♂, 4 ♀♀, Hongkan Ranger Station (19.08° N, 109.50° E), 508~540 m, 26-VI-2014~26-VII-2015, Peixin Cong et al. (BX15132 ♂); 1 ♂, 2 ♀♀, Nankai Station (19.07° N, 109.40° E), 210~280 m, 16-VIII-2016~9-V-2017, Xia Bai et al. Baisha County: 15 ♂♂, 4 ♀♀, Hongxin Village, Yuanmen Towan (19.08° N, 109.52° E), 430~460 m, 14-IV-2014~22-III-2016, Tengteng Liu et al. (BX15391 ♂, BX15394); 6 ♂♂, 3 ♀♀, Yaxing Village, Nankai Towan (19.02° N, 109.40° E), 321 m, 20-VI~30-VII-2015, Peixin Cong et al. (BX15415 ♂, BX15136). Wuzhishan City: 2 ♂♂, 2 ♀♀, Maoyang Town (18.94° N, 109.51° E), 225 m, 19-IV-2009, Qing Jin and Bingbing Hu; 3 ♂♂, 2 ♀♀, Shuiman Town (18.88° N, 109.66° E), 430~640 m, 19-IV-2014~16-V-2017, Tengteng Liu et al.; 9 ♂♂, 5 ♀♀, Nature Reserves (18.88° N, 109.67° E), 738~766 m, Kaijian Teng et al. (BX15384, BX15413, BX15758). Mt. Diaoluo: 1 ♀, Nanxi Protection Station (18.66° N, 109.93° E), 300 m, 12-VIII-2008, Bingbing Hu and Li Zhang (BX15759); 1 ♂, Nature Reserves (18.43° N, 109.52° E), 922 m, 24-V-2015, Peixin Cong, Wei Guan, and Sha Hu (BX15133). Ledong County: 2 ♂♂, 2 ♀♀, Jianfeng Town (18.70° N, 108.79° E), 40 m, 28-VI~2-V-2013, Yinghui Sun, Wei Guan, and Tengteng Liu; 24 ♂♂, 7 ♀♀, Tropical Botanical Garden (18.70° N, 108.79° E), 40 m, 25~30-IV-2014, Tengteng Liu, Wei Guan, and Xuemei Hu (BX15134 ♀, BX15135 ♀); Mt. Jianfeng: 1 ♂, Mt. Jianfeng (18.75° N, 108.87° E), 810 m, 5-VI-2018, Ping Liu, Xia Bai, and Shuai Yu; 2 ♂♂, Tianchi (18.73° N, 108.87° E), 787 m, 13~14-VII-2015, Qingyun Wang, Suran Li, and Mengting Chen (BX15147); 4 ♂♂, Nature Reserves (18.74° N, 108.84° E), 1050 m, 27-IV-2014, Tengteng Liu, Wei Guan, and Xuemei Hu. Yunnan: 1 ♀, The Institute of Medicinal Plant Development, Jinghong City, 585 m, 18-IV-1995, Hongjian Wang; 1 ♂, Bubang Village, Mengla County, 650 m, 22-VIII-2005, Yingdang Ren; 1 ♂, Guanping Village, Mengyang Town, 1200 m, 18-VIII-2005, Yingdang Ren [NKU].

**Host plants.** Malvaceae: *Abelmoschus moschatus* Medicus; Anacardiaceae: *Mangifera indica* Linn. and *Anacardium occidentale* Linn.; Fabaceae: *Flemingia macrophylla* (Wiild.), *Inocarpus fagifer* (Parkinson), and *Arachis hypogaea* Linn.; Asteraceae: *Bidens pilosa* Linn. and *Dahlia* spp.; Lauraceae: *Rhodomyrtus tomentosa* (Ait.), *Eugenia aquea* Burm, *E. jambos* Linn., *Eucalyptus* spp., *Metrosideros collina* Tahiti., and *Cinnamomum zeylanicum* Blume; Lythraceae: *Lagerstroemia speciosa* (Linn.); Moraceae: *Ficus* spp.; Verbenaceae: *Lantana camara* Linn.; Loranthaceae: *Loranthus* spp.; Sapindaceae: *Litchi chinensis* Sonn.; Gramineae: *Oryza glaberrima* Steud.; Plumbaginaceae: *Plumbago zeylanica* Linn.; Myrtaceae: *Psidium guajava* Linn.; Annonaceae: *Polyalthia longifolia* Benth. et Hook.; Rosaceae: *Rosa* spp.

**Distribution.** China (Guangdong, Guangxi, Hainan, Taiwan, Yunnan), Australia, Brazil, East Africa, East Asia, and South Asia.

#### 3.10.2. *Dudua charadraea* (Meyrick, 1909)

*Argyroploce charadraea* Meyrick, 1909: 594. TL: Sri Lanka, Ceylon [Sri Lanka] (Maskeliya). Lectotype: male, BMNH. 

*Argyroploce ptarmicopa* Meyrick, 1936: 612.

*Olethreutes ptarmicopa* (Meyrick): Inoue, 1954: 105.

*Platypeplus charadraea* (Meyrick): Clarke, 1958: 572.

*Plalypeplus ptarmicopa* (Meyrick): Clarke, 1958: 575.

*Dudua charadraea* (Meyrick): Diakonoff, 1973: 427.

**Material examined.** Taiwan: 5 ♂♂, Nantow Co. Lushan, ca. 30 km E. Wushe, 1000 m, 27-31-V-1980, D. R. Davis; 1 ♂, Nantou Hsien, 23-VIII-1973, Y. Shibata; 1 ♂, Taipei Co. Chihsingshan, ca. 10 air km N Taipei, 700 m, 28-VII-1980, D. R. Davis; 1 ♂, Nantou Hsien, Lushan spa, 1200 m, 3-5-VIII-1985, A. Kawabe; 1 ♂, Rozan spa, Formosa, 27-VIII-1969, M. Nisikawa; 1 ♂, Hualien Hsien, Hungyeh spa, 200 m, 29-30-III-1984, A. Kawabe; 1 ♂, 1 ♀, Sozan, 12-VII-1933, S. Issiki; 1 ♀, Taihoku, 8-V-1935, S. Issiki; 1 ♂, Taihoku, 10-IX-1935, S. Issiki (Sun-243 ♂, USNM24098, UFCC); 1 ♀, Kansirei, 19-X-1934, S. Issiki (Sun-244 ♀, USNM24107, UFCC); 1 ♀, Taiwan, S. Issiki; 1 ♂, Hualian Co., Xiulin, Tianxiang, 6-IX-1995, Y. F. Hsu; 1 ♂, Hualien Hsien, Wenshan spa, 580 m, 4-5-VIII-1983, A. Kawabe; 1 ♂, Taiwan, Kansirei, 20-X-1934, S. Issiki; 1 ♂, Taiwan, Taihoku, 22-X-1935, S. Issiki; 1 ♀, Taiwan, Raisya, 21-XI-1934, S. Issiki [USNM]. 

1 ♂, Nantou Co. Lienhuachih For. Sta. Puli, 700 m, 4-VII-1996, J. Heppner and H. Wang (TORT12031 ♂, FL242); 1 ♂, Taitung Co. Bishan Hot Spgs., 720 m, 26-29-X-1984, J. Heppner and H. Wang (Gen. Sl. Bae UFL-048 ♂); 1 ♂, Taipei Co., Maiyueyuan, 900 m, 16-17-V-1989, J. Heppner and H. Wang (Gen. Sl. Bae UFL-013 ♂) [FSCA]. 

Hainan: Mt. Limu: 5 ♂♂, Forest Park (19.17° N, 109.73° E), 240~640 m, 3-V-2014~20-VII-2016, Peixin Cong (BX15358, BX15150, BX15748). Mt. Bawang: 3 ♂♂, Nancha River (19.22° N, 109.43° E), 600 m, 9-VI-2007, Weichun Zhang and Weichun Li (BX15755). Mt. Yingge: 4 ♂♂, Hongkan (19.08° N, 109.50° E), 508~540 m, 16-V-2015~17-III-2016, Peixin Cong (BX15745, BX15397). Baisha County: 2 ♂♂, Yaxing Village, Nankai Town (19.02° N, 109.40° E), 321 m, 20-VI-2015, Peixin Cong, Wei Guan, and Sha Hu (BX15144). Wuzhishan City: 2 ♂♂, Shuiman Town (18.88° N, 109.67° E), 690~766 m, 27-II~5-XI-2016, Qingyun Wang et al.; 1 ♂, Nature Reserves (18.90° N, 109.67° E), 738 m, 2-III-2016, Qingyun Wang et al. Hunan: 2 ♂♂, 1 ♀, Yantang Village, Weishan Town, Xinhua County, 6-VIII-2004, Yunli Xiao; 2 ♂♂, Maqiao Village, Baituo Town, Xiangtan City, 30-VII-2004, Yunli Xiao; 1 ♂, 1 ♀, Cangxi Town, Xinhua County, 8~9-VIII-2000, Yunli Xiao; 1 ♂, Heping Village, Chengguan Town, Taojiang County, 26-VII-2004, Yunli Xiao. Sichuan: 1 ♀, Wolong, 1900 m, 9-VIII-2004, Yingdang Ren. Taiwan: 4 ♂♂, Mt. Fu, Yilan County, 420 m, 31-VII-2006, Houhun Li and Xicui Du (YHL07035) [NKU]. 

**Distribution.** China (Hainan, Hunan, Sichuan, Taiwan), Indonesia, Japan, Micronesia, Sri Lanka, and Thailand.

#### 3.10.3. *Dudua crossotoma* (Meyrick, 1931)

*Argyroploce crossotoma* Meyrick, 1931: 129. TL: Bismark Archipelago, Bismarck Islands (New Hanover) [Papua New Guinea]. Lectotype: male, BMNH. 

*Dudua crossotoma* (Meyrick): Brown, 2005: 262.

**Material examined.** Taiwan: 1 ♂, Taiwan “B” (Sun-245 ♂, USNM17830, RL Brown) [USNM].

**Distribution.** China (Taiwan) and Papua New Guinea.

**Remarks.** This species is recorded for the first time in China.

#### 3.10.4. *Dudua dissectiformis* Yu et Li, 2005

*Dudua dissectiformis* Yu et Li, 2005: 278. TL: China, Zhejiang, Mt. Tianmu. Holotype: male, NKU. 

**Material examined.** Taiwan: 1 ♀, Nantow Co. Lushan, ca. 30 km E. Wushe, 1000 m, 27-31-V-1980, D. R. Davis (USNM144945 ♀, YH Sun); 1 ♂, Kao Hsiung Co. 1-2 km W Meishan, 870 m, 29-VI-2-VII-1980, D. R. Davis; 1 ♂, Nantou Hsien, Lushan spa, 1200 m, 27-29-VII-1983, A. Kawabe; 2 ♂♂, Nantou Hsien, Nanshanchi, 25-26-VII-1983, A. Kawabe; 1 ♂, Nantou Hsien, Yushih, 1700 m, 3-4-VIII-1987, A. Kawabe; 1 ♂, Nantou Hsien, Lushan spa, 1200 m, 9-10-VIII-1987, A. Kawabe (Sun-255 ♂, USNM144944, YH Sun); 1 ♂, Nantou Hsien, Lushan spa, 1200 m, 7-8-VI-1988, K. Fujisawa; 1 ♂, Kansirei, 25-III-35, S. Issiki; 1 ♂, 1 ♀, Taoyuan Co. Upper Palin, 12-VIII-1992, T.T.Lo [USNM]. 

1 ♀, Nantou Co. Lo Farm, 8 km E. Puli, 750 m, 10-11-VII-2005, J. Heppner; 1 ♂, Ilan Co. 5 km N. Nanao, 100 m, 11-13-X-1984, J. Heppner and H. Wang; 1 ♀, Hualien Co. 7 km. E. Kuangfu, 300 m, 26-VIII-1983, J. Heppner; 1 ♂, Kaohsiung Co. Liukuei, Shanpin For., 750 m, 16-23-III-1990, J. Heppner and H. Wang (TORT11846 ♂, FL059); 1 ♀, Taichung Co. Chingshan, 1100 m, 8-11-V-1989, J. Heppner and H. Wang (TORT12174 ♀, Gen. Sl. Bae UF-027) [FSCA].

**Distribution.** China (Anhui, Fujian, Guangxi, Guizhou, Hunan, Taiwan, Zhejiang).

**Remarks.** This species is recorded for the first time in Taiwan.

#### 3.10.5. *Dudua hemigrapta* (Meyrick, 1931)

*Argyroploce hemigrapta* Meyrick, 1931: 133. TL: Taiwan, Formosa [Taiwan] (Taihoku [Taipei]). Holotype: female, BMNH. 

*Olethreutes hemigrapta* (Meyrick): Clarke, 1958: 516.

*Dudua hemigrapta* (Meyrick): Diakonoff, 1973: 507.

**Material examined.** Taiwan: 1 ♀, Chiai Hsien, 1400 m, 11-12-VIII-1985, A. Kawabe; 1 ♀, Nantou Hsien, Lushan spa, 1200 m, 27-29-VII-1983, A. Kawabe (Sun-246 ♀, USNM122542, A. Kawabe); 1 ♂, Hualien Hsien, Losao, 1110 m, 2-IV-1984, A. Kawabe; ♂, Hualien Hsien, Hungyeh spa, 200 m, 29-30-III-1984, A. Kawabe (Sun-247 ♂, USNM122544, A. Kawabe); ♂, Nantou Hsien, Lushan spa, 1200 m, 27-29-VII-1983, A. Kawabe (Sun-248 ♂, USNM122545, A. Kawabe); 3 ♂♂, 1 ♀, Orchid Isl. 4 km SW Hungt’ou narrow valley, 5-10 m, 16-20-VII-1980, D. R. Davis; 2 ♂♂, Taipei Co. Chihsingshan, ca. 10 air km N Taipei, 700 m, 28-VII-1980, D. R. Davis; 1 ♂, TaiNanCo. 2-3 km S Kwantzuling, 26-28-VI-1980, D. R. Davis; 1 ♂, Kao Hsiung Co. 1-2 km W Meishan, 870 m, 29-VI-2-VII-1980, D. R. Davis; 1 ♀, Raisya, 25-XI-1934, S. Issiki [USNM]. 

4 ♂♂, 1 ♀, Hualien Co. Fuyan, 400 m, 7-III-1990, J. Heppner and H. Wang (TORT12066 ♂, FL177); 1 ♂, 1 ♀, Ilan Co. Fushan For. Sta., 650 m, 4-11-IV-1990, J. Heppner; 1 ♂, Ilan Co. Fushan For. Sta, 650 m, 20-24-VII-1996, J. Heppner; 1 ♀, Taoyuan Co. Upper Palin, 1750 m, 12-VIII-1992, T.T.Lo (TORT12067 ♀, FL178); 1 ♀, Ilan Co. Chilan Nsy., Tuchan, 480 m, 15-17-X-1984, J. Heppner and H. Wang; 1 ♀, Nantou Co. Lienhuachih For. Sta. 12 km. S. Puli, 700 m, 9-XI-1984, J. Heppner and H. Wang; 3 ♂♂, Ilan Co. Mingchyr Rec. Area, 1250 m, 9-10-VII-1996, J. Heppner and H. Wang (TORT12068 ♂); 1 ♀, Ilan Co. Mingchyr Rec. Area, 1250 m, 9-10-VII-1996, J. Heppner and H. Wang (TORT12061 ♀); 1 ♂, 1 ♀, Taitung Co. Bishan Hot Spgs., 720 m, 26-29-X-1984, J. Heppner and H. Wang; 1 ♂, Kaohsiung Co. Lukuei For. Sta., 750 m, 29-IV/3-V-1989, J. Heppner and H. Wang; 1 ♂, Kaohsiung Co. Tienchih, 2210 m, 24-X-1984, J. Heppner and H. Wang; 5 ♂♂, Nantou Co. 5 km N. Chitou, 500 m, 29-VI-1985, J. Heppner and H. Wang; 4 ♂♂, 1 ♀, Taoyuan Co. Upper Palin, 1500 m, 7-9-VII-1985, J. Heppner and H. Wang; 3 ♂♂, 2 ♀♀, Ilan Co. Chilan Nsy., Tuchan, 480 m, 15-17-X-1984, J. Heppner and H. Wang; 4 ♂♂, 1 ♀, Kaohsiung Co. Dona, 16 km NE. Maolin, 500 m, 3-XI-1984, J. Heppner and H. Wang; 1 ♂, 1 ♀, Taipei Co. Wulai, 200 m, 17-19-VI-1985, J. Heppner and H. Wang; 2 ♀♀, Ilan Co. 5 km N. Nanao, 100 m, 11-13-X-1984, J. Heppner and H. Wang; 1 ♂, Nantou Co. Tungpu, 1150 m, 1-VII-1985, J. Heppner and H. Wang; 1 ♀, Hualien Co. 7 km. E. Kuangfu, 300 m, 26-VIII-1983, J. Heppner; 1 ♂, Ilan Co. Tuchan, 480 m, 1-2-VII-1982, J. Heppner; 1 ♂, Hualien Co. Kuyuan, Taroko Gorge, 750 m, 6-7-VII-2005, J. Heppner; 1 ♂, Tainan Co. Kuantzuling, 250 m, 21-IV-1989, J. Heppner and H. Wang; 1 ♀, Kaohsiung Co. Lukuei For. Sta., 750 m, 29-IV/3-V-1989, J. Heppner and H. Wang; 1 ♂, Kaohsiung Co. Lukuei For. Sta., 750 m, 29-IV/3-V-1989, J. Heppner and H. Wang (TORT12181 ♂, Gen. Sl. Bae UF-028) [FSCA].

Guangdong: 3 ♂♂, 2 ♀♀, Mt. He, 6-XI~9-IX -2002, Guilin Liu and Binglan Zhang; 2 ♂♂, 4 ♀♀, Bubang Village, Mengla County, 650 m, 22~25-VIII-2005, Yingdang Ren. Hainan: Mt. Limu: 1 ♀, Nature Reserves (19.17° N, 109.74° E), 640 m, 1-V-2014, Tengteng Liu, Wei Guan, and Xuemei Hu. Wuzhishan City: 2 ♂♂, 2 ♀♀, Mt. Wuzhi (18.91° N, 109.67° E), 732 m, 4~5-VIII-2017, Xia Bai, Ping Liu, and Shuai Yu (BX15384 ♀, BX15385 ♂); 1 ♂, Chongtoumen, Shuiman Town (18.92° N, 109.68° E), 630 m, 1-VIII-2013, Haili Yu and Yao Fei (BX15744); 1 ♂, Lizudadian, Shuiman Town (18.88° N, 109.67° E), 766 m, 7-VII-2015, Qingyun Wang, Suran Li, and Mengting Chen (BX15399); 1 ♂, 3 ♀♀, Ecological Tea Sightseeing Garden, Shuiman Town (18.88° N, 109.67° E), 690 m, 5~6-XI-2016, Xia Bai, Shuonan Qian, and Wanding Qi (BX15752 ♀, BX15756 ♂); 1 ♂, Butterfly Ranch (18.87° N, 109.67° E), 680 m, 27-X-2016, Xia Bai, Shuonan Qian, and Wanding Qi; 3 ♂♂, 1 ♀, Nature Reserves (18.90° N, 109.67° E), 740 m, 14~15-IV-2009, Qing Jin and Bingbing Hu (BX15400 ♂); 1 ♀, Nature Reserves (18.91° N, 109.68° E), 710 m, 21-IV-2014, Tengteng Liu, Wei Guan, and Xuemei Hu (BX15353); 1 ♂, Nature Reserves (18.90° N, 109.67° E), 738 m, 29-X-2016, Xia Bai, Shuonan Qian, and Wanding Qi (BX15749). Yunnan: 1 ♂, Rare Botanical Garden, Ruili City, 1000 m, 6-VIII-2005, Yingdang Ren; 1 ♂, Hudie Spring, Dali City, 2030, 2-VIII-2005, Yingdang Ren. 

**Distribution.** China (Guangdong, Hainan, Taiwan, Yunnan) and Japan.

#### 3.10.6. *Dudua hesperialis* Walker, 1864

*Dudua hesperialis* Walker, 1864: 1000. TL: Borneo, Sarawak. Holotype: male, BMNH. 

**Distribution.** China (Taiwan), Indonesia, and Myanmar.

#### 3.10.7. *Dudua trunciformis* Liu et Yu, 2014

*Dudua trunciformis* Liu et Yu, 2014: 184. TL: China (Hainan). Holotype: male, NKU. 

**Material examined.** Taiwan: 1 ♀, Habon, Oct. A. 1926, S. Issiki [USNM]. 

1 ♀, Nantou Co., Lienhuachih For. Sta., 12 km. S. Puli, 700 m, 7-12-IX-1983, J. Heppner (TORT12057 ♀, Sun-FL221) [FSCA].

Hainan: Mt. Limu: 1 ♀, Forest Park (19.17° N, 109.73° E), 607 m, 25-X-2016, Xia Bai, Shuonan Qian, and Wanding Qi; Mt. Yingge: 1 ♂, Hongkan (19.08° N, 109.50° E), 540 m, 15-III-2016, Qingyun Wang, Suran Li, and Shengnan Zhao; 1 ♂, Yinggezui (19.05° N, 109.57° E), 623 m, 4-I-2018, Mujie Qi and Shuai Yu; 1 ♂, Yinggezui (19.05° N, 109.70° E), 599 m, 27-VII-2017, Xia Bai, Ping Liu, and Shuai Yu (BX15836). Wuzhishan City: 1 ♂, National Park (18.88° N, 109.67° E), 766 m, 8-I-2016, Kaijian Teng, Xia Bai, and Mengting Chen; Mt. Jianfeng: 1 ♂, 1 ♀, 18.73° N, 108.91° E, 940 m, 4~7-VI-2017, Zhiwei Zhang and Weichun Li (BX15750, BX15751); 1 ♂, Tianchi (18.73° N, 108.87° E), 787 m, 7-III-2016, Qingyun Wang, Suran Li, and Shengnan Zhao (BX15398) [NKU].

**Distribution.** China (Hainan, Taiwan).

### 3.11. Eudemis *Hübner, [1825] 1816*

*Eudemis* Hübner, [1825] 1816: 382. Type-species: *Tortrix porphyrana* Hübner, [1796−1799].

*Thirates* Treitschke, 1829: 233. Type-species: *Tortrix profundana* Denis et Schiffermüller, 1775.

#### 3.11.1. *Eudemis gyrotis* (Meyrick, 1909)

*Eudemis gyrotis* (Meyrick): Issiki, 1957: 69.

*Argyroploce gyrotis* Meyrick, 1909: 604. TL: India, Assam, Khasi Hills. Lectotype: female, BMNH. 

**Material examined.** Taiwan: 1 ♂, Tikusiko, 29-X-1935, S. Issiki (Sun-249 ♂, USNM145140, YH Sun); 1 ♀, Taihoku, 8-V-1935, S. Issiki; 1 ♀, Taihoku, 13-V-1935, S. Issiki [USNM].

Guizhou: 1 ♂, Xiaodanjiang, Rongjiang County, 680 m, 15-IX-2005, Jialiang Zhang [NKU].

**Host plant.** Myricaceae: *Myrica rubra* (Lour.).

**Distribution.** China (Aanhui, Fujian, Guangdong, Guangxi, Guizhou, Jiangxi, Sichuan, Taiwan), Japan, and Russia.

#### 3.11.2. *Eudemis porphyrana* (Hübner, [1796–1799])

*Tortrix porphyrana* Hübner, [1796–1799]: pl. 5, Figure 26. TL: Europe. Syntype(s): unknown.

*Olethreutes porphyrana* (Hübner): Hübner, 1822: 63.

*Eudemis porphyrana* (Hübner): Hübner, 1825: 382.

*Poecilochroma pomedaxana* (Hübner): Pierce & Metcalfe, 1915: 9. TL: England (Devonshire). Holotype: unknown, BMNH.

*Eudemis pomedaxana* (Hübner): Pierce & Metcalfe, 1922: 45.

**Material examined.** Taiwan: 1 ♀, Nantou Hsien, Tsuifeng, 2400 m, Dec. 29, 1989, A. Kawabe [USNM]. 

1 ♂, Kaohsiung Co. Lukuei For. Sta., 750 m, 29-IV/3-V-1989, J. Heppner and H. Wang; 8 ♂♂, 9 ♀♀, Nantou Co., Meifeng, 2100 m, 6-9-IX-1988, J. Heppner [FSCA].

**Host plants.** Rosaceae: *Padus avium* Mill., *Crataegus* spp., *Malus* spp., and *Pyrus* spp.; Fagaceae: *Quercus* spp.

**Distribution.** China (Fujian, Gansu, Guangdong, Guizhou, Heilongjiang, Henan, Hubei, Jiangxi, Jilin, Shaanxi, Sichuan, Taiwan, Zhejiang), Europe, Japan, and Russia.

**Remarks.** This species is recorded for the first time in Taiwan.

### 3.12. Eudemopsis *Falkovitsh, 1962*

*Eudemopsis* Falkovitsh, 1962: 190. Type-species: *Penthina purpurissatana* Kennel, 1901.

#### 3.12.1. *Eudemopsis brevis* Liu et Bai, 1982

*Eudemopsis brevis* Liu et Bai, 1982: 47. TL: China, Sichuan Province, Guanxian Co., Qingcheng Mt. Holotype: male, IZAS. 

**Material examined.** Taiwan: 6 ♂♂, Nantou Hsien, Lushan spa, 1200 m, 3-5-VIII-1985, A. Kawabe (Sun-250 ♂); 1 ♂, Taipei Co. Chihsingshan, ca. 10 air km N Taipei, 700 m, 28-VII-1980, D. R. Davis; 1 ♂, Taiwan, Rantaisan, 11-III-26, S. Issiki, no abdomen [USNM]. 

1 ♀, Ilan Co. Mingchyr Rec. Area, 1250 m, 9-10-VII-1996, J. Heppner and H. Wang (TORT12009 ♀, FL248); 1 ♂, Ilan Co. Fushan Botanical Sta., 650 m, 20-24-VII-1996, J. Heppner; 1 ♂, Taoyuan Co. Upper Palin, 1350 m, 4-5-VI-2005, J. Heppner (TORT12123 ♂, FL247); 1 ♂, Hsinchu Co. Litonshan, 1450-1914 m, 13-VII-2005, J. Heppner; 1 ♂, Kaohsiung Co. Liukuei, Shanpin For., 750 m, 16-23-III-1990, J. Heppner and H. Wang; 6 ♂♂, Taipei Co. Wulai, 200 m, 17-19-VI-1985, J. Heppner and H. Wang (Gen. Sl. Bae UFL-104 ♂) [FSCA].

Hainan: 1 ♂, Mt. Yingge (19.08° N, 109.50° E), 620 m, 21-VI-2010, Bingbing Hu and Jing Zhang (BX15515) [NKU].

**Distribution.** China (Hainan, Sichuan, Taiwan).

### 3.13. Hedya *Hübner, [1825] 1816*

*Hedya* Hübner, [1825] 1816: 380. Type-species: *Phalaena (Tortrix) salicella* Linnaeus, 1758.

*Episagma* Hübner, [1825] 1816: 383. Type-species: *Phalaena (Tortrix) schreberiana* Linnaeus, 1761. 

*Pendina* Treitschke, 1829: 227. Type-species: *Phalaena (Tortrix) salicella* Linnaeus, 1758.

*Penthina* Treitschke, 1830: 21 [unjustified emendation *Pendina*]. 

#### 3.13.1. *Hedya anaplecta* (Meyrick, 1909)

*Argyroploce anaplecta* Meyrick, 1909: 598. TL: Sri Lanka, Ceylon [Sri Lanka] (Maskeliya). Lectotype: male, BMNH.

*Olethreutes anaplecta* (Meyrick) Clarke, 1958: 483.

*Hedya anaplecta* (Meyrick): Heppner, 2012: 19.

**Material examined.** Taiwan: 1 ♂, Taoyuan Co., Upper Balin, 1750 m, 12-VIII-1992, T. T. Lo (Gen. Sl. Bae UFL-069 ♂); 1 ♀, Taiwan, Tyokakurai, 25-Mar-26, S. Issiki (Sun-336 ♀, USNM145141, YH Sun) [USNM].

Hainan: 1 ♂, National Park, Mt. Limu (19.17° N, 109.73° E), 607 m, 15-V-2015, Peixin Cong, Wei Guan, and Sha Hu (BX15485) [NKU].

**Distribution.** China (Hainan, Taiwan) and Sri Lanka.

#### 3.13.2. *Hedya auricristana* (Walsingham, 1900)

*Argyroploce auricristana* Walsingham, 1900: 237. TL: Japan. Syntypes: male and female, BMNH. 

*Olethreutes auricristana* (Walsingham): Inoue, 1954: 104, no. 579.

*Aphania auricristana* (Walsingham): Issiki, 1957: 73.

*Aphanina auricristana* (Walsingham): Inoue, 1959: 263.

*Hedya auricristana* (Walsingham): Diakonoff, 1973: 443.

**Material examined.** Taiwan: 1 ♂, Nantou Co., Tayuling, 2570 m, 10-14-VI-1982, J. Heppner (TORT12159 ♂, Gen. Sl. Bae UFL-046) [FSCA].

**Host plants.** Caprifoliaceae: *Lonicera* spp.

**Distribution.** China (Guizhou, Henan, Taiwan, Zhejiang) and Japan.

**Remarks.** This species is recorded for the first time in Taiwan.

#### 3.13.3. *Hedya iophaea* (Meyrick, 1912)

*Argyroploce iophaea* Meyrick, 1912: 873. TL: Sri Lanka, Ceylon [Sri Lanka] (Maskeliya). Lectotype: male, BMNH.

*Hedya iophaea* (Meyrick): Diakonoff, 1973: 437.

**Material examined.** Taiwan: 1 ♂, Hualien Hsien, Wenshan spa, 580 m, 4-5-VIII-1983, A. Kawabe (Sun-251 ♂, USNM145142, YH Sun); 1 ♀, Nantou Hsien, Lushan spa, 1200 m, 3-5-VIII-1985, A. Kawabe (Sun-252 ♀, USNM145143, YH Sun); 1 ♂, Sozan, 5-VII-1933, S. Issiki; 1 ♂, Kansirei, 19-X-1934, S. Issiki [USNM].

Guangdong: 1 ♂, Dawuling Natural Reserves, Xinyi City, 1000 m, 8-VIII-2003, Dandan Zhang. Hainan: Mt. Limu: 1 ♂, Forest Park (19.17° N, 109.73° E), 610 m, 4-V-2017, Xiaofei Yang; 1 ♀, Forest Park (19.17° N, 109.73° E), 607 m, 24-X-2016, Xia Bai, Shuonan Qian, and Wanding Qi (BX16492). Mt. Baiwang: 2 ♂♂, River Nancha (19.22° N, 109.43° E), 600 m, 10-VI-2007, Zhiwei Zhang and Weichun Li (BX15342). Mt. Yingge: 4 ♂♂, Mt. Yingge (19.08° N, 109.50° E), 620 m, 3~28-IV-2010, Bingbing Hu and Jing Zhang; 1 ♀, Yinggezui (19.05° N, 109.70° E), 700 m, 13-V-2017, Xiaofei Yang. Baisha County: 1 ♀, Hongxin Village, Yuanmen County (19.07° N, 109.52° E), 445 m, 22-III-2016, Qingyun Wang, Suran Li, and Shengnan Zhao. Wanning City: 1 ♂, Xinzhong Ranch (18.86° N, 110.20° E), 100 m, 3-VIII-2008, Bingbing Hu and Li Zhang. Wuzhishan City: 1 ♀, Shuiman County (18.88° N, 109.67° E), 31-V-2010, Bingbing Hu and Jing Zhang; 1 ♂, Shuiman County (18.86° N, 109.67° E), 700 m, 18-IV-2013, Yinghui Sun, Wei Guan, and Tengteng Liu; 1 ♂, Shuiman County (18.88° N, 109.67° E), 640 m, 28-X-2016, Xia Bai, Shuonan Qian, and Wanding Qi; 1 ♂, Wuzhishan Natural Reserves (18.90° N, 109.67° E), 740 m, 14-IV-2009, Qing Jin and Bingbing Hu. Mt. Jianfeng: 1 ♀, Natural Reserves (18.74° N, 108.84° E), 1050 m, 27-IV-2014, Tengteng Liu, Wei Guan, and Xuemei Hu; 1 ♀, Natural Reserves (18.74° N, 108.87° E), 770 m, 29-V-2015, Peixin Cong, Wei Guan, and Sha Hu. Mt. Diaoluo: 1 ♂, Natural Reserves (18.73° N, 109.87° E), 980 m, 24-IV-2014, Tengteng Liu, Wei Guan, and Xuemei Hu [NKU].

**Distribution.** China (Guangdong, Hainan, Taiwan), Russia, and Sri Lanka.

#### 3.13.4. *Hedya sunmoonlakensis* Kawabe, 1993

*Hedya sunmoonlakensis* Kawabe, 1993: 230. TL: Taiwan, Nantou Hsien, Sunmoonlake. Holotype: male, USNM. 

**Material examined.** Taiwan: holotype: ♂, Nantow Co. Sunmoon Lake, 760 m, 20-25-VI-1980, D. R. Davis (USNM92898 ♂, A. Kawabe) [USNM]. 

1 ♂, Henglong [in Chinese], 11-VII-1983, B.S. Chang (Sun-253 ♂, USNM92899, A. Kawabe); 1 ♂, Nantou Co. Lienhuachih For. Sta. 12 km. S. Puli, 700 m, 9-XI-1984, J. Heppner and H. Wang [FSCA].

Anhui: 1 ♂, Tangkou Town, Huangshan City, 5-VIII-2004, Jiasheng Xu and Jialiang Zhang. Fujian: 1 ♂, Gutian Town, Shanghang County, 600 m, 2-VI-2004, Haili Yu. Henan: 3 ♂♂, Huanglian Village, Jiyuan City, 700 m, 6~7-VI-2000, Haili Yu; 4 ♂♂, 1 ♀, Mt. Wangwu, Jiyuan City, 700 m, 4~5-VI-2000, Haili Yu; 3 ♂♂, Xiaguan Town, Neixiang Couty, 650 m, 12-VII-1998, Houhun Li; 3 ♂♂, Huangshian, Xixia County, 890 m, 16~19-VII-1998, Houhun Li; 2 ♂♂, 4 ♀♀, Shuiliandong, Tongbai County, 300 m, 24-VI~11-IX-2000, Haili Yu, Houhun Li, and Karsholt; 1 ♀, Mt. Song, Dengfeng City, 800 m, 9-VI-2000, Haili Yu. Hunan: 1 ♀, Zhangjiajie, 650 m, 8-VIII-2001, Houhun Li and Xinpu Wang. Shaanxi: 4 ♂♂, 2 ♀♀, Yangling City, 17~20-VI-1985, Houhun Li; 5 ♂♂, 3 ♀♀, Zhao Village, Chengcheng County, 1000 m, 9~11-VIII-1993, Houhun Li; 2 ♂, Wuan Village, Zhaozhuang Town, Chengcheng County, 1000 m, 7-VII~1-VIII-1986, Houhun Li; 1 ♂, Bin County, 4-VIII-1988; 1 ♂, Yangling City, 450 m, 9-VIII-1986, Houhun Li; 1 ♂, Bin County, 16-IX-1988, Houhun Li. Zhejiang: 1 ♂, Qingliang Feng, Linan City, 900 m, 8-VIII-2005, Yunli Xiao [NKU].

**Distribution.** China (Anhui, Fujian, Henan, Hunan, Shaanxi, Taiwan, Zhejiang).

#### 3.13.5. *Hedya vicinana* (Ragonot, 1894)

*Conchylis vicinana* Ragonot, 1894: 200. TL: Russia, Amur. Syntype(s): unknown, MNHN. 

*Hedya vicinana* (Ragonot): Liu et Bai, 1977: 73.

**Material examined.** Taiwan: 4 ♂♂, Nantow Co. Tayuling, 2500 m, 9-18-VI-1980, D. R. Davis (Sun-254 ♂) [USNM].

Gansu: 9 ♂♂, 9 ♀♀, Mt. Xinglong, Yuzhong County, 2120~2230 m, 30-VII~4-VIII-2001, Houhun Li. Hebei: 2 ♂♂, 8 ♀♀, Xiaowutai, Yu County, 1200 m, 22~23-VIII-2005, Yunli Xiao. Henan: 3 ♂♂, 6 ♀♀, Baotianman, Neixiang County, 1350 m, 13~15-VII-1998, Houhun Li; 1 ♂, Xiaguan, Neixiang County, 650 m, 12-VII-1998, Houhun Li; 1 ♂, Mt. Baiyun, Song County, 1580 m, 18-VII-2002, Xinpu Wang. Inner Mongolia: 9 ♂♂, 2 ♀♀, Mt. Manhan, Liangcheng County, 1300~1450 m, 9~10-VIII-2002, Dandan Zhang, Zhiqiang Li. Jilin: 1 ♂, 7 ♀♀, Erdao Town, 1010 m, 4~5-VIII-2004, Aihuan Zhang; 1 ♀, Mt. Changbai, 960 m, 3-VIII-2004, Aihuan Zhang. Qinghai: 1 ♀, Mengda, Xunhua County, 2240 m, 15-VII-1995, Houhun Li and Shuxia Wang. Shaanxi: 1 ♂, Huoditang, Ningshan County, 15-VI-1987, Houhun Li; 1 ♂, Xinjia Forest Farm, Feng Couty, 1600 m, 10-VII-1988, Houhun Li. Sichuan: 2 ♂♂, Kangding City, 2400 m, 8-VII-2001, Houhun Li and Xinpu Wang; 13 ♂♂, 1 ♀, Jiuzhaigou, 2300~2700 m, 13~16-VIII-2002, Shulian Hao; 1 ♂, 1 ♀, Wolong, 1900 m, 7-VIII-2004, Yingdang Ren; 3 ♂♂, Zhongdian, 3150 m, 14-VII-2001, Houhun Li and Xinpu Wang [NKU].

**Host plants.** Salicaceae: *Salix* spp., *S. caprea* Linn., *S. koreana* Anderss., *S. sachalinensis* F. Schmidt, and *S. zerophila* Floder.

**Distribution.** China (Gansu, Hebei, Heilongjiang, Henan, Inner mongolia, Jilin, Qinghai, Shaanxi, Sichuan, Taiwan, Yunnan), Japan, and Russia.

### 3.14. Hystrichoscelus *Walsingham, 1900*

*Hystrichoscelus* Walsingham, 1900: 335. Type-species: “*Hystrichoscelus spathanum* Walsingham, 1900”.

#### 3.14.1. *Hystrichoscelus spathanum* Walsingham, 1900

*Hystrichoscelus spathanum* Walsingham, 1900: 336. TL: Japan. Syntypes: male and female, BMNH. 

**Distribution.** China (Taiwan), Japan, and Russia.

### 3.15. Lobesia *Guenée, 1845*

*Lobesia* Guenée, 1845: 297. Type-species: *Asthenia reliquana* Hübner, [1825] 1816.

*Analdes* Turner, 1916: 533. Type-species: *Analdes hypolepta* Turner, 1916. 

*Byrsoptera* Lower, 1901: 77. Type-species: *Byrsoptera xylistis* Lower, 1901 [preoccupied]. 

*Harmosma* Diakonoff, 1963: 354. Type-species: *Polychrosis harmonia* Meyrick, 1908. 

*Lomaschiza* Lower, 1901: 68. Type-species: *Lomaschiza physophora* Lower 1901. 

*Lomaschizodes* Diakonoff, 1954: 15. Type-species: *Grapholitha extrusana* Walker, 1863. 

*Neolobesia* Bae et Komai, 1991: 136. Type-species: *Lobesia coccophaga* Falkovitsh, 1970 [subgenus of *Lobesia*].

*Pirireisia* Kocak, 1981: 114 [replacement name for *Byrsoptera*]. 

*Polychrosis* Ragonot, 1894: 209. Type-species: *Tortrix botrana* Denis et Schiffermüller, 1775. 

*Steriophotis* Bradley, 1961: 123 [misspelling of *Steriphotis*].

*Steriphotis* Meyrick, 1911: 259. Type-species: *Steriphotis peltophora* Meyrick, 1911. 

#### 3.15.1. *Lobesia aeolopa* Meyrick, 1907

*Lobesia aeolopa* Meyrick, 1907: 976. TL: Sri Lanka, Ceylon [Sri Lanka] (Maskeliya). Lectotype: male, BMNH. 

*Lobesia dryopelta* Meyrick, 1932: 225. TL: Indonesia. Java, teak forest. Lectotype: male, BMNH.

*Lobesia eustales* Bradley, 1956: 146. TL: Australia. Lord Howe Island, Mt. Lidgbird. Holotype: male, BMNH. 

**Material examined.** Taiwan: 1 ♂, Miaoli Hsien, Hernglong Lodge, 550 m. nr Taian spa, 8-VIII-1983, A. Kawabe; 2 ♂♂, Miaoli Hsien, Hernglong Lodge, 550 m. nr Taian spa, 8-VIII-1983, A. Kawabe; 3 ♂♂, Huali Hsien, Hungyeh spa, 29-30-III-1984, A. Kawabe (Sun-256 ♂); 1 ♂, 1 ♀, Nantou Hsien, Nanshanchi, 25-26-VII-1983, A. Kawabe; 1 ♂, Hualien Hsien, Tienshiang, 31-III-1984, A. Kawabe; 1 ♂, Taoyuan Hsien, Palin, 1000 m, 31-VII-2-VIII-1985, A. Kawabe; 2 ♂♂, (Wurai) Taipei Hsien, Formosa, 30-VII-1974, Y. Kishida; 1 ♂, Hualien Hsien, Wenshan spa, 580 m, 4-5-VIII-1983, A. Kawabe; 1 ♂, Wu Lai, Taipei, Hsien, Formosa, 30-VII-1974, H. Nakajima; 1 ♂, Taihoku, 19-VI-1934, S. Issiki; 1 ♂, Taihoku, 4-XI-1933, S. Issiki [USNM].

Anhui: 2 ♂♂, Wenquan Town, Yuexi County, 18~22-VIII-1995, Xiangfu Hu; 3 ♂♂, 1 ♀, Mozitan, Huoshan County, 12-VIII-2004, Jiasheng Xu and Jialiang Zhang; 1 ♂, Ke Village, Mt. Jiuhua, 8-VIII-2004, Jiasheng Xu and Jialiang Zhang; 5 ♂♂, 1 ♀, Tanqiao Town, Huangshan City, 6~7-VIII-2004, Jiasheng Xu and Jialiang Zhang; 5 ♂♂, Tangkou Town, Huangshan City, 4~5-VIII-2004, Jiasheng Xu and Jialiang Zhang; 1 ♂, Mt. Tianzhu, 11-VIII-2004, Jiasheng Xu and Jialiang Zhang. Fujian: 7 ♂♂, 2 ♀♀, Mt. Wuyi, 600~740 m, 19~27-V-2004, Haili Yu; 1 ♂, 1 ♀, Gutian Town, Shanghang County, 600 m, 2-VI-2004, Haili Yu; 1 ♂, Mt. Qingyun, Yongtai County, 550 m, 18-IX-2002, Xinpu Wang; 1 ♀, weather station, Jianning County, 350 m, 25-IX-2002, Xinpu Wang; 1 ♀, Maodi, Nanping City, 850 m, 23-IX-2002, Xinpu Wang. Guangdong: 7 ♂♂, 4 ♀♀, Mt. He, 10-X~18-XII-2002, Guilin Liu and Binglan Zhang; 2 ♂♂, Mt. Dadong, Lianzhou City, 650 m, 21-VI-2004, Dandan Zhang; 1 ♀, Mt. Dawu, Xinyi City, 1000 m, 8-VIII-2003, Dandan Zhang. Guangxi: 1 ♂, 2 ♀♀, Pinglang Town, Heng County, 7~18-VII-2002, Yanli Du; 1 ♂, 1 ♀, Rongshui County, 579~650 m, 13~19-VII-2004, Jiasheng Xu; 1 ♂, Mt. Maoer, 1-VII-2001, unknown collector; 1 ♀, Leye County, 1160 m, 26-VII-2004, Jiasheng Xu. Guizhou: 5 ♂♂, 1 ♀, River Dasha, Daozhen County, 1350 m, 24-VIII-2004, Yunli Xiao; 3 ♂♂, 2 ♀♀, Xiannvdong, Daozhen County, 600 m, 17-VIII-2004, Yunli Xiao; 1 ♂, Guo Village, Daozhen County, 1300 m, 20-VIII-2004, Yunli Xiao; 1 ♂, River Heiwan, Jiangkou County, 600 m, 27-VII-2001, Houhun Li and Xinpu Wang; 5 ♀♀, River Xiaodan, Rongjiang County, 680 m, 15~16-IX-2005, Jialiang Zhang; 1 ♀, Dongtang, Maolan Town, 26-V-1998, Qirong Liao. Hainan: Lingao County: 4 ♂♂, 1 ♀, Duowenling (19.80° N, 109.77° E), 120-207 m, 1-V-2009~20-VIII-2017, Bingbing Hu et al.; 40 ♂♂, 10 ♀♀, Gaoshanling (19.93° N, 109.64° E), 171 m, 18-VIII-2017~3-VI-2018, Baixia et al. Qionghai City: 1 ♂, Baishiling (19.17° N, 110.40° E), 58 m, 18-VI-2018, Ping Liu, Xia Bai, and Shuai Yu (BX16438); Mt. Limu: 5 ♂♂, 3 ♀, Forest Park (19.17° N, 109.73° E), 607 m, 15-V-2015~26-X-2016, Peixin Cong et al. (BX15126); 2 ♂♂, Nature Reserves (19.17° N, 109.73° E), 632 m, 3-VII-2015~5-I-2015, Qingyun Wang. Mt. Bawang: 2 ♂♂, (19.11° N, 109.09° E) 146 m, 12~15-VIII-2017, Ping Liu, Xia Bai, and Shuai Yu (BX16447); 3 ♂♂, Work Station (19.12° N, 109.09° E), 1000 m, 2009.IV.23, Bingbing Hu and Qing Jin; 3 ♂♂, 3 ♀♀, Forestry Administration (19.11° N, 109.09° E), 137 m, 22~24-IV-2009, Qing Jin and Bingbing Hu (BX15122, BX15798, BX15799); 3 ♂♂, Yaga II Hydropower Station (19.08° N, 109.10° E), 261 m, 19~21-VII-2015, Qingyun Wang, Suran Li, and Mengting Chen (BX15794); 5 ♂♂, 3 ♀♀, Nature Reserves (19.12° N, 109.08° E), 161 m, 7-VI-2015~19-I-2016, Peixin Cong et al. (BX15796); 1 ♀, Nancha River (19.22° N, 109.43° E), 600 m, 9-VI-2007, Zhiwei Zhang and Weichun Li; Dongfang City: 7 ♂♂, 1 ♀, Datian Nature Reserves (19.11° N, 108.79° E), 100 m, 26-IV~1-XII-2009, Qing Jin et al. (BX15800); 1 ♀, Datian Village (19.05° N, 108.84° E), 119 m, 2-I-2018, Mujie Qi and Shuai Yu (BX16450). Mt. Yingge: 35 ♂♂, 7 ♀♀, Ranger Station (19.08° N, 109.50° E), 508 m, 15~17-VI-2015, Peixin Cong, Wei Guan, and Sha Hu (BX15120, BX15124, BX15131). Baisha County: 10 ♂♂, 1 ♀, Hongxin Village, Yuanmen County (19.07° N, 109.52° E), 445 m, 1~3-VIII-2015, Qingyun Wang, Suran Li, and Mengting Chen (BX15776); 1 ♂, 2 ♀♀, Yaxing Village, Nankai County (19.02° N, 109.40° E), 321 m, 21-VI-2015, Peixin Cong, Wei Guan, and Sha Hu (BX15130). Wanning City: 1 ♀, Xinzhong Forest Form (18.86° N, 110.20° E), 110 m, 3-VIII-2018, Bingbing Hu and Li Zhang. Wuzhishan City: 1 ♀, Lizudadian, Shuiman County (18.88° N, 109.67° E), 766 m, 6-VII-2015, Qingyun Wang, Suran Li, and Mengting Chen (BX15793); 2 ♂♂, 2 ♀♀, Nature Reserves (18.88° N, 109.65° E), 742 m, 18~22-V-2015, Peixin Cong, Wei Guan, and Sha Hu (BX15766, BX15802). Mt. Diaoluo: 1 ♀, (18.67° N, 109.52° E) 84 m, 22-VIII-2017, Xia Bai, Ping Liu, and Shuai Yu (BX16448). Ledong County: 2 ♂♂, Jianfeng Town (18.70° N, 108.79° E), 40 m, 28-IV-2013, Yinghui Sun, Wei Guan, and Tengteng Liu (BX15792). Mt. Jianfeng: 1 ♀, (18.73° N, 108.91° E) 940 m, 6-VI-2007, Zhiwei Zhang and Weichun Li; 3 ♂♂, Tianchi (18.74° N, 108.84° E), 790~810 m, 30-III~1-IV-2008, Bingbing Hu and Haiyan Bai; 1 ♂, 2 ♀♀, Tianchi (18.74° N, 108.84° E), 21-IV-2010, Bingbing Hu and Jing Zhang; 2 ♂♂, Tianchi (18.73° N, 108.87° E), 787 m, 13~17-I-2016, Kaijian Teng, Xia Bai, and Mengting Chen (BX15788). Baoting County: 1 ♂♂, Pailiao Village (18.70° N, 109.67° E), 130 m, 25-IV-2013, Yinghui Sun, Wei Guan, and Tengteng Liu (BX15806). Henan: 12 ♂♂, 2 ♀♀, Shuiliandong (300 m), Tongbai County, 300 m, 11~14-IX-2000, Houhun Li et al. Hubei: 8 ♂♂, 3 ♀♀, Songbai Town, Shennongjia, 1200~1400 m, 17-VII-2003, Shulian Hao; 1 ♂, Badaling, Jingzhou City, 100 m, 26-VII-2003, Shulian Hao; 4 ♂♂, 2 ♀♀, Maobaqu, Lichuan City, 700 m, 28~30-VII-1999, Houhun Li. Hunan: 6 ♂♂, Zhangjiajie, 650 m, 7~11-VIII-2001, Houhun Li and Xinpu Wang; 27 ♂♂, 3 ♀♀, Xinhua County, 3~11-VIII-2004, Yunli Xiao; 5 ♂♂, 3 ♀♀, Xiangtan City, 29~30-VII-2004, Yunli Xiao; 2 ♂♂, 1 ♀, Taojiang County, 26~27-VII-2004, Yunli Xiao. Shaanxi: 1 ♂, Yueba Town, Foping County, 22-VII-1985, Houhun Li; 1 ♂, Ziyang County, 350 m, 21-V-1994, Jin Zhou; 1 ♂, Tieyupu Town, Danfeng County, 680 m, 27-V-1994, Jin Zhou; 4 ♂♂, Bifenggou, Wen County, 860 m, 9~13-VII-2005, Haili Yu. Sichuan: 2 ♂♂, Haichao, Lu County, 23-VII-1995, Yongxing Zeng; 1 ♂, Fengyongzhai, Baoxing County, 1600 m, 3-VIII-2004, Yingdang Ren; 1 ♂, 1 ♀, River Laba, Tianquan County, 1300 m, 29-VII-2004, Yingdang Ren; 1 ♂, Baoxing County, 1100 m, 5-VIII-2004, Yingdang Ren. Yunnan: 1 ♂, Qingshuihe Power Station, Yuanjiang County, 710 m, 27-IV-1995, Guangyun Yan; 1 ♂, Guanping Village, Mengyang Town, 1200 m, 18-VIII-2005, Yingdang Ren; 1 ♀, Menglun Botanical Garden, Xishuangbanna, 630 m, 20-IV-1995, Guangyun Yan; 1 ♂, Wenquan, Kunming City, 1900 m, 30-VIII-2005, Yingdang Ren. Zhejiang: 1 ♂, Mt. Tianmu, 10-VII-2000, Ping You; 8 ♂♂, 2 ♀♀, Mt. Tianmu, 350~1500 m, 15~20-VIII-1999, Houhun Li; 7 ♀♀, Wuyanling, Taishun County, 680~1050 m, 28-VII~3-VIII-2005, Yunli Xiao [NKU].

**Host plants.** Ulmaceae: *Ulmus parvifolia* Jacquin; Fabaceae: *Albizzia julibrissin* Durazzini; Rosaceae: *Eriobotrya japonica* (Thunb.) and *Prunus xyedoensis* Matsumura; Euphorbiaceae: *Glochidion hongkongense* Muell.-Arg. and *Ricinus communis* Linn.; Vitaceae: *Vitis* spp.; Actinidiaceae: *Actinidia chinensia* Planch. var. *hispida* C. F. Liang; Ebenaceae: *Diospyros kaki* Thunb.; Theaceae: *Thea sinensis* Linn.; Asteraceae: *Pluchea indica* Less.; Rutaceae: *Citrus* spp.; Sterculiaceae: *Melochia indica* Gray.

**Distribution.** China (Anhui, Fujian, Gansu, Guangdong, Guangxi, Guizhou, Hainan, Heilongjiang, Henan, Hubei, Hunan, Jiangxi, Shaanxi, Sichuan, Yunnan, Taiwan, Zhejiang), Africa, Japan, India, Indonesia, Korea, Madagascar, New Guinea, Solomon Islands, and Sri Lanka.

#### 3.15.2. *Lobesia ambigua* Diakonoff, 1954

*Lobesia (Lobesia) ambigua* Diakonoff, 1954: 40. TL: Indonesia, East Java, Tengger Mountains, Nongkodjadjar. Holotype: male, RMNH. 

*Lobesia ambigua*: Kawabe, 1992: 105.

**Material examined.** Taiwan: 2 ♂♂, Nantou Hsien, Wushe, 1150 m, 5-6-VI-1988, K. Fujisawa; 1 ♂, 1 ♀, Chiai Hsien, Fenchihu, 1400 m, 11-12-VIII-1985, A. Kawabe (Sun-257 ♂); 1 ♂, 1 ♀, Hualien Hsien, Wenshan spa, 580 m, 4-5-VIII-1983, A. Kawabe [USNM].

Fujian: 2 ♂♂, Xianfengling, Mt. Wuyi, 1000 m, 26-V-2004, Haili Yu. Gansu: 1 ♂, Bifenggou, Wen County, 860 m, 13-VII-2005, Haili Yu. Guizhou: 2 ♂♂, 6 ♀♀, Linjiang Village, Xishui County, 500 m, 25~28-IX-2000, Haili Yu; 1 ♂, 2 ♀♀, Suoluo, Chishui City, 240 m, 21~23-IX-2000, Haili Yu; 3 ♂♂, 2 ♀♀, River Dasha, Daozhen County, 1350~1370 m, 24~25-VIII-2004, Yunli Xiao; 1 ♂, Xiannvdong, Daozhen County, 600 m, 18-VIII-2004, Yunli Xiao; 3 ♂♂, 1 ♀, Guo Village, Daozhen County, 1300 m, 20~21-VIII-2004, Yunli Xiao; 1 ♂, Pingyuan Village, Daozhen County, 1300 m, 22-VIII-2004, Yunli Xiao. Hainan: Wuzhishan City: 1 ♂, Ecological Tea Garden (18.88° N, 109.67° E), Shuiman Town, 690 m, 6-XI-2016, Xia Bai, Shuonan Qian, and Wanding Qi (BX15767); 1 ♀, National Park (18.88° N, 109.67° E), 766 m, 8-I-2016, Kaijian Teng, Xia Bai, and Mengting Chen (BX15772); 2 ♂♂, Nature Reserves (18.90° N, 109.67° E), 738 m, 22~30-VII-2017, Xia Bai, Shuonan Qian, and Wanding Qi. Henan: 1 ♀, Mt. Gan, Shan County, 1100 m, 1-VI-2000, Haili Yu. Hubei: 1 ♀, Shayuan, Hefeng, 1260 m, 16-VII-1999, Houhun Li. Hunan: 6 ♂♂, 7 ♀♀, Nanping, Mt. Huping, Shimen County, 504 m, 4-V-2002, Haili Yu. Yunnan: 2 ♂♂, 1 ♀, Guanping Village, Mengyang Town, 1200 m, 20-VIII-2005, Yingdang Ren; 1 ♂, Bubang, Mengla Town, 650 m, 22-VIII-2005, Yingdang Ren. Zhejiang: 1 ♂, 1 ♀, Wuyanling, Taishun County, 1050 m, 30-VII-2005, Yunli Xiao; 8 ♂♂, 1 ♀, Qingliangfeng, Linan City, 900 m, 8~10-VIII-2005, Yunli Xiao [NKU].

**Host plants.** Moraceae: *Morus alba* Linn.; Papilionaceae: *Crotalaria* spp.

**Distribution.** China (Fujian, Gansu, Guizhou, Hainan, Henan, Hubei, Hunan, Jiangxi, Shandong, Yunnan, Taiwan, Zhejiang), Indonesia, Japan, and Thailand.

#### 3.15.3. *Lobesia atsushii* Bae, 1993

*Lobesia atsushii* Bae, 1993: 516. TL: Taiwan (Hualien Hsien, Mt. Hohuanshan). Holotype: male, USNM. 

**Material examined.** Taiwan: holotype: ♂, Hualien Hsien, Hohuanshan, 3100 m, 30-VII-1-VIII-1983, A. Kawabe (examined) [USNM].

**Distribution.** China (Taiwan). 

#### 3.15.4. *Lobesia cunninghamiacola* (Liu et Bai, 1977)

*Polychrosis cunninghamiacola* Liu et Bai, 1977: 217 TL: China, Jiangsu, Nanking. Holotype: female, IZAS. 

*Lobesia cunninghamiacola* (Liu et Bai): Kawabe, 1992: 105.

*Lobesia (Neodasyphora) cunninghamiacola*: Razowski, 1995: 297.

**Material examined.** Taiwan: 1 ♂, 1 ♀, Nantou Hsien, Wushe, 1150 m, 5-6-VI-1988, K. Fujisawa; 1 ♀, Nantou Hsien, Lushan spa, 1200 m, 7-8-VI-1988, K. Fujisawa; 1 ♂, Dongpu [in Chinese], 16-VI-1985, B.S. Chang; 1 ♂, Taiwan, S. Issiki; 1 ♂, Taiwan, Hori, 9-VI-1943, S. Issiki (Sun-258 ♂); 1 ♀, Taiwan, Baibara, 24-III-43, S. Issiki; 1 ♀, Taiwan, Baibara, 26-III-43, S. Issiki; 1 ♀, Miaoli Hsien, Hernglong Lodge, 550 m. nr Taian spa, 8-VIII-1983, A. Kawabe; 1 ♂, Taiwan, Higasinoko, 3-VI-43, S. Issiki [USNM]. 

1 ♂, Nantou Co. Lienhuachih For. Sta. 12 km. S. Puli, 700 m, 4-VII-1996, J. Heppner and H. Wang; 1 ♂, 1 ♀, Kaohsiung Co. Liukuei, Shanpin For., 750 m, 16-23-III-1990, J. Heppner and H. Wang [FSCA].

Anhui: 1 ♀, Tanqiao Town, Huangshang City, 6-VIII-2004, Jiasheng Xu and Jialiang Zhang. Fujian: 1 ♂, Gutian Town, Shanghang County, 600 m, 2-VII-2004, Haili Yu. Gansu: 1 ♂, Bifenggou, Wen County, 860 m, 14-VII-2005, Haili Yu. Guangxi: 3 ♂♂, Peixiu Village, Rongshui County, 579 m, 13-VII-2004, Jiasheng Xu; 1 ♂, Yachang Forest Farm, Leye County, 665 m, 24-VII-2004, Jiasheng Xu. Guizhou: 1 ♀, Heiwan, Mt. Fanjing, 530 m, 2-VI-2002, Xinpu Wang. Hainan: Lingao County: 1 ♀, Mt. Gaoshan (19.93° N, 109.64° E), 168 m, 2-VI-2018, Ping Liu, Xia Bai, and Shuai Yu. Mt. Bawang: 1 ♀, Work Station (19.12° N, 109.09° E), 1000 m, 6-IV-2008, Bingbing Hu and Haiyan Bai. Mt. Yingge: 1 ♀, Nankai Station (19.07° N, 109.40° E), 210 m, 16-VIII-2016, Xia Bai, Shuonan Qian, and Wanding Qi (BX15775). Mt. Jianfeng: 6 ♀♀, (18.75° N, 108.87°) 745 m~810 m, 11-VIII-2017~13-VI-2018, Xia Bai, Ping Liu, and Shuai Yu. Henan: 4 ♂♂, 3 ♀♀, Lingshansi, Mt. Luo, 350 m, 21~23-V-2000, Haili Yu; 1 ♂, Shuiliandong, Tongbai County, 300 m, 25-V-2000, Haili Yu. Hubei: 1 ♂, Maobaqu, Lichuan City, 700 m, 29-VII-1999, Houhun Li; 1 ♂, 1 ♀, Shayuan Village, Hefeng County, 1260 m, 15~16-VII-1999, Houhun Li; 4 ♂♂, 2 ♀♀, Badaling, Jingzhou City, 100 m, 26-VII-2003, Shulian Hao. Hunan: 1 ♂, Cangxi Town, Xinhua County, 8-VIII-2004, Yunli Xiao; 1 ♀, Zhangjiajie, 650 m, 8-VIII-2001, Houhun Li and Xinpu Wang [NKU].

**Host plant.** Taxodiaceae: *Cunninghamia lanceolata* (Lamb.).

**Distribution.** China (Anhui, Fujian, Gansu, Guangxi, Guizhou, Hainan, Henan, Hubei, Hunan, Jiangsu, Jiangxi, Taiwan).

#### 3.15.5. *Lobesia genialis* Meyrick, 1912

*Lobesia genialis* Meyrick, 1912: 869. TL: Ceylon [Sri Lanka] (Peradeniya). Holotype: male, BMNH.

*Lobesia (Lomaschiza) genialis*: Diakonoff, 1954: 24.

**Material examined.** Taiwan: 1 ♀, Taitung Hsien, Chihpen spa, 200 m, 28-III-1984, A. Kawabe (Sun-259 ♀) [USNM].

**Host plants.** Verbenaceae: *Lantana* spp.; Sapindaceae: *Dimocarpus longan* Lour.; Anacardiaceae: *Mongifera indica* Linn.; Clusiaceae: *Garcinia mangostana* Linn. 

**Distribution.** China (Taiwan, Yunnan), Sri Lanka, and Thailand.

#### 3.15.6. *Lobesia lithogonia* Diakonoff, 1954

*Lobesia lithogonia* Diakonoff, 1954: 49. TL: Indonesia (West Java, Buitenzorg). Holotype: male, RMNH.

**Material examined.** Taiwan: 2 ♂♂, Nantou Hsien, Nanshanchi, 25-26-VII-1983, A. Kawabe; 3 ♂♂, 1 ♀, Taitung Hsien, Chihpen spa, 200 m, 28-III-1984, A. Kawabe (Sun-260 ♂); 1 ♂, Nantou Hsien, Lushan spa, 1200 m, 3-5-VIII-1985, A. Kawabe; 1 ♀, Taiwan, Orchid Isl. 4 km SW Hungt’ou narrow valley, 5-10 m, 16-20-VII-1980, D. R. Davis; 2 ♀♀, Taiwan, Taidong Co., Lanyu, Langdao, 14-IV-2003, Y. F. Hsu [USNM].

Hainan: Danzhou City: 1 ♀, Songtao Reservoir (19.27° N, 109.66° E), 277 m, 28-XII-2017, Mujie Qi and Shuai Yu; 1 ♂, Fanjia Village (19.32° N, 109.69° E), 193 m, 27-XII-2017, Mujie Qi and Shuai Yu (BX16441); 3 ♂♂, 4 ♀, Hongkan Village (19.08° N, 109.50° E), 540 m, 24~27-VII-2015, Qingyun Wang, Suran Li, and Mengting Chen (BX15782); 1 ♂, 1 ♀, Hongkan Village (19.08° N, 109.50° E), 540 m, 22-I-2016, Kaijian Teng, Xia Bai, and Mengting Chen; 1 ♂, Nankai Station (19.07° N, 109.40° E), 210 m, 11~16-VIII-2016, Xia Bai, Shuonan Qian, and Wanding Qi (BX15805). Chengmai County: 1 ♀, Mt. Jigong (19.51 N, 110.08° E), 138 m, 23-VI-2018, Ping Liu, Xia Bai, and Shuai Yu (BX16449); 1 ♂, Mt. Wolong (19.46° N, 110.12° E), Tunchang County, 126 m, 21-VI-2018, Ping Liu, Xia Bai, and Shuai Yu (BX16443). Mt. Bawang: 4 ♂♂, 1 ♀, Yajia Hydropower Station (19.10° N, 109.11° E), 245 m, 7-V-2013, Yinghui Sun, Wei Guan, and Tengteng Liu (BX15797); Datian Nature Reserves (19.11° N, 108.79° E). Dongfang City: 1 ♀, 122 m, 1-I-2018, Mujie Qi and Shuai Yu; 2 ♂♂, 56 m, 6~7-VI-2018, Ping Liu, Xia Bai, and Shuai Yu (BX16440); Lemei Village (19.08° N, 108.84° E): 1 ♂, 81 m, 3-I-2018, Mujie Qi and Shuai Yu; 2 ♂♂, 2 ♀♀, 124 m, 8-VI-2018, Ping Liu, Xia Bai, and Shuai Yu (BX16439). Mt. Yingge (19.08° N, 109.50° E): 620 m: 2 ♂♂, 8~10-XII-2010, Zhaohui Du and Linlin Yang; 7 ♂♂, 1 ♀, 14-IV~6-V-2010, Bingbing Hu and Jing Zhang (BX15781); 1 ♂, Yinggezui (19.05° N, 109.57° E), 599 m, 27-VII-2017, Xia Bai, Ping Liu, and Shuai Yu (BX16452). Baisha County: Hongxin Village, Yuanmen Village (19.08° N, 109.52° E): 2 ♂♂, 430 m, 17-IV-2014, Tengteng Liu, Wei Guan, and Xuemei Hu; 4 ♂♂, 445 m, 1~3-VIII-2015, Qingyun Wang, Suran Li, and Mengting Chen (BX15777). Wanning City: 1 ♂, Jianling Primary School (18.89° N, 110.25° E), Beida Town, 110 m, 29-VII-2008, Bingbing Hu and Li Zhang. Wuzhishan City: 10 ♂♂, 6 ♀♀, Maoyang Town (18.94° N, 109.51° E), 225 m, 18~21-IV-2009, Qing Jin and Bingbing Hu (BX15761). Shuiman Town (18.88° N, 109.67° E): 20 ♂♂, 1 ♀, 640~650 m, 15~21-V-2007, Zhiwei Zhang and Weichun Li (BX15765); 4 ♂♂, 630 m, 16-IV-2009, Qing Jin and Bingbing Hu (BX15762); 3 ♂♂, 1 ♀, 700 m, 18~19-IV-2013, Yinghui Sun, Wei Guan, and Tengteng Liu (BX15123, BX15769); 1 ♂, 1 ♀, 766 m, 5~6-VII-2015, Qingyun Wang, Suran Li, and Mengting Chen (BX15801, BX15808); 2 ♀♀, 766 m, 28-X-2016, Xia Bai, Shuonan Qian, and Wanding Qi (BX15773). Mt. Wuzhi (18.87° N, 109.68° E): 5 ♂♂, 30-V~1-VI-2010, Bingbing Hu and Jing Zhang (BX15764); 1 ♂, 2 ♀♀, 740 m, 14~15-IV-2009, Qing Jin and Bingbing Hu (BX15763); 1 ♂, 738 m, 27~30-VII-2016.VII, Xia Bai, Shuonan Qian, and Wanding Qi. Mt. Diaoluo: 1 ♂, Nanxi Nature Reserves (18.66° N, 109.93° E), 250 m, 22-IV-2008, Bingbing Hu and Haiyan Bai. Mt. Jianfeng (18.75° N, 108.87° E): 2 ♀♀, 745 m, 11-VIII-2017, Xia Bai, Ping Liu, and Shuai Yu; 5 ♂♂, 810 m, 12~16-VI-2018, Xia Bai, Ping Liu, and Shuai Yu (BX16444); 1 ♂, 1050 m, 27-IV-2014, Tengteng Liu, Wei Guan, and Xuemei Hu; Tianchi (18.73° N, 108.87° E), 787 m: 1 ♂, 13-I-2016, Tengkai Jian, Xia Bai, and Mengting Chen (BX15789); 1 ♂, 787 m, 6-III-2016, Qingyun Wang, Suran Li, and Shengnan Zhao (BX15785); 2 ♂♂, 3 ♀♀, 8-VIII-2016, Xia Bai, Shuonan Qian, and Wanding Qi (BX15790). Baoting County: 1 ♂, Qixianling National Forest Park (18.70° N, 109.68° E), 302 m, 10-VII-2015, Qingyun Wang, Suran Li, and Mengting Chen. Yunnan: 2 ♂♂, Botanic Garden, Mengla, 21-XI-1987, Houhun Li; 1 ♂, Guanping, Mengyang, 1200 m, 20-VIII-2005, Yingdang Ren; 2 ♂♂, 1 ♀, Bubang, Mengla, 650 m, 24~25-VIII-2005, Yingdang Ren [NKU].

**Host plant.** Myrtaceae: *Eugenia densiflora* Willed.

**Distribution.** China (Hainan, Taiwan, Yunnan), Indonesia, Myanmar, New Guinea, Sri Lanka, and Thailand.

#### 3.15.7. *Lobesia montana* Diakonoff, 1954

*Lobesia montana* Diakonoff, 1954: 35. TL: Indonesia, West Java, Mount Gede−Pangrango, Tjibodas. Holotype: female, RMNH. 

**Material examined.** Taiwan: 14 ♂♂, 3 ♀♀, Hualien Hsien, Losao, 1110 m, 2-IV-1984, A. Kawabe (Sun-261 ♂, USNM145144, YH Sun; Sun-262 ♀, no abdomen); 2 ♀♀, Taiwan, Taidong Co., Lanyu, Langdao, 2-V-2003, Y. F. Hsu; 1 ♀, Taiwan, Jiayi Co, Dapu, Zhutoushan, 17-X-2000, Y. F. Hsu [USNM].

**Distribution.** China (Taiwan) and Indonesia.

#### 3.15.8. *Lobesia postica* Bae, 1993

*Lobesia postica* Bae, 1993: 522. TL: Taiwan, Pintung Hsien, Kenting Park. Holotype: female, USNM. 

**Material examined.** Taiwan: holotype: ♀, Pintung Hsien, Kenting Park, 100 m, 26-27-III-1984, A. Kawabe (examined) [USNM].

**Distribution.** China (Taiwan).

#### 3.15.9. *Lobesia reliquana* (Hübner, [1825] 1816)

*Asthenia reliquana* Hübner, [1825] 1816: 381. TL: Europe. Syntype(s): unknown.

*Tortrix leucopterana* Frölich, 1828: 65. TL: Germany. Syntype(s): unknown.

*Cochylis fischerana* Treitschke, 1835: 145. TL: Germany. Syntype(s): TMB.

*Lobesia reliquana* (Hübner): Guenée, 1845: 297.

*Lobesia permixtana* (Hübner): Meyrick, 1895: 455.

*Lobesia (Lobesia) reliquana* (Hübner): Obraztsov, 1953: 91.

**Material examined.** Taiwan: 1 ♀, Sirin, 4-XI-1933, S. Issiki (Sun-263 ♀, USNM145145, YH Sun) [USNM].

**Host plants.** Rosaceae: *Prunus spinosa* Linn.; Compositae: *Solidago virgaurea* Linn.; Poaceae: *Sorphum vulgare* Linn.; Betulaceae: *Betula* spp. and *Quercus* spp.; Boraginaceae: *Anchusa officinalis* Linn.; Cupressaceae: *Juniperus* spp. and *Salix* spp.; Fagaceae: *Faugs* spp.

**Distribution.** China (Inner Mongolia, Shaanxi, Sichuan, Taiwan, Yunnan), Asia Minor, Europe, Japan, Korea, and Russia.

**Remarks.** This species is recorded for the first time in Taiwan.

#### 3.15.10. *Lobesia virulenta* Bae et Komai, 1991

*Lobesia (Lobesia) virulenta* Bae et Komai, 1991: 127. TL: Japan, “Honshu, Osaka Prefecture”. Holotype: male, OPU.

*Lobesia virulenta*: Li, 2001: 524.

**Material examined.** Anhui: 1 ♀, Tangkou Town, Huangshang City, 5-VIII-2004, Jiasheng Xu and Jialiang Zhang; 6 ♂♂, 1 ♀, Ke Village, Mt. Jiuhua, 8-VIII-2004, Jiasheng Xu and Jialiang Zhang; 1 ♂, Tanqiao Town, Huangshan City, 7-VIII-2004, Jiasheng Xu and Jialiang Zhang. Gansu: 4 ♂♂, 3 ♀♀, Bifenggou, Wen County, 860 m, 10~14-VII-2005, Haili Yu. Guizhou: 1 ♀, Heiwan, Mt. Fanjing, 530 m, 2-VI-2002, Xinpu Wang; 1 ♀, Heiwan, Jiangkou, 600 m, 27-VII-2001, Houhun Li and Xinpu Wang; 3 ♂♂, 6 ♀♀, Fangxiang Town, Leishan County, 900 m, 13~14-IX-2005, Jialiang Zhang. Henan: 2 ♂♂, 3 ♀♀, Xiaguan, Neixiang County, 650 m, 10~12-VII-1998, Houhun Li; 1 ♀, Huangshian, Xixia, 890 m, 19-VII-1998, Houhun Li. Hunan: 2 ♂♂, 2 ♀♀, Mt. Badagong, Sangzhi, 1250 m, 12~14-VIII-2001, Houhun Li and Xinpu Wang; 1 ♂, 2 ♀♀, Zhangjiajie, 650 m, 7~8-VIII-2001, Houhun Li and Xinpu Wang. Sichuan: 7 ♂♂, 2 ♀♀, Baoxing County, 1100 m, 1~5-VIII-2004, Yingdang Ren; 5 ♂♂, 2 ♀♀, Fengyongzhai, Baoxing County, 1600 m, 2~3-VIII-2004, Yingdang Ren; 3 ♀♀, Labahe, Tianquan, 1300 m, 29-VII-2004, Yingdang Ren; 1 ♂, Mt. Qingcheng, 13-VIII-1990, unknown [NKU].

**Host plants.** Rosaceae: *Pyrus* sp. and *Pyrus serotina* Linn.; Pinaceae: *Larix leptolepis* (Siebold et Zucc.); Apiaceae: *Angelica* sp.; Styracaceae: *Styrax japonicus* Siebold et Zucc.

**Distribution.** China (Aanhui, Fujian, Gansu, Guizhou, Heilongjiang, Henan, Hunan, Shanghai, Sichuan, Taiwan, Zhejiang), Japan, Korea, and Russia.

### 3.16. Megalota *Diakonoff, 1966*

*Megalota* Diakonoff, 1966: 52. Type-species: *Polychrosis fallax* Meyrick, 1909.

**Remarks.** No species had been recorded in China prior to this study.

#### 3.16.1. *Megalota fallax* (Meyrick, 1909)

*Polychrosis fallax* Meyrick, 1909: 587. TL: India, Assam, Khasi Hills. Lectotype: male, BMNH.

*Megalota fallax* (Meyrick): Diakonoff, 1966: 52.

**Material examined.** Taiwan: 2 ♀, Kaohsiung Co. Santimen For. Sta., 9 km SE. Lukuei, 700 m, 4-7-XI-1984, J. Heppner and H. Wang (TORT12132 ♀, FL260) [FSCA].

**Distribution.** China (Taiwan) and India.

**Remarks.** This species is recorded for the first time in China.

### 3.17. Neopotamia *Diakonoff, 1973*

*Neopotamia* Diakonoff, 1973: 296. Type-species: *Neopotamia leucotoma* Diakonoff, 1973.

#### 3.17.1. *Neopotamia cryptocosma* Diakonoff, 1973

*Neopotamia cryptocosma* Diakonoff, 1973: 303. TL: India, Sikkim. Holotype: male, BMNH. 

*Neopotamia cryptocosma taiwana* Kawabe, 1992: 359. TL: Taiwan. Hualien Hsien, Kuanyuan. Holotype: male, USNM.

**Material examined.** Taiwan: 5 ♂♂, 3 ♀♀, Hualien Hsien, Kuanyuan, 2400 m, 7~8-VIII-1987, A. Kawabe (Sun-265 ♀, USNM90839, A. Kawabe; USNM90838 ♀, A. Kawabe); 1 ♂, Hualien Hsien, Wenshan spa, 580 m, 4~5-VIII-1983, A. Kawabe (USNM90833 ♂, A. Kawabe); 1 ♂, Nantou Hsien, Lushan spa, 1200 m, 27~29-VII-1983, A. Kawabe; 1 ♂, Nantou Hsien, Lushan spa, 1200 m, 3~5-VIII-1985, A. Kawabe; 1 ♂, Nantou Hsien, Yushih, 1700 m, 3~4-VIII-1987, A. Kawabe; 1 ♀, Nantou Hsien, Wushe, 1150 m, 27-XII-1988, A. Kawabe (USNM90840 ♀, A. Kawabe); 1 ♂, (Alishan) Chiai Hsien, Formosa, 12-VIII-1974, Y. Kishida (USNM90835 ♂, A. Kawabe); 1 ♀, Dayudian [Tai Yu Peak], 29-12-1985; USNM90836, A. Kawabe; paretype, A. Kawabe; 1 ♀, Hualien Hsien, Tayuling, 2560 m, 2~3-VIII-1983, A. Kawabe; 1 ♀, Nantow Co., Mei-feng, 30 km S Tayuling, 2200 m, 1~8-VI-1980, D. R. Davis; 3 ♂♂, 2 ♀♀, Nantow Co, Tayuling, 2500 m, 9~18-VI-1980, D. R. Davis (Sun-264 ♂, USNM90837, A. Kawabe; USNM90834 ♂, A. Kawabe); 1 ♂, Taiwan, Mareppa, 13-VIII-1943, A. Mutuura; 1 ♂, Taiwan, Tattaka, 19-VII-1925, S. Issiki; 1 ♀, Taiwan, Rarasan, 27-VI-1943, S. Issiki [USNM]. 

23 ♂♂, 2 ♀♀, Hsinchu Co. Kuangwu For. Sta., 2000 m, 18~25-VIII-1988, J. Heppner and H. Wang (TORT11835 ♂, FL063); 2 ♂♂, 1 ♀, Hsingchu/Miaoli Kuangwu, 2000 m, 24-25-VI-1985, J. Heppner and H. Wang; 1 ♂, Nantou Co. Tayuling, 2570 m, 5-9-VI-1982, J. Heppner; 1 ♂, 1 ♀, Kaohsiung Co. Tienchih, 2210 m, 24-X-1984, J. Heppner and H. Wang; 1 ♂, Kaohsing Co. Tienchih, Yushan N.P., 2280 m, 7-VII-1996, J. Heppner and H. Wang; 1 ♂, Hualien Co. Kuyuan, Taroko Gorge, 750 m, 6-7-VII-2005, J. Heppner; 1 ♂, Ilan Co., Taipingshan, 1900 m, 13-V-1989, J. Heppner and H. Wang; 1 ♂, Taitung Co. Yakou, 2750 m, 25-X-1984, J. Heppner and H. Wang; 1 ♂, Nantou Co. Tayuling, 2570 m, 15-18-VI-1982, J. Heppner; 2 ♂♂, 2 ♀♀, Kaohsiung Co. Tienchih, 2210 m, 24-X-1984, J. Heppner and H. Wang (TORT11836 ♀, FL064); 5 ♂♂, 2 ♀♀, Taitung Co. Yakou, 2750 m, 25-X-1984, J. Heppner and H. Wang (TORT11837 ♂, FL065; TORT11838 ♀, FL066); 1 ♂, Nantou Co., Meifeng, 2100 m, 6-9-IX-1988, J. Heppner; Gen. Sl. Bae UFL-026 ♂ [FSCA]. 

3 ♂♂, Zhongzhiguan, Gaoxiong City, 2100 m, 28-VII-2006, Houhun Li and Xicui DU [NKU].

**Distribution.** China (Sichuan, Taiwan, Xizang, Yunnan) and India, Sikkim.

#### 3.17.2. *Neopotamia divisa* (Walsingham, 1900)

*Phaecadophora divisa* Walsingham, 1900: 132. TL: India, Assam, Naga Hills. Holotype: female, BMNH. 

*Argyroploce acrosema* Meyrick, 1909: 601. TL: India. Assam, Khasi Hills. Lectotype: male, BMNH.

*Neopotamia divisa* (Walsingham): Heppner, 2012: 18.

**Material examined.** Taiwan: 1 ♀, Aizi Yue [in Chinese], 30-III-47 (Sun-266 ♀, USNM92976, A. Kawabe) [USNM].

**Distribution.** China (Taiwan) and India.

#### 3.17.3. *Neopotamia formosa* Kawabe, 1989

*Neopotamia formosa* Kawabe, 1989: 39. TL: Thailand, Nakhon Nayok, Khao Yai. Holotype: male, OPU.

**Material examined.** Taiwan: paratypes: 2 ♂♂, Nantow Co. Sunmoon Lake, 760 m, 20-25-VI-1980, D. R. Davis (Sun-267 ♂, USNM90843, A. Kawabe) [USNM].

Guangxi: 1 ♂, Xiangfen Town, Rongshui County, 650 m, 16-VII-2004, Jiasheng Xu [NKU].

**Distribution.** China (Guangxi, Taiwan) and Thailand.

#### 3.17.4. *Neopotamia ioxantha* (Meyrick, 1907)

*Enarmonia ioxantha* Meyrick, 1907: 139. TL: India, Assam, Khasi Hills. Lectotype: male, BMNH.

*Neopotamia ioxantha* (Meyrick): Brown, 2005: 429.

**Material examined.** Taiwan: 1 ♂, X-1926, S. Issiki (Sun-268 ♂, USNM145146, YH Sun) [USNM].

**Distribution.** China (Taiwan) and India.

**Remarks.** This species is recorded for the first time in China.

#### 3.17.5. *Neopotamia punctata* Kawabe, 1989 

*Neopotamia punctata* Kawabe, 1989: 38. TL: Thailand, Nakhon Nayok, Khao Yai. Holotype: male, OPU. 

**Material examined.** Taiwan: 1 ♂, Nantou Hsien, Lushan spa, 1200 m, 27-29-VII-1983, A. Kawabe (Sun-269 ♂, USNM90842, A. Kawabe) (paratype) [USNM].

Hainan: 1 ♂, Mingfenggu (18.74° N, 108.84° E), 954 m, 9-VIII-2017, Xia Bai, Ping Liu, and Shuai Yu; 1 ♂, Jianfengling Nature Reserves (18.74° N, 108.87° E), 770 m, 15-VII-2014, Peixin Cong, Linjie Liu, and Sha Hu (BX15442); 1 ♂, Jianfengling Nature Reserves (18.74° N, 108.87° E), 770 m, 3-VI-2015, Peixin Cong, Wei Guan, and Sha Hu (BX15651) [NKU]. 

**Distribution.** China (Hainan, Taiwan) and Thailand.

#### 3.17.6. *Neopotamia rubra* Kawabe, 1992

*Neopotamia rubra* Kawabe, 1992: 358. TL: Taiwan, Huslien Hsien, Hohuanshan. Holotype: male, USNM.

**Material examined.** Taiwan: holotype: ♂, Hualien Hsien, Hohuanshan, 3100 m, 30-VII-1-VIII-1983, A. Kawabe (USNM92973, A. Kawabe) (examined). Paratypes: 1 ♀, Nantou Hsien, Lushan spa, 1200 m, 3-5-VIII-1985, A. Kawabe; 1 ♀, Nantou Hsien, Tsuifeng, 2400 m, 29-XII-1989, A. Kawabe; 5 ♂♂, 9 ♀♀, Chiai Hsien, Alishan, 2270 m, 8-10-VIII-1985, A. Kawabe (Sun-271 ♀, USNM92974b, A. Kawabe); 1 ♂, 2 ♀♀, Hualien Hsien, Hohuanshan, 3100 m, 30-VII-1-VIII-1983, A. Kawabe (Sun-272 ♂; Sun-273 ♀); 1 ♂, Mt. Foufuanshan, Nantow, Formosa, 3100 m, 8-VIII-1974, Y. Kishida (Sun-270 ♂, USNM92974a, A. Kawabe); 1 ♀, (Alishan) Chiai Hsien, Formosa, 12-VIII-1974, Y. Kishida; 1 ♂, 1 ♀, Nantow Co. Mei-feng, 30 km S Tayuling, 2200 m, 1-8-VI-1980, D. R. Davis; 1 ♂, Nantow Co. Tayuling, 2500 m, 9-18-VI-1980, D. R. Davis; 1 ♂, Musya, 18-VII-1925, S. Issiki (examined) [USNM]. 

1 ♂, Hsinchu Co. Kuangwu For. Sta., 2000 m, 18-25-VIII-1988, J. Heppner and H. Wang; 5 ♂♂, 6 ♀♀, Nantou Co., Meifeng, 2100 m, 6-9-IX-1988, J. Heppner (Gen. Sl. Bae UFL-023 ♂); 6 ♂♂, 11 ♀♀, Taitung Co. Yakou, 2750 m, 25-X-1984, J. Heppner and H. Wang; 4 ♂♂, 1 ♀, Taitung Co. Chingshan, 1100 m, 31-VIII/4-IX-1988, J. Heppner and H. Wang; 1 ♂, Hsinchu Co. Kuangwu For. Sta., 2000 m, 18-25-VIII-1988, J. Heppner and H. Wang; 2 ♂♂, 1 ♀, Nantou Co. Tayuling, 2570 m, 5-9-VI-1982, J. Heppner (FSCA).

**Distribution.** China (Taiwan).

### 3.18. Olethreutes *Hübner, 1822*

*Olethreutes* Hübner, 1822: 72. Type-species: *Phalaena arcuella* Clerck, 1759.

*Roxana* Stephens, 1834: 118. Type-species: *Phalaena arcuella* Clerck, 1759. 

*Mixodia* Guenée, 1845: 160. Type-species: *Pyralis schulziana* Fabricius, 1776. 

*Exartema* Clemens, 1860: 356. Type-species: *Exartema nitidana* Clemens, 1860. 

#### 3.18.1. *Olethreutes aliana* Kawabe, 1993

*Olethreutes aliana* Kawabe, 1993: 236. TL: Taiwan, Hualien Hsien, Hohuanshan. Holotype: male, USNM.

**Material examined.** Taiwan: 1 ♂, Hualien Hsien, Houhuanshan, 3100 m, 10-11-VI-1988, K. Fujisawa (USNM92957 ♂, A. Kawabe) [USNM].

**Distribution.** China (Taiwan).

#### 3.18.2. *Olethreutes cana* Kawabe, 1993

*Olethreutes cana* Kawabe, 1993: 236. TL: Taiwan, Hualien Hsien, Tayuling. Holotype: male, USNM.

**Material examined.** Taiwan: 1 ♂, Nantow Co. Tayuling, 2500 m, 9-18-VI-1980, D. R. Davis (USNM92958 ♂, A. Kawabe) (holotype); paratypes: 1 ♀, Tayuling, Hualien Hsien, 28-VII-1985, S. Kimata; 1 ♀, Nantow Co. Tayuling, 2500 m, 9-18-VI-1980, D. R. Davis (USNM92959 ♀, A. Kawabe); 1 ♂, Hualien Hsien, Tayuling, 2560 m, 12-VI-1988, K. Fujisawa, no abdomen; 3 ♂♂, Nantow Co. Tayuling, 2500 m, 9-18-VI-1980, D. R. Davis, no abdomen; 1 ♂, Taiwan, Sakuramine, 8-VI-1943, S. Issiki; 1 ♂, Taiwan, Tipononsen, 4-VII-1927, S. Issiki [USNM]. 

8 ♂♂, 4 ♀♀, Nantou Co. Tayuling, 2570 m, 10-14-VI-1982, J. Heppner (Gen. Sl. Bae UFL-115 ♂; TORT12002 ♂, FL152; Gen. Sl. Bae UFL-010; TORT12001 ♀, FL153); 1 ♀, Taitung Co. Yakou, 2750 m, 25-X-1984, J. Heppner and H. Wang [FSCA].

**Distribution.** China (Taiwan).

#### 3.18.3. *Olethreutes doubledayana* (Barrett, 1872)

*Sericoris doubledayana* Barrett, 1872: 246. TL: Great Britain. Syntype(s): BMNH.

*Loxoterma doubledayana* (Barrett): Razowski, 1995: 314.

*Olethreutes doubledayana* (Barrett): Byun et al., 1998: 124.

**Material examined.** Taiwan: 1 ♂, Kagi, 28-XI-1934, S. Issiki; 1 ♂, Taihoku, Formosa, 8-VIII-1933, M. Chujo, Sun-274; 1 ♂, Taihoku, A-V-1921, S. Issiki; 1 ♂, Nantow Co., Lienhuachi Exp. For. Sta., 15 km SW Puli, 750 m, 22-26-V-1980, D. R. Davis; 1 ♂, Taiwan, Raisya, 25-XI-1934, S. Issiki; 1 ♂, Taiwan, Heito, 15-VIII-1925, S. Issiki [USNM].

**Host plants.** Fabaceae: *Trifolium repens* Linn. and *Dorycnium* spp.; Cyperaceae: *Cyperus longus* Linn.; Crassulaceae: *Sedum* spp.

**Distribution.** China (Anhui, Hebei, Heilongjiang, Henan, Hubei, Jilin, Tianjin, Shaanxi, Taiwan), Europe, Japan, Korea, and Russia.

**Remarks.** This species is recorded for the first time in Taiwan.

#### 3.18.4. *Olethreutes electana* (Kennel, 1901) 

*Penthina electana* Kennel, 1901: 257. TL: Russia, Primorsky Krai, Suchan. Holotype: female, MNHU. 

*Olethreutes electana* (Kennel): Razowski, 1971: 533.

*Phiaris electana* (Kennel): Byun et al., 1998: 133.

*Celypha electana* (Kennel): Kuznetzov, 2001: 273.

**Material examined.** Taiwan: 1 ♂, Higasinoko, 3-VI-1943, S. Issiki (Sun-275 ♂) [USNM]. 

2 ♂♂, Hualien Co. Kuanyuan, Taroko N.P., 2375 m, 3-VII-1996, J. Heppner and H. Wang (Gen. Sl. Bae UFL-008 ♂); ♂, Nantou Co. 15 km E of Puli, 700 m, 6-V-1989, J. Heppner and H. Wang (Gen. Sl. Bae UFL-080 ♂) [FSCA].

**Host plants.** Saxifragaceae: *Deutzia* spp.; Poaceae: *Filipendula palmata* (Pall) Maxim.

**Distribution.** China (Anhui, Beijing, Gansu, Hebei, Heilongjiang, Henan, Jilin, Sichuan, Taiwan, Tianjin, Yunnan, Zhejiang), Japan, and Russia (Far East).

#### 3.18.5. *Olethreutes flammanus* Kawabe, 1993

*Olethreutes flammanus* Kawabe, 1993: 231. TL: Taiwan, Hualien Hsien, Hohuanshan. Holotype: male, USNM.

**Material examined.** Taiwan: holotype: ♂, Hualien Hsien, Hohuanshan, 3100 m, 30-VII-1-VIII-1983, A. Kawabe (USNM92951 ♂, A. Kawabe) (examined). Paratypes: 1 ♀, Hualien Hsien, Tayuling, 2560 m, 2-3-VIII-1983, A. Kawabe (Sun-276 ♀, USNM92952) (examined) [USNM]. 

4 ♂♂, Nantou Co. Tayuling, 2570 m, 5-9-VI-1982, J. Heppner (TORT11850 ♂, FL036; TORT12161 ♂, Gen. Sl. Bae UFL-030); 1 ♂, Hsinchu Co. Kuangwu For. Sta., 2000 m, 18-25-VIII-1988, J. Heppner and H. Wang (TORT12162 ♂, Gen. Sl. Bae UFL-094) [FSCA].

**Distribution.** China (Taiwan).

#### 3.18.6. *Olethreutes meifengensis* Kawabe, 1993

*Olethreutes meifengensis* Kawabe, 1993: 231. TL: Taiwan, Nantou Hsien, Mei−feng, 30 km S Tayuling. Holotype: male, USNM.

**Material examined.** Yunnan: 6 ♂♂, 1 ♀, Zhongdian, 3150 m, 14-VII-2001, Houhun Li and Xinpu Wang [NKU].

**Distribution.** China (Taiwan, Yunnan).

**Remarks.** The authors have not seen the specimens collected from Taiwan.

#### 3.18.7. *Olethreutes orthocosma* (Meyrick, 1931)

*Argyroploce orthocosma* Meyrick, 1931: 141. TL: Japan, Tokyo and Hasimoto; China Kwanshien and Mt. Omei. Syntype(s): unknown. 

*Celypha orthocosma* (Meyrick): Razowski, 2000: 326

*Olethreutes orthocosma* (Meyrick): Kawabe, 1982: 109.

*Phiaris orthocosma* (Meyrick): Kuznetzov, 2001: 267.

**Material examined.** Taiwan: 3 ♂♂, Nantou Hsien, Lushan spa, 1200 m, 9-10-VIII-1987, A. Kawabe (Sun-277 ♂); 1 ♀, Nantou Hsien, Yushih, 1700 m, 3-4-VIII-1987, A. Kawabe; 2 ♂♂, Nantou Hsien, Lushan spa, 1200 m, 3-5-VIII-1985, A. Kawabe; 1 ♂, Nantou Hsien, Wushe, 1000 m, 6-7-VIII-1985, A. Kawabe; 6 ♂♂, Nantou Hsien, Lushan spa, 1200 m, 27-29-VII-1983, A. Kawabe; 1 ♂, Huali Hsien, Hungyeh spa, 200 m, 29-30-III-1984, A. Kawabe; 1 ♀, Hualien Hsien, Hungyeh spa, 200 m, 29-III-86, Y. Shibata; 1 ♂, Nantou Hsien, Wushe, 1150 m, 1-V-1990, A. Kawabe; 1 ♂, Nantow Co. Lienhuachi Exp. For. Sta. 15 km SW Puli, 750 m, 22-26-V-1980, D. R. Davis; 1 ♂, Koantauchi, Formosa, 31-X-1972, S. Yamane; 1 ♂, Lushan Nan Tow Hsien, Formosa, 4-VIII-1974, H. Nakajima; 1 ♂, Hualien Hsien, Tayuling, 2560 m, 12-VI-1988, K. Fujisawa; 1 ♂, Taiwan, Taiheisan, 9-V-1942, S. Issiki; 3 ♂, 1 ♀, Taiwan, Taiheisan, 9-V-1942, S. Issiki; 1 ♂, Nantou Hsien, Tsuifeng, 2400 m, 29-XII-1989, A. Kawabe; 1 ♂, 1 ♀, Suiryuto, 12-X-1935, S. Issiki; 4 ♂♂, Nantou Hsien, Nanshanchi, 25-26-VIII-1983, A. Kawabe; 2 ♂♂, Miaoli Hsien, Hernglong Lodge, 550 m. nr Taian spa, 8-VIII-1983, A. Kawabe [USNM]. 

4 ♂♂, Taichung Co. Chingshan, 1100 m, 31-VIII/4-IX-1988, J. Heppner and H. Wang (TORT12069 ♂, FL214); 2 ♂♂, Ilan Co. Tuchan, 480 m, 1-2-VII-1982, J. Heppner; 1 ♂, Taichung Co. Anmah-shan, 2250 m, 7-V-1989, J. Heppner and H. Wang; 1 ♂, 3 ♀♀, Taichung Co. Chingshan, 1100 m, 8-11-V-1989, J. Heppner and H. Wang; TORT12073 ♂, FL179; TORT12072 ♀, FL180); 1 ♀, Nantou Co. 15 km E of Puli, 700 m, 6-V-1989, J. Heppner and H. Wang (TORT12077 ♀, FL219); 46 ♂♂, 12 ♀♀, Kaohsiung Co. Liukuei, Shanpin For., 750 m, 16-23-III-1990, J. Heppner and H. Wang (TORT12070 ♂, FL270; TORT12120 ♀, FL271) [FSCA].

Anhui: 1 ♂, Tangkou Town, Huangshan City, 30-V-1987, unknown [NKU].

**Host plants.** Viburnaceae: *Viburnum furcatum* Blume; Rosaceae: *Fragaria* spp.; Labiatae: *Perilla frutescens* var. *japonica* (Hassk.).

**Distribution.** China (Anhui, Fujian, Guizhou, Sichuan, Taiwan), Korea, Japan, and Russia.

#### 3.18.8. *Olethreutes perdicoptera* (Wileman et Stringer, 1929)

*Tortrix perdicoptera* Wileman et Stringer, 1929: 65. TL: Taiwan, Formosa [Taiwan] (Rantaizan). Holotype: female, BMNH. 

*Olethreutes perdicoptera* (Wileman et Stringer): Kawabe, 1992: 107.

**Material examined.** Taiwan: 16 ♂♂, Nantow Co. Mei-feng, 30 km S Tayuling, 2200 m, 1-8-VI-1980, D. R. Davis (Sun-278 ♂, USNM145147, YH Sun; Sun-279 ♀, USNM145148, YH Sun); 5 ♂♂, Nantow Co. Tayuling, 2500 m, 9-18-VI-1980, D. R. Davis; 1 ♀, Hualien Hsien, Tayuling, 2560 m, 2-3-VIII-1983, A. Kawabe; 1 ♀, Hualien Hsien, Kuanyuan, 2400 m, 7-8-VIII-1987, A. Kawabe; 1 ♂, Nantou Hsien, Lushan spa, 1200 m, 7-8-VI-1988, K. Fujisawa; 1 ♀, Hualien Hisen, Tayuling, 2560 m, 12-VI-1988, K. Fujisawa; 1 ♂, 4 ♀♀, Alishan, Chiayi, Formosa, 2200 m, 9-11-VII-1964, H. Inoue; 1 ♀, Hualien Hsien, Tayuling, 2560 m, 2-3-VIII-1983, A. Kawabe; 2 ♂♂, Taiheisan, 17-V-1942, S. Issiki; 1 ♂, Nantou, 4-VI-1943, S. Issiki; 1 ♂, Taiwan, Rantaisan, 4-VI-1927, S. Issiki; 1 ♀, Taiwan, Rantaisan, 15-V-1933, S. Issiki [USNM]. 

2 ♂♂, 1 ♀, Nantou Co. Tayuling, 2570 m, 5-9-VI-1982, J. Heppner; 4 ♂♂, 4 ♀♀, Nantou Co. Tayuling, 2570 m, 10-14-VI-1982, J. Heppner; 2 ♂♂, 4 ♀♀, Ilan Co., Taipingshan, 1900 m, 13-V-1989, J. Heppner and H. Wang; 1 ♀, Taoyuan Co. Upper Palin, 1450 m, 14-15-V-1989, J. Heppner and H. Wang; 3 ♂♂, 2 ♀♀, Hsinchu/Miaoli Kuangwu, 2000 m, 24-25-VI-1985, J. Heppner and H. Wang; 4 ♂♂, 2 ♀♀, Hualien Co. 5 km SW of Tzuen, 2400 m, 11-V-1989, J. Heppner and H. Wang; 3 ♂, Hualien Co. Kuanyuan, Taroko N.P., 2375 m, 3-VII-1996, J. Heppner and H. Wang (Gen. Sl. Bae UFL-061 ♂; TORT12053 ♂, FL224); 1 ♂, Kaohsiung Co. Tienchih, Yushan N.P., 2280 m, 7-VII-1996, J. Heppner and H. Wang; 1 ♂, 2 ♀♀, Hualien Co., Kuyuan, Taroko Gorge, 750 m, 6-7-VII-2005, J. Heppner [FSCA].

**Distribution.** China (Taiwan).

#### 3.18.9. *Olethreutes perexiguana* Kuznetzov, 1988 

*Olethreutes perexiguana* Kuznetzov, 1988: 174. TD: North Vietnam. Holotype: male, ZMAS. 

*Syricoris perexiguana*: Brown, 2005: 444.

**Material examined.** Taiwan: 2 ♂♂, 1 ♀, Hassenzan, June 6, 1942, S. Issiki (Sun-327 ♂); 1 ♀, Taiwan, Taiheisan, May 9, 1942, S. Issiki; 1 ♀, Nantou Hsien, Wushe, 1150 m, May 1, 1990, A. Kawabe; 1 ♂, Nantow Co. Mei-feng, 30 km S Tayuling, 2200 m, June 1-8, 1980, D. R. Davis [USNM]. 

2 ♂♂, Ilan Co. Fushan For. Sta, 650 m, 20-24-VII-1996, J. Heppner (TORT11979 ♂, FL139); 2 ♂♂, Nantou Co. 5 km N. Chitou, 500 m, 29-VI-1985, J. Heppner and H. Wang; 1 ♂, Taipei Co. Wulai, 200 m, 7-19-VI-1985, J. Heppner and H. Wang (no photo, forewing pattern not clear, FL222 ♂); 1 ♀, Taitung Co. Bishan Hot Spgs., 720 m, 26-29-X-1984, J. Heppner and H. Wang (TORT12088 ♀, FL223); 1 ♂, Nantou Co. Lienhuachih For. Sta. Puli, 700 m, 4-VII-1996, J. Heppner and H. Wang; 1 ♂, Hsinchu Co. Kuangwu For. Sta., 2000 m, 18-25-VIII-1988, J. Heppner and H. Wang (no photo, forewing broken, FL168 ♂); 1 ♀, Nantou Co., Meifeng, 2100 m, 6-9-IX-1988, J. Heppner; 1 ♀, Kaohsiung Co. Lukuei For. Sta., 750 m, 29-IV/3-V-1989, J. Heppner and H. Wang; 1 ♀, Hualien Co. Kuyuan, Taroko Gorge, 750 m, 6-7-VII-2005, J. Heppner (TORT12056 ♀, FL169); 1 ♀, Taichung Co. Chingshan, 1100 m, 31-VIII/4-IX-1988, J. Heppner and H. Wang (TORT12180 ♀, Gen. Sl. Bae UF-110) [FSCA].

**Distribution.** China (Anhui, Fujian, Guangxi, Guizhou, Taiwan, Zhejiang) and Vietnam.

**Remarks.** This species is recorded for the first time in China.

#### 3.18.10. *Olethreutes sayonae* Kawabe, 1993

*Olethreutes sayonae* Kawabe, 1993: 234. TL: Taiwan, Hualien Hsien, Hohuanshan. Holotype: male, USNM.

**Material examined.** Taiwan: holotype: ♂, Hualien Hsien, Hohuanshan, 3100 m, 30-VII-1-VIII-1983, A. Kawabe (USNM92953 ♂, A. Kawabe) (examined) [USNM]. 

1 ♂, Tipononsen, 4 July 1929, S. Issiki; 1 ♂, Taiwan, Hassenzan, June 4, 1942, S. Issiki; 1 ♂, Taiwan, Rarasan, 23 June 1943, S. Issiki; 3 ♂♂, Hualien Hsien, Hohuanshan, 3100 m, 30-VII-1-VIII-1983, A. Kawabe (Sun-280 ♂, USNM92955, A. Kawabe); 2 ♂, 1 ♀, Hualien Hsien, Hohuanshan, 3100 m, 5-6-VIII-1987, A. Kawabe (Sun-281 ♂, USNM92954, A. Kawabe; Sun-282 ♀, USNM92956, A. Kawabe) [USNM].

**Distribution.** China (Taiwan).

### 3.19. Ophiorrhabda *Diakonoff, 1966*

*Ophiorrhabda* Diakonoff, 1966: 47. Type-species: *Argyroploce ergasima* Meyrick, 1911.

*Cellifera* Diakonoff, 1968: 47. Type-species: *Polychrosis cellifera* Meyrick, 1912. 

*Didrimys* Diakonoff, 1973: 388. Type-species: *Platypeplus harmonica* Meyrick, 1905. 

*Lasiognatha* Diakonoff, 1973: 429. Type-species: *Lasiognatha quartaria* Diakonoff, 1973.

#### 3.19.1. *Ophiorrhabda mormopa* (Meyrick, 1906)

*Platypeplus mormopa* Meyrick, 1906: 136. TL: Sri Lanka, Ceylon [Sri Lanka] (Maskeliya). Lectotype: male, BMNH.

*Argyroploce mormopa* (Meyrick): Meyrick, 1926: 152.

*Olethreutes mormopa* (Meyrick): Clarke, 1958: 531.

*Hedya (Platypeplus) mormopa*: Diakonoff, 1968: 46, 301.

*Lasiognatha mormopa* (Meyrick): Diakonoff, 1973: 430.

*Ophiorrhabda mormopa* (Meyrick): Kawabe, 1992: 106.

**Material examined.** Taiwan: 1 ♂, Heito, 8-IV-1935, S. Issiki (Sun-284 ♂, USNM145149, YH Sun); 1 ♀, Hagi, 18-XII-1934, S. Issiki (Sun-285 ♀, USNM145150, YH Sun); 1 ♂, Kagi, 16-XII-1934, S. Issiki; 1 ♀, Heito, 17-IV-1935, S. Issiki; 1 ♂, Nantou Hsien, Lushan spa, 1200 m, 3-5-VIII-1985, A. Kawabe; 1 ♀, Orchid Isl. 4 km SW Hungtou narrow valley, 5-10 m, 16-20-VII-1980, D. R. Davis; 1 ♂, Kao Hsiung Co., 10-11 km NE Chiahsien, ca. 300 m, 3-8-VII-1980, D. R. Davis [USNM]. 

1 ♂, 2 ♀♀, Taipei Co. Wulai, 200 m, 17-19-VI-1985, J. Heppner and H. Wang [FSCA]. 

Hainan: Haikou City: 1 ♂, Jinniuling Park (20.00° N, 110.31° E), 23 m, 31-V-2018, Ping Liu, Xia Bai, and Shuai Yu. Mt. Limu (19.17° N, 109.73° E): 1 ♂, 607 m, 15-V-2015, Peixin Cong, Wei Guan, and Sha Hu; 2 ♂♂, 607 m, 15~18-VII-2016, Xia Bai, Shuonan Qian, and Wanding Qi; 1 ♂, 632 m, 5-I-2016, Kaijian Teng, Xia Bai, and Mengting Chen; 13 ♂♂, 610 m, 4~5-V-2017, Xiaofei Yang. Mt. Bawang: 1 ♂, Mt. Bawang (19.12° N, 109.08° E), 161 m, 21-VII-2014, Peixin Cong, Linjie Liu, and Sha Hu; 2 ♂♂, Yajia Hydropower Station (19.10° N, 109.11° E), 245 m, 7-V-2013, Yinghui Sun, Wei Guan, and Tengteng Liu; 5 ♂♂, Yajia Hydropower Station (19.08° N, 109.10° E), 261 m, 20~21-VII-2015, Qingyun Wang, Suran Li, and Mengting Chen. Dongfang City: 1 ♂, 1 ♀, Datian Nature Reserves (19.11° N, 108.79° E), 100 m, 26~27-IV-2009, Qing Jin and Bingbing Hu (BX15119); 1 ♂, Lemei Village (19.08° N, 108.84° E), 124 m, 8-VI-2018, Ping Liu, Xia Bai, and Shuai Yu. Mt. Yingge: Hongkan (19.08° N, 109.50° E): 1 ♂, 508 m, 17-VI-2015, Peixin Cong, Wei Guan, and Sha Hu; 3 ♂♂, 540 m, 24~27-VII-2015, Qingyun Wang, Suran Li, and Mengting Chen; 1 ♂, 540 m, 22-I-2016, Kaijian Teng, Xia Bai, and Mengting Chen; Nankai Station (19.07° N, 109.40° E): 6 ♂♂, 210 m, 11~16-VIII-2016, Xia Bai, Shunan Qian, and Wanding Qi; 1 ♂, 280 m, 9-V-2017, Xiaofei Yang; 5 ♂♂, Yinggezui (19.05° N, 109.57° E), 599 m, 27-VII~1-VIII-2017, Xia Bai, Ping Liu, and Shuai Yu. Baisha County: 6 ♂♂, Hongxin Village (19.07° N, 109.52° E), Yuanmen County, 445 m, 1~3-VIII-2015, Qingyun Wang, Suran Li, and Mengting Chen (BX15117); 10 ♂♂, Yaxing Village (19.02° N, 109.40° E), Nankai County, 321 m, 21-VI-2015, Peixin Cong, Wei Guan, and Sha Hu (BX15118); 1 ♂, Yaxing Village (19.02° N, 109.40° E), Nankai County, 312 m, 29-VIII-2015, Qingyun Wang, Suran Li, and Mengting Chen. Wanning City: 2 ♂♂, Sangengluo Town (18.86° N, 110.19° E), 185 m, 27-VIII-2017, Xia Bai, Ping Liu, and Shuai Yu. Wuzhishan City: 1 ♂, Wuzhishan National Park (18.88° N, 109.67° E), 766 m, 9-I-2016, Kaijian Teng, Xia Bai, and Mengting Chen; 3 ♂♂, Wuzhishan Nature Reserves (18.88° N, 109.65° E), 742 m, 18~22-V-2015, Peixin Cong, Wei Guan, and Sha Hu; 5 ♂♂, Wuzhishan Nature Reserves (18.90° N, 109.67° E), 738 m, 22~30-VII-2017, Xia Bai, Shuonan Qian, and Wanding Qi; Shuiman Town (18.88° N, 109.67° E): 1 ♂, 640 m, 16-V-2017, Xiaofei Yang; 5 ♂♂, 690 m, 6-VIII-2017, Xia Bai, Ping Liu and Shuai Yu; 4 ♂♂, 766 m, 5~6-VII-2015, Qingyun Wang, Suran Li, and Mengting Chen; 4 ♂♂, 766 m, 1~4-VIII-2016, Xia Bai, Shuonan Qian, and Wanding Qi; 1 ♂, 690 m, 6-XI-2016.XI.6, Xia Bai, Shuonan Qian, and Wanding Qi. Mt. Diaoluo: 1 ♂, Nanxi Protection Station (18.66° N, 109.93° E), 250 m, 22-IV-2008, Bingbing Hu and Haiyan Bai. Ledong County: 1 ♂, Jianfeng Town (18.70° N, 108.79° E), 40 m, 28-IV-2013.IV.28, Yinghui Sun, Wei Guan, and Tengteng Liu; 1 ♂, Jianfengling (18.75° N, 108.87° E), 745 m, 11-VIII-2017, Xia Bai, Ping Liu, and Shuai Yu; 1 ♂, Tianchi (18.74° N, 108.84° E), 21-IV-2010, Bingbing Hu and Jing Zhang; 1 ♂, Tianchi (18.74° N, 108.84° E), 787 m, 12-VII-2015, Qingyun Wang, Suran Li, and Mengting Chen; 2 ♂♂, Tianchi (18.73° N, 108.87° E), 787 m, 8-VIII-2016, Xia Bai, Shuonan Qian, and Wanding Qi; 3 ♂♂, Jianfengling Nature Reserves (18.74° N, 108.87° E), 770 m, 28-V~5-VI-2015. Yunnan: 1 ♂, Botanic Garden, Menglun Town, 570 m, 15-VIII-2005, Yingdang Ren; 3 ♂♂, Guanping, Mengyang, 1200 m, 17 ~20-VIII-2005, Yingdang Ren; 1 ♂, Mt. Pinglong, Shangsi, 250 m, 7-IV-2002, Shulian Hao and Huaijun Xue. Taiwan: 1 ♂, Yongxing Farm, Lanyu County, 20 m, 20-VIII-2006, Houhun Li and Xicui Du (YHL07040) [NKU].

**Host plants.** Myrtaceae: *Syzygium iambos* Linn.; Rutaceae: *Citrus* spp.

**Distribution.** China (Guangxi, Hainan, Hongkong, Taiwan, Yunnan), Australia, Brunei, India, Indonesia, Japan, Malaysia, Nepal, the Philippines, Sri Lanka, Thailand, and Vietnam.

### 3.20. Pelatea *Guenée, 1845*

*Pelatea* Guenée, 1845: 161. Type-species: *Tortrix klugiana* Freyer, 1836.

*Petalea* Walker, 1866: 1790 [misspelling of *Pelatea*].

#### 3.20.1. *Pelatea assidua* (Meyrick, 1914)

*Argyroploce assidua* Meyrick, 1914: 49. TL: Taiwan, Formosa [Taiwan] (Suisharyo). Holotype: male, DEIB.

*Pelatea assidua* (Meyrick): Diakonoff, 1973: 503.

**Material examined.** Taiwan: 2 ♂♂, Chiai Hsien, Fenchihu, 1400 m, 11-12-VIII-1985, A. Kawabe (Sun-286 ♂, USNM122559, A. Kawabe; Sun-287 ♂, USNM122560, A. Kawabe; Sun-288 ♀, USNM122558, A. Kawabe); 1 ♂, Nantow Co. Mei-feng, 30 km S Tayuling, 2200 m, 1-8-VI-1980, D. R. Davis; 1 ♂, Taiwan, Hassenzan, 4-VI-1942, S. Issiki (Sun-332 ♂, USNM145151, YH Sun); 1 ♂, Taiwan, Higasinoko, 3-VI-1943, S. Issiki; 1 ♂, Taiwan, Kusukusu, 24-III-26, S. Issiki; 1 ♀, Taiwan, Kirisato, 1-VI-1943, S. Issiki (Sun-333 ♀, USNM145152, YH Sun); 1 ♀, Taiwan, Rantaisan, 4-VI-1927, S. Issiki; 1 ♀, Nantou Hsien, Tsuifeng, 2400 m, 29-XII-1989, A. Kawabe [USNM]. 

1 ♂, Kaosiung Co. Liukuei, Shanpin For., 750 m, 16-23-III-1990, J. Heppner and H. Wang (Gen. Sl. Bae UFL-051 ♂); 1 ♂, 4 ♀♀, Ilan Co. Mingchyr Rec. Area, 1250 m, 9-10-VII-1996, J. Heppner and H. Wang (TORT12051 ♂, FL170; TORT12058 ♀, FL171); 1 ♂, Ilan Co., Taipingshan, 1900 m, 13-V-1989, J. Heppner and H. Wang; 1 ♂, Chiayi Co. Fennchihwu, 1450 m, 2-4-VII-1985, J. Heppner and H. Wang; 4 ♂♂, Taoyuan Co. Upper Palin, 1500 m, 7-9-VII-1985, J. Heppner and H. Wang; 1 ♀, Taichung Co., Chingshan, 1100 m, 8-11-V-1989, J. Heppner and H. Wang (TORT12122 ♀) [FSCA].

**Distribution.** China (Taiwan).

### 3.21. Phaecadophora *Walsingham, 1900*

*Phaecadophora* Walsingham, 1900: 130. Type-species: *Phaecadophora fimbriata* Walsingham, 1900.

#### 3.21.1. *Phaecadophora acutana* Walsingham, 1900

*Phaecadophora acutana* Walsingham, 1900: 131 TL: Japan, Kyushu, Satsuma. Holotype: female, BMNH.

*Olethreutes acutana*: Inoue, 1954: 107.

**Distribution.** China (Taiwan) and Japan.

#### 3.21.2. *Phaecasiophora attica* (Meyrick, 1907)

*Eucosma attica* Meyrick, 1907: 137. TL: India, Assam, Khasi Hills. Lectotype: male, BMNH.

*Argyroploce attica* (Meyrick): Meyrick, 1935: 61.

*Olethreutes attica* (Meyrick): Clarke, 1958: 487.

*Phaecasiophora (Phaecasiophora) attica* (Meyrick): Diakonoff, 1959: 170.

*Phaecasiophora attica* (Meyrick): Brown, 2005: 485.

**Distribution.** China (Taiwan), India, Myanmar, Thailand, and Vietnam.

#### 3.21.3. *Phaecadophora fimbriata* Walsingham, 1900

*Phaecadophora fimbriata* Walsingham, 1900: 130 TL: Japan, Kyushu, Satsuma [Kagoshima Prefecture]. Lectotype: male, BMNH. 

*Argyroploce metactenis* Meyrick, 1909: 597. TL: India. Assam, Khasi Hills. Lectotype: male, BMNH.

*Argyroploce eucrossa* Meyrick, 1914: 49. TL: Taiwan. Formosa [Taiwan]. Holotype: male, DEIB.

*Argyroploce eaolotechna* Meyrick, 1935: 60. TL: China. Kiangsu Province, Lungtan bei Nanking. Lectotype: male, MGAB. 

*Argyroploce leucocteis* Diakonoff, 1953: 112. TL: New Guinea. Moss Forest Camp, 5 km NE Lake Habbema. Holotype: male, RMNH.

*Eudemis fimbriata*: Issiki, 1957: 69.

*Phaecadophora eaolotechna*: Clarke, 1958: 571.

*Phaecadophora aeolotechna*: Diakonoff, 1968: 57.

**Material examined.** Taiwan: 1 ♀, Tyokakurai, 25-III-44, S. Issiki; 1 ♂, Taihoku, 15-VII-1934, S. Issiki; 1 ♂, Taiwan; S. Issiki (Sun-289 ♂, USNM24106, UFGC); 1 ♂, Mt. Yuwan-dake, Is. Amami-oshima, 15-17-VIII-1977, A. Seino; 1 ♂, Yoshio, Ashikitamachi, Ashikita-gun, 18-IV-1917, Isao Ohtsuka [USNM]. 

3 ♂♂, 4 ♀♀, Taipei Co. Wulai, 200 m, 17-19-VI-1985, J. Heppner and H. Wang; 2 ♀♀, Ilan Co. Chilan Nsy., Tuchan, 480 m, 15-17-X-1984, J. Heppner and H. Wang; 1 ♂, Taitung Co. Yakou, 2750 m, 25-X-1984, J. Heppner and H. Wang; 1 ♂, Ilan Co. Chilan Nsy., Tuchan, 480 m, 12-V-1989, J. Heppner and H. Wang; 1 ♀, Taitung Co. Bishan Hot Spgs., 720 m, 26-29-X-1984, J. Heppner and H. Wang; 1 ♀, Kaohsiung Co. Dona, 16 km NE. Maolin, 500 m, 3-XI-1984, J. Heppner and H. Wang; 1 ♀, Ilan Co., Taipingshan, 1900 m, 13-V-1989, J. Heppner and H. Wang [FSCA].

Anhui: 1 ♂, 3 ♀♀, Tanqiao Town, Huangshan City, 6-VIII-2004; 1 ♂, Ke Village, Mt. Jiuhua, 8-VIII-2004; 1 ♂, Fengjing Village, Mt. Tianzhu, 10-VIII-2004; 1 ♂, Jiuhuajie, Mt. Jiuhua, 9-VIII-2004, Jiasheng Xu and Jialiang Zhang. Fujian: 1 ♀, Xianfengling, Mt. Wuyi, 1000 m, 26-V-2004; 1 ♀, Pikeng, Mt. Wuyi, 600 m, 27-V-2004, Haili Yu; 1 ♂, Mt. Qingyun, Yongtai County, 550 m, 18-IX-2002, Xinpu Wang. Guizhou: 1 ♀, Jinding, Mt. Fanjing, 2200 m, 30-V-2002, Xinpu Wang; 1 ♂, Dashahe, Daozhen County, 1350 m, 25-V-2004, Shulian Hao. Hainan: Mt. Limu: 2 ♂♂, Nature Reserves (19.17° N, 109.74° E), 640 m, 1-V-2014, Tengteng Liu, Wei Guan, and Xuemei Hu. Mt. Bawang: 1 ♂, Yajia Hydropower Station (19.10° N, 109.11° E), 245 m, 9-V-2013, Yinghui Sun, Wei Guan, and Tengteng Liu (BX15207). Mt. Yingge (19.08° N, 109.50° E): 1 ♂, 620 m, 3-VI-2010, Bingbing Hu and Jing Zhang. Baisha County: 3 ♂♂, Yaxing Village, Nankai Town (19.02° N, 109.40° E), 321 m, 21-VI-2015, Peixin Cong, Wei Guan, and Sha Hu. Wuzhishan City: 1 ♂, Lizudadian, Shuiman Town (18.88° N, 109.67° E), 766 m, 5-VII-2015, Qingyun Wang, Suran Li, and Mengting Chen; 1 ♀, Butterfly Pasture (18.87° N, 109.67° E), 680 m, 27-X-2016, Xia Bai, Shuonan Qian, and Wanding Qi (BX16493); 1 ♂, Shuiman Town (18.88° N, 109.67° E), 640 m, 28-X-2016, Xia Bai, Shuonan Qian, and Wanding Qi. Mt. Jianfeng: 7 ♂♂, Tianchi (18.73° N, 108.87° E), 787 m, 13~17-VII-2015, Qingyun Wang, Suran Li, and Mengting Chen. Hubei: 1 ♂, Maoba District, Lichuan City, 700 m, 30-VII-1999, Houhun Li. Hunan: 1 ♀, Cangxi Town, Xinhua County, 9-VIII-2004, Yunli Xiao [NKU].

**Host plant.** Fagaceae: *Quercus glauca* Thunb.

**Distribution.** China (Anhui, Fujian, Guangdong, Guangxi, Guizhou, Hainan, Hubei, Hunan, Jiangsu, Jiangxi, Taiwan, Yunnan), India, Indonesia, Japan, Papua New Guinea, Russia, Thailand, and Vietnam.

### 3.22. Phaecasiophora *Grote, 1873*

*Phaecasiophora* Grote, 1873: 90. Type-species: *Sciaphila confixana* Walker, 1863.

*Megasyca* Diakonoff, 1959: 171. Type-species: *Sericoris mutabilana* Clemens, 1865 [=*Sciaphila confixana* Walker, 1863] [subgenus of *Phaecasiophora*].

#### 3.22.1. *Phaecasiophora amoena* Kawabe, 1986

*Phaecasiophora amoena* Kawabe, 1986: 77. TL: Taiwan, Hualien Hsien, Hungyeh spa. Holotype: male, USNM.

**Material examined.** Taiwan: holotype: ♂, Hualien Hsien, Hungyeh spa, 200 m, 29-30-III-1984, A. Kawabe (examined) [USNM].

**Distribution.** China (Guangxi, Taiwan).

#### 3.22.2. *Phaecasiophora caryosema* (Meyrick, 1931)

*Argyroploce caryosema* Meyrick, 1931: 138. TL: Taiwan, Kyuhabon. Lectotype: male, BMNH. 

*Olethreutes caryosema* (Meyrick): Clarke, 1958: 492.

Phaecasiophora (Phaecasiophora) caryosema (Meyrick): Diakonoff, 1973: 118.

*Phaecasiophora caryosema* (Meyrick): Kawabe, 1992: 106.

**Material examined.** Taiwan: 1 ♀, Taiwan, Sinten, 11-IX-1925, S. Issiki (Sun-290 ♀, USNM145153, YH Sun); 1 ♂, Ilan Co. 5 km N. Nanao, 100 m, 11-13-X-1984, J. Heppner and H. Wang (TORT12110 ♂, FL267) [USNM].

**Distribution.** China (Taiwan).

#### 3.22.3. *Phaecasiophora cornigera* Diakonoff, 1959

*Phaecasiophora cornigera* Diakonoff, 1959: 180. TL: India, Assam, Khasis, Cherra Punji. Holotype: male, BMNH.

*Phaecasiophora cornigera* ssp. *burmensis* Diakonoff, 1959: 171. TL: India. Burma (Kambaiti). Holotype: male, NHRS.

*Phaecasiophora (Megasyca) cornigera* ssp. *birmensis* Diakonoff, 1973: 121. No type.

**Material examined.** Taiwan: 1 ♂, Nantou Hsien, Lushan spa, 1200 m, 9-10-VIII-1987, A. Kawabe; 2 ♂♂, Nantou Hsien, Yushih, 1700 m, 3-4-VIII-1987, A. Kawabe; 1 ♀, Formosa, Nantew Rozan, 29-IV-1-May, 1973, Mi. Yamamoto; 2 ♂♂, Nantou Hsien, Lushan spa, 1200 m, 27-29-VII-1983, A. Kawabe; ♂, Nantou Hsien, Lushan spa, 1200 m, 27-29-VII-1983, A. Kawabe (Sun-291 ♂, USNM122562, A. Kawabe); 2 ♂♂, Pingtung Co. Kenting Bot. Garden subtropical forest, 260 m, 22-25-VII-1980, D. R. Davis; 1 ♀, Nantow Co. Lienhuachi Exp. For. Sta. 15 km SW Puli, 750 m, 22-26-V-1980, D. R. Davis; 1 ♂, Taiwan; S. Issiki; 1 ♂, Taiwan, Habon, A-X-1926, S. Issiki [USNM]. 

Fujian: 4 ♂♂, Xianfengling, Mt. Wuyi, 1000 m, 26-V-2004, Haili Yu. Guangxi: 1 ♂, Jiuniu Tang, Mt. Maoer, 1100 m, 20-IV-2002, Shulian Hao and Huaijun Xue. Guizhou: 1 ♂, Dashahe, Daozhen County, 1420 m, 22-VII-2004, Shulian Hao. Hainan: Mt. Limu: 2 ♂♂, Forest Park (19.17° N, 109.73° E), 610 m, 4~5-V-2017, Xiaofei Yang. Mt. Yingge: 2 ♂♂, Yinggezui Station (19.05° N, 109.56° E), 700 m, 12~13-V-2017, Xiaofei Yang. Wuzhishan City: 1 ♂, Shuiman Town (18.88° N, 109.67° E), 1-VI-2010, Bingbing Hu and Jing Zhang; 2 ♂♂, Lizudadian, Shuiman Town (18.88° N, 109.67° E), 766 m, 3-VIII-2016, Xia Bai, Shuonan Qian, and Wanding Qi (BX15742, BX16482); 1 ♂, Nature Reserves, Mt. Wuzhi (18.88° N, 109.65° E), 742 m, 20-V-2015, Peixin Cong, Wei Guan, and Sha Hu; 1 ♂, Nature Reserves, Mt. Wuzhi (18.90° N, 109.67° E), 738 m, 24-VII-2016.VII.24, Xia Bai, Shuonan Qian, and Wanding Qi. Sichuan: 1 ♂, Laba River, Tianquan County, 1300 m, 29-VII-2004, Yingdang Ren. Taiwan: 1 ♂, 1 ♀, Mt. Lala, Nantou County, 1800 m, 1-VIII-2006, Houhun Li and Xicui Du. Zhejiang: 2 ♂♂, Wuyanling, Taishun County, 1000~1050 m, 30-VII~3-VIII-2005, Yunli Xiao [NKU].

**Distribution.** China (Fujian, Guangdong, Guangxi, Guizhou, Hainan, Hongkong, Sichuan, Taiwan, Zhejiang), India, Thailand, and Vietnam.

#### 3.22.4. *Phaecasiophora diluta* Diakonoff, 1973

*Phaecasiophora (Phaecasiophora) diluta* Diakonoff, 1973: 112. TL: India, Assam, Khasias hills. Holotype: male, BMNH.

**Material examined.** Taiwan: 2 ♂♂, Ilan Co., Taipingshan, 1900 m, 13-V-1989, J. Heppner and H. Wang (TORT12173 ♂, Gen. Sl. Bae UF-076; TORT12175 ♂, Gen. Sl. Bae UF-106) [FSCA].

**Distribution.** China (Fujian, Henan, Taiwan), India, and Indonesia.

**Remarks.** This species is recorded for the first time in Taiwan.

#### 3.22.5. *Phaecasiophora fernaldana* Walsingham, 1900

*Phaecasiophora fernaldana* Walsingham, 1900: 135 TL: Japan/Korea, Gensan. Syntypes: male and female, BMNH. 

*Phaecasiophora latior* Diakonoff, 1959: 180. TL: Indonesia. Central West Sumatra, Fort de Kock. Holotype: male, RMNH.

**Material examined.** Taiwan: 1 ♂, (Lushan spa) Nantow Hsien, Formosa, 6-VIII-1974, Y. Kishida; 7 ♂♂, Nantow Co. Meifeng, 30 km S Tayuling, 2200 m, 1-8-VI-1980, D. R. Davis; 1 ♂, Chiai Hsien, Fenchihu, 1400 m, 11-12-VIII-1985, A. Kawabe; 1 ♂, Chiduan [in Chinese], 23-IV-1983, B.S. Chang; 1 ♂, Rozan spa, Formosa, 27-VIII-1969, M. Nisikawa; 1 ♂, Taitung Hsien, Chihpen spa, 200 m, 28-III-1984, A. Kawabe (Sun-292 ♂, USNM122563, A. Kawabe); ♀, Fenchihu, Formosa, 10-IV-1965, T. Shirozu (Sun-293 ♀, USNM122564, A. Kawabe); 2 ♂♂, Nantou Hsien, Lushan spa, 1200 m, 3-5-VIII-1985, A. Kawabe; 1 ♂, Chiai Hsien, Alishan, 2270 m, 8-10-VIII-1985, A. Kawabe; 1 ♂, Nantou Hsien, Tsingjing Farm, 1900 m, 9-VI-1988, K. Fujisawa; 1 ♂, Nantou Hsien, Lushan spa, 1200 m, 9-10-VIII-1987, A. Kawabe; 1 ♂, Taoyuan Hsien, Palin, 1000 m, 31-VII-2-VIII-1985, A. Kawabe; 1 ♂, Nantou Hsien, Wushe, 1150 m, 1-V-1990, A. Kawabe; 1 ♂, Nantou Hsien, Tsingjing Farm, 1900 m, 9-VI-1988, K. Fujisawa; 2 ♂♂, Tattaka, 19-VII-1925, S. Issiki; 1 ♂, Sinten, 11-IX-1925, S. Issiki; 1 ♂, Taiwan; S. Issiki; 1 ♂, Taipin, May, T. Shiraki [USNM]. 

3 ♂♂, 1 ♀, Kaohsiung Co. Liukuei, Shanpin For., 750 m, 16-23-III-1990, J. Heppner and H. Wang; 4 ♂♂, Nantou Co. Meifeng Hort. Sta., 2100 m, 6-10-IX-1988, J. Heppner and H. Wang; 1 ♀, Taipei Co. Wulai, 200 m, 17-19-VI-1985, J. Heppner and H. Wang; 1 ♂, Hsinchu/Miaoli Kuangwu, 2000 m, 24-25-VI-1985, J. Heppner and H. Wang; 1 ♂, Taitung Co. Bishan Hot Spgs., 720 m, 26-29-X-1984, J. Heppner and H. Wang; 1 ♂, Hsinchu Co. Litonshan, 1450-1914 m, 13-VII-2005, J. Heppner; 2 ♂♂, Taichung Co. Chingshan, 1100 m, 31-VIII/4-IX-1988, J. Heppner and H. Wang; 1 ♂, Nantou Co. 12 km E. Puli, 700 m, 24-25-III-1990, J. Heppner and H. Wang; 1 ♂, Hualien Co. 7 km. E. Kuangfu, 300 m, 26-VIII-1983, J. Heppner; 1 ♀, Taoyuan Co. Upper Palin, 1350 m, 4-5-VII-2005, J. Heppner and H. Wang; 1 ♂, Hualien Co. Tzuen, 1975 m, 19-20-VI-1982, J. Heppner; 1 ♂, Hualien Co. Kuyuan, Taroko Gorge, 750 m, 6-7-VII-2005, J. Heppner [FSCA].

Guizhou: 1 ♂, Chishuisuoluo, 240 m, 21-IX-2000, Haili Yu. Hunan: 1 ♂, Zhangjiajie, 650 m, 7-VIII-2001, Houhun Li and Xinpu Wang. Sichuan: 8 ♂♂, 1 ♀, Fengyongzhai, Baoxing County, 1600 m, 3-VIII-2004, Yingdang Ren; 1 ♂, Laba River, Tianquan County, 1300 m, 29-VII-2004, Yingdang Ren [NKU].

**Distribution.** China (Anhui, Guizhou, Hunan, Sichuan, Taiwan), Japan, Korea, and Russia.

#### 3.22.6. *Phaecasiophora leechi* Diakonoff, 1973

*Phaecasiophora (Megasyca) leechi* Diakonoff, 1973: 120. TL: China (Foochau). Holotype: female, BMNH. 

*Phaecasiophora obligata* Kawabe, 1993: 227, 228.

*Phaecasiophora guttulosa* (Diakonoff): Liu et Li, 2002: 242, pls. XXXIII−328, LXXX−328.

*Phaecasiophora leechi* (Diakonoff): Brown, 2005: 486.

*Phaecasiophora (Phaecasiophora) leechi* (Diakonoff): Yu et Li, 2006: 36.

**Material examined.** Taiwan: holotype: ♂, Nantow Co. Lienhuachi Exp. For. Sta. 15 km SW Puli, 750 m, 22-26-V-1980, D. R. Davis (USNM92894, A. Kawabe) (examined) [USNM]. 

1 ♂, Rengwati, 29-III-27, S. Issiki (Sun-294 ♂); 1 ♂, Taiwan, Rengwati, 21-III-43, S. Issiki; 1 ♂, Taipei Co. Wulai, 200 m, 17-19-VI-1985, J. Heppner and H. Wang (Gen. Sl. Bae UF-113 ♂) [FSCA].

Anhui: 1 ♂, Ke Village, Mt. Jiuhua, 8-VIII-2004, Jiasheng Xu and Jialiang Zhang; 2 ♂♂, 1 ♀, Jiuhuajie, Mt. Jiuhua, 9-VIII-2004, Jiasheng Xu and Jialiang Zhang; 1 ♂, Tanqiao Town, Huangshan City, 6-VIII-2004, Jiasheng Xu and Jialiang Zhang; 1 ♂, Tangkou Town, Mt. Jiuhua, 5-VIII-2004, Jiasheng Xu and Jialiang Zhang. Fujian: 1 ♂, Xianfengling, Mt. Wuyi, 1000 m, 26-V-2004, Haili Yu. Hubei: 1 ♂, Maoba District, Lichuan County, 700 m, 30-VII-1999, Houhun Li. Hunan: 6 ♂♂, 4 ♀♀, Jianxin Village, Ketou Town, Xinhua County, 10~11-VIII-2004, Yunli Xiao; 1 ♀, Cangxi Town, Xinhua County, 9-VIII-2004, Yunli Xiao. Zhejiang: 1 ♂, Mt. Tianmu, 500 m, 16-VIII-1999, Houhun Li; 7 ♂♂, Wuyanling, Taishun County, 680~1050 m, 28-VII~2-VIII-2005, Yunli Xiao [NKU].

**Distribution.** China (Anhui, Fujian, Guangdong, Guangxi, Hongkong, Hubei, Hunan, Jiangxi, Taiwan, Zhejiang).

**Remarks.** This species is recorded for the first time in Taiwan.

#### 3.22.7. *Phaecasiophora obraztsovi* Diakonoff, 1973

*Phaecasiophora pryeri*: Issiki, 1957: 71.

*Phaecasiophora (Megasyca) obraztsovi* Diakonoff, 1973: 124. TL: Japan (Honshu, Kawati [Osaka Prefecture], Iwanaakisan). Holotype: male, LNK.

*Phaecasiophora obraztsovi*: Kawabe, 1982: 95.

**Material examined.** Taiwan: 1 ♂, Taichung Co. Chingshan, 1100 m, 31-VIII/4-IX-1988, J. Heppner and H. Wang; 1 ♂, Kaohsiung Co. Dona, 16 km NE. Maolin, 500 m, 3-XI-1984, J. Heppner and H. Wang; 1 ♂, Hsinchu Co. Kuangwu For. Sta., 2000 m, 18-25-VIII-1988, J. Heppner and H. Wang; 1 ♂, Hsinchu Co. Litonshan, 1450-1914 m, 13-VII-2005, J. Heppner (TORT12109 ♂, FL259) [FSCA].

**Host plant.** Fagaceae: *Quercus acutissima* Carr.

**Distribution.** China (Taiwan, Zhejiang), Japan, and Russia (Far East).

**Remarks.** This species is recorded for the first time in Taiwan.

#### 3.22.8. *Phaecasiophora pyragra* Diakonoff, 1973 

*Phaecasiophora (Megasyca) pyragra* Diakonoff, 1973: 122. TL: China (Hainan Island, Mangrin). Holotype: male, BMNH. 

*Phaecasiophora pyragra* (Diakonoff): Liu et Li, 2002: 242.

**Material examined.** Taiwan: 1 ♂, Nantou Co. Huisun For. Rec. Area, 700 m, 8-VIII-1988, J. Heppner (TORT12172, Gen. Sl. Bae UF-101) [FSCA].

**Distribution.** China (Guangxi, Hainan, Taiwan).

**Remarks.** This species is recorded for the first time in Taiwan.

#### 3.22.9. *Phaecasiophora walsinghami* Diakonoff, 1959

*Phaecasiophora (Megasyca) walsinghami* Diakonoff, 1959: 179. TL: Indonesia (West Java, Mt. Gede–Pangrango, Tjibodas). Holotype: male, RMNH.

*Phaecasiophora walsinghami* (Diakonoff): Kawabe, 1987: 64.

**Material examined.** Taiwan: 1 ♂, Habon, A-X-1926, S. Issiki (Sun-295 ♂, USNM145154, YH Sun) [USNM].

**Distribution.** China (Anhui, Guangxi, Guizhou, Hunan, Sichuan, Taiwan, Yunnan, Zhejiang), Indonesia, and Thailand.

**Remarks.** This species is recorded for the first time in Taiwan.

### 3.23. Phaulacantha *Diakonoff, 1973*

*Phaulacantha* Diakonoff, 1973: 185. Type-species: *Argyroploce catharostoma* Meyrick, 1921.

#### 3.23.1. *Phaulacantha acyclica* Diakonoff, 1973

*Phaulacantha acyclica* Diakonoff, 1973: 187. TL: Taiwan, Formosa [Taiwan] (Kyuhabon). Holotype: male, BMNH. 

**Material examined.** Taiwan: 3 ♂♂, Nantow Co. Mei-feng, 30 km S Tayuling, 2200 m, 1-8-VI-1980, D. R. Davis (Sun-296 ♂); 1 ♂, Hassenzan, 30-VIII-1929, S. Issiki (Sun-297 ♂, USNM145155, YH Sun) [USNM].

**Distribution.** China (Taiwan).

### 3.24. Proschistis *Meyrick, 1907*

*Proschistis* Meyrick, 1907: 731. Type-species: *Proschistis zaleuta* Meyrick, 1907.

*Sporocelis* Meyrick, 1907: 732. Type-species: *Sporocelis marmaropa* Meyrick, 1907. 

#### 3.24.1. *Proschistis marmaropa* (Meyrick, 1907)

*Sporocelis marmaropa* Meyrick, 1907: 732. TL: Sri Lanka, Ceylon [Sri Lanka] (Maskeliya). Lectotype: male, BMNH. 

*Proschistis marmaropa* (Meyrick): Diakonoff, 1973: 281.

**Material examined.** Taiwan: 1 ♂, Nantou Hsien, Lushan spa, 1200 m, 7-8-VI-1988, K. Fujisawa; 1 ♂, Nantow Co. Lienhuachi Exp. For. Sta. 15 km SW Puli, 750 m, 22-26-V-1980, D. R. Davis; 1 ♂, Kao Hsiung Co., 10-11 km NE Chiahsien, ca. 300 m, 3-8-VII-1980, D. R. Davis; 1 ♂, Huali Hsien, Hungyeh spa, 200 m, 29-30-III-1984, A. Kawabe (Sun-298 ♂, USNM145156, YH Sun) [USNM].

Hainan: Wuzhishan City: 2 ♂♂, Mt. Wuzhi (18.91° N, 109.67° E), 732 m, 2~5-VIII-2017, Xia Bai, Ping Liu, and Shuai Yu; 1 ♂, Lizudadian, Shuiman Town (18.88° N, 109.67° E), 766 m, 1-VIII-2016, Xia Bai, Shuonan Qian, and Wanding Qi; 2 ♂♂, Experimental Area, Wuzhishan Nature Reserves (18.91° N, 109.68° E), 710 m, 20~21-IV-2014, Tengteng Liu, Wei Guan, and Xuemei Hu (BX15363); 1 ♂, Wuzhishan Nature Reserves (18.90° N, 109.67° E), 738 m, 22-V-2015, Peixin Cong, Wei Guan, and Sha Hu; 2 ♂♂, 1 ♀, Wuzhishan Nature Reserves (18.90° N, 109.67° E), 738 m, 24-VII~3-XI-2016, Xia Bai, Shuonan Qian, and Wanding Qi (BX16495). Mt. Jianfeng: 2 ♂♂, Mingfenggu (18.74° N, 108.84° E), 954 m, 9~10-VIII-2017, Xia Bai, Ping Liu, and Shuai Yu [NKU]. 

**Distribution.** China (Hainan, Taiwan), Japan, and Sri Lanka.

#### 3.24.2. *Proschistis sideroxyla* (Meyrick, 1931)

*Argyroploce sideroxyla* Meyrick, 1931: 133. TL: Taiwan, Formosa [Taiwan] (Kyuhabon). Holotype: female, BMNH. 

*Proschistis sideroxyla* (Meyrick): Clarke, 1958: 580.

*Olethreutes sideroxyla* (Meyrick): Kawabe, 1992: 107.

**Material examined.** Taiwan: 1 ♀, Habon, A-X-1926, S. Issiki (Sun-300 ♀, USNM145157, YH Sun) [USNM].

**Distribution.** China (Taiwan).

### 3.25. Pseudohermenias *Obraztsov, 1960*

*Pseudohermenias* Obraztsov, 1960: 471. Type-species: *Phalaena clausthaliana* Saxesen, 1840 [=*Tortrix hercyniana* Bechstein et Scharfenberg,1804].

**Remarks.** No species had been recorded in Taiwan prior to this study.

#### 3.25.1. *Pseudohermenias hercyniana* (Bechstein et Scharfenberg, 1804)

*Pyralis abietana* Fabricius, 1787: 237. TL: Sveciae [Sweden]. Lectotype: male, ZMUC. 

*Phalaena hercymiana* Bechstein et Scharfenberg, 1804: 755. TL: German. Syntype(s): unknown.

*Tortrix schmidtiana* Herrich−Schäffer, 1851: 221. TL: German. Syntype(s): unknown.

Pseudohermenias clausthaliana: Liu et Bai, 1977: 76.

*Pseudohermenias hercyniana* (Bechstein et Scharfenberg): Razowski, 1983: 104.

*Pseudohermenias abietana* (Fabricius): Kuznetzov, 2001: 247.

**Material examined.** Taiwan: 1 ♀, Taichung Co., Chingshan, 1100 m, 8-11-V-1989, J. Heppner and H. Wang (TORT11864 ♀, FL087); 1 ♂, Taichung Co. Chingshan, 1100 m, 27-III/1-IV-1990, J. Heppner and H. Wang (TORT12152 ♂, FL280) [FSCA].

**Host plants.** Pinaceae: *Abies alba* Linn., *Picea excels* Link., and *Pinus sylvestris* Linn.

**Distribution.** China (Heilongjiang, Taiwan), Europe, Japan, and Russia (Far East).

**Remarks.** This species is recorded for the first time in Taiwan.

### 3.26. Rhodacra *Diakonoff, 1973*

*Corethrarcha* Diakonoff, 1973: 287. Type-species: *Argyroploce rupifera* Meyrick, 1909.

*Rhodacra* Diakonoff, 1973: 286. Type-species: *Argyroploce pyrrhocrossa* Meyrick, 1912.

#### 3.26.1. *Rhodacra pyrrhocrossa* (Meyrick, 1912)

*Argyroploce pyrrhocrossa* Meyrick, 1912: 874. TL: India, Assam, Khasi Hills. Lectotype: male, BMNH. 

*Rhodacra pyrrhocrossa* (Meyrick): Diakonoff, 1973: 286.

**Material examined.** Taiwan: 1 ♂, 1 ♀, Tikusiko, 17-X-1935, S. Issiki (Sun-301 ♀, USNM145158, YH Sun); 1 ♂, Taihoku, 6-XI-1923, S. Issiki, no abdomen; 1 ♀, Taihoku, 17-XII-1934, S. Issiki [USNM].

**Distribution.** China (Taiwan), Australia, India, Japan, Russia, Sri Lanka, and Thailand.

### 3.27. Rudisociaria *Falkovitsh, 1962*

*Rudisociaria* Falkovitsh, 1962: 41. Type-species: *Grapholitha (Sericoris) expeditana* Sneffen, 1883.

#### 3.27.1. *Rudisociaria velutinum* (Walsingham, 1900)

*Exartema velutinum* Walsingham, 1900: 125. TL: China (Mupin). Syntypes: male and female, BMNH. 

*Rudisociaria velutina* (Walsingham): Diakonoff, 1973: 495.

*Olethreutes velutinus* (Walsingham): Kawabe, 1982: 110.

*Rudisociaria velutinum* (Walsingham): Kuznetzov, 2001: 258.

**Material examined.** Taiwan: 1 ♂, Baibara, 23-III-43, S. Issiki, no abdomen; 1 ♀, Hassenzan, 30-VIII-1929, S. Issiki (Sun-302 ♀); 1 ♂, Musya, 19-VII-1925, S. Issiki, no abdomen; 1 ♀, Rengwati, 29-III-27, S. Issiki [USNM]. Kaohsiung Co. Liukuei, Shanpin For., 750 m, 16-23-III-1990, J. Heppner and H. Wang; no photo, 47 ♂♂, 13 ♀♀, Kaohsiung Co. Liukuei, Shanpin For., 750 m, 16-23-III-1990, J. Heppner and H. Wang (TORT12070 ♂, FL270; Gen. Sl. Bae UFL-087 ♂; TORT12120 ♀, FL271); 3 ♂♂, Nantou Co. 5 km N. Chitou, 500 m, 29-VI-1985, J. Heppner and H. Wang (TORT12062 ♂); 1 ♂, Kaohsiung Co., Shanpin Forest Sta., Liukuei, 750 m, 5-6-VII-1996, J. Heppner and H. Wang (TORT12032 ♂); 1 ♂, Ilan Co. Tuchan, 480 m, 1-2-VII-1982, J. Heppner (no photo, Gen. Sl. Bae UFL-088 ♂); 2 ♂♂, Nantou Co. Lienhuachih For. Sta. Puli, 700 m, 4-VII-1996, J. Heppner and H. Wang (TORT12040 ♂, FL218); 1 ♂, Hualien Co. Fuyan, 400 m, 7-III-1990, J. Heppner and H. Wang (no photo, Gen. Sl. Bae UFL-096 ♂) [FSCA].

**Distribution.** China (Anhui, Gansu, Guangdong, Guangxi, Guizhou, Hunan, Shaanxi, Sichuan, Taiwan, Tianjin, Zhejiang), Korea, Japan, and Russia.

**Remarks.** This species is recorded for the first time in Taiwan.

### 3.28. Semniotes *Diakonoff, 1973*

*Semniotes* Diakonoff, 1973: 218. Type-species: *Argyroploce halantha* Meyrick, 1909.

#### 3.28.1. *Semniotes abrupta* Diakonoff, 1973

*Semniotes abrupta* Diakonoff, 1973: 220. TL: Borneo, East Borneo (Gunungsari). Holotype: male, RMNH.

**Material examined.** Taiwan: 14 ♂♂, Pingtung Co. Kenting Bot. Garden subtropical forest, 260 m, 10-14-VII-1980, D. R. Davis; 3 ♂♂, 2 ♀♀, Pintung Hsien, Kenting Park, 100 m, 26-27-III-1984, A. Kawabe (Sun-305 ♂, USNM145159, YH Sun; USNM122568 ♂, A. Kawabe; Sun-306 ♀, USNM145160, YH Sun; USNM122567 ♀, A. Kawabe); 1 ♀, Kuraru, 20-III-26, S. Issiki; 1 ♂, Kuraru, 6-IV-1943, A. Mutuura; 1 ♂, Sozan, 3-VI-1934, S. Issiki; 1 ♀, Sozan, 10-VI-1934, S. Issiki; 1 ♀, Sozan, 13-V-1935, S. Issiki; 1 ♀, Tiponosen, 28-III-44, S. Issiki; 1 ♂, Hassenzan, 30-VIII-1929, S. Issiki; 1 ♀, Kurai, 20-III-26, S. Issiki [USNM]. 

7 ♂♂, 6 ♀♀, Pingtung Co. Kenting Park, 255 m, 23-28-IV-1989, J. Heppner and H. Wang; 9 ♂♂, 7 ♀♀, Pingtung Co. Kenting Park, 255 m, 9-15-III-1990, J. Heppner and H. Wang (Gen. Sl. Bae UFL-021 ♂; Gen. Sl. Bae UFL-022 ♂); 3 ♂♂, 2 ♀♀, Pingtung Co. Kenting Park, 50 m, 1-5-IX-1983, J. Heppner; 1 ♀, Pingtung Co. Kenting Park, 50 m, 29-31-VIII-1983, J. Heppner [FSCA].

**Distribution.** China (Taiwan) and Indonesia.

### 3.29. Sorolopha *Lower, 1901*

*Sorolopha* Lower, 1901: 73. Type-species: *Sorolopha cyclotoma* Lower, 1901.

*Acanthothyspoda* Lower, 1908: 319. Type-species: *Acanthothyspoda elaeodes* Lower, 1908. 

*Alypeta* Turner, 1916: 528. Type-species: *Alypeta delochlora* Turner, 1916. 

*Alytopeta* Fletcher, 1929: 10 [misspelling of *Alypeta*]. 

*Choganhia* Razowski, 1960: 387. Type-species: *Argyroploce sphaerocopa* Meyrick, 1930. 

#### 3.29.1. *Sorolopha aeolochlora* (Meyrick, 1916)

*Argyroploce aeolochlora* Meyrick, 1916: 562. TL: India, Assam, Khasi Hills. Holotype: male, BMNH.

*Choganhia aeolochlora* (Meyrick): Razowski, 1960: 387.

*Eudemis (Acanthothyspoda) aeolochlora* (Meyrick): Diakonoff, 1968: 51.

*Sorolopha aeolochlora* (Meyrick): Diakonoff, 1973: 88.

**Material examined.** Taiwan: 2 ♂♂, 1 ♀, Chiai Hsien, 2270 m, 8-10-VIII-1985, A. Kawabe (Sun-307 ♂, USNM122570, A. Kawabe; Sun-308 ♀, USNM122569, A. Kawabe); 1 ♂, 2 ♀, Taitung Co. Yakou, 2750 m, 25-X-1984, J. Heppner and H. Wang (Gen. Sl. Bae UFL-002 ♂; TORT11840 ♀, FL038); 1 ♀, Taitung Co. Yakou, 2750 m, 25-X-1984, J. Heppner and H. Wang; 1 ♂, Ilan Co. Fushan For. Sta., 650 m, 4-11-IV-1990, J. Heppner (Gen. Sl. Bae UFL-053 ♂) [USNM].

**Distribution.** China (Taiwan) and India.

#### 3.29.2. *Sorolopha agana* (Falkovitsh, 1966)

*Choganhia agana* Falkovitsh, 1966: 209. TL: China (Chekiang Province, Hangchow). Holotype: male, MGAB. 

*Sorolopha agana* (Falkovitsh): Diakonoff, 1973: 95.

**Material examined.** Taiwan: 1 ♀, Nantou Hsien, Lushan spa, 1200 m, 3-5-VIII-1985, A. Kawabe; 1 ♂, Taoyuan Hsien, Palin, 1000 m, 31-VII-2-VIII-1985, A. Kawabe (Sun-309 ♂, USNM122571, A. Kawabe) [USNM]. 

1 ♀, Hualien Co. Fuyan, 400 m, 7-III-1990, J. Heppner and H. Wang (TORT11839 ♀, FL037) [FSCA].

**Distribution.** China (Hunan, Jiangxi, Sichuan, Taiwan, Zhejiang).

**Remarks.** This species is recorded for the first time in Taiwan.

#### 3.29.3. *Sorolopha angustorbis* Sun, Sun et Heppner, 2017

*Soroloph angustorbis* Sun, Sun et Heppner, 2017: 642. TL: Taiwan (Taitung Co. Bishan Hot Spgs). Holotype: male, MGCL.

**Material examined.** Taiwan: 1 ♂, Nantou Hsien, Lushan spa, 1200 m, July 27-29, 1983, A. Kawabe [USNM]. In addition, 2 ♂♂, Taitung Co. Bishan Hot Spgs., 720 m, 26-29-X-1984, J. Heppner and H. Wang (TORT12060 ♂, FL212); 1 ♂, 1 ♀, Kaohsiung Co. Lukuei For. Sta., 750 m, 29-IV/3-V-1989, J. Heppner and H. Wang (TORT12160 ♂, Gen. Sl. Bae UFL-054; Gen. Sl. Bae UFL-055 ♀); 1 ♂, Ilan Co. Fushan For. Sta., 650 m, 4-11-IV-1990, J. Heppner (Gen. Sl. Bae UFL-004 ♂) [FSCA].

**Distribution.** China (Taiwan) and Vietnam.

#### 3.29.4. *Sorolopha bryana* (Felder et Rogenhofer, 1875)

*Penthina bryana* Felder et Rogenhofer, 1875: pl. 137, Figure 54. TL: Sri Lanka [Sri Lanka]. Holotype: female, BMNH. 

*Sorolopha bryana* (Felder et Rogenhofer): Diakonoff, 1973: 89.

**Material examined.** Taiwan: 2 ♂♂, 3 ♀♀, Chiai Hsien, Fenchihu, 1400 m, 11-12-VIII-1985, A. Kawabe (Sun-311 ♀); 1 ♂, Chiai Hsien, Fenchihu, 1400 m, 11-12-VIII-1985, A. Kawabe (Sun-310 ♂, USNM122572, A. Kawabe); 1 ♂, 2 ♀♀, Chiai Hsien, Alishan, 2270 m, 8-10-VIII-1985, A. Kawabe (USNM123821 ♀, A. Kawabe); 1 ♂, Nantou Hsien, Lushan spa, 1200 m, 3-5-VIII-1985, A. Kawabe; 1 ♀, Kao Hsiung Co., 10-11 km NE Chiahsien, ca. 300 m, 3-8-VII-1980, D. R. Davis; 1 ♀, Nantow Co. Mei-feng, 30 km S Tayuling, 2200 m, 1-8-VI-1980, D. R. Davis [USNM]. 

1 ♂, Kaohsiung Co. Liukuei, Shanpin For., 750 m, 16-23-III-1990, J. Heppner and H. Wang (TORT12078 ♂, FL206); 1 ♀, Nantou Co., Meifeng, 2100 m, 6-9-IX-1988, J. Heppner (Gen. Sl. Bae UFL-006 ♀) [FSCA].

Fujian: 1 ♂, Pikeng, Mt. Wuyi, 600 m, 27-V-2004, Haili Yu [NKU].

**Distribution.** China (Fujian, Taiwan) and Sri Lanka. 

#### 3.29.5. *Sorolopha camarotis* (Meyrick, 1936)

*Argyroploce camarotis* Meyrick, 1936: 612. TL: India (Bengal, Kalimpong). Lectotype: male, BMNH. 

*Olethreutes camarotis* (Meyrick): Clarke, 1958: 491.

*Eudemis (Acanthothyspoda) camarotis* (Meyrick): Diakonoff, 1967: 52.

*Sorolopha camaroti* (Meyricks): Diakonoff, 1973: 96.

*Sorolopha longurus* Liu et Bai, 1982: 168. 

**Material examined.** Taiwan: 1 ♂, Kaohsiung Co. Liukuei, Shanpin For., 750 m, 16-23-III-1990, J. Heppner and H. Wang (TORT12068 ♂, Gen. Sl. Bae UFL-063) [FSCA].

**Host plants.** Magnoliaceae: *Michelia fuscata* Blume and *M. Sampaca* Linn.

**Distribution.** China (Fujian, Hainan, Jiangxi, Taiwan, Yunnan), India, Indonesia, Malaysia, and Thailand.

**Remarks.** This species is recorded for the first time in Taiwan.

#### 3.29.6. *Sorolopha elaeodes* (Lower, 1908)

*Acanthothyspoda elaeodes* Lower, 1908: 320. TL: Australia, Queensland, Cairns. Lectotype: male, SAMA [lost].

*Argyroploce lasiosoma* Meyrick, 1921: 449. TL: Australia, Queensland, Cairns. Holotype: male, SAMA.

*Argyroploce temenopis* Meyrick, 1936: 614. TL: Taiwan. Formosa [Taiwan] (Kagi). Lectotype: female, BMNH. 

*Sorolopha elaeodes* ssp. *parachlora* Diakonoff, 1973: 63. TL: Indonesia. South Celebes, low country. Holotype: female, BMNH. 

*Sorolopha elaeodes* (Lower): Kawabe, 1992: 106.

**Material examined.** Hainan: Mt. Yingge: 1 ♀, Hongkan (19.08° N, 109.50° E), 540 m, 27-VII-2015, Qingyun Wang, Suran Li, and Mengting Chen (BX15266) [NKU].

**Distribution.** China (Hainan, Taiwan) and Australia.

**Remarks.** The authors have not seen the specimens collected from Taiwan.

#### 3.29.7. *Sorolopha herbifera* (Meyrick, 1909)

*Argyroploce herbifera* Meyrick, 1909: 603. TL: India, Assam, Khasi Hills. Lectotype: male, BMNH. 

*Sorolopha herbifera* (Meyrick): Diakonoff, 1973: 72.

**Material examined.** Taiwan: 1 ♂, Nantou Hsien, Lushan spa, 1200 m, 3-5-VIII-1985, A. Kawabe; 1 ♂, Chiai Hsien, Fenchihu, 1400 m, 11-12-VIII-1985, A. Kawabe (Sun-313, USNM94012, N. Pinkaew) [USNM]. 

1 ♂, Taipei Co. Wulai, 200 m, 17-19-VI-1985, J. Heppner and H. Wang (TORT12080 ♂, FL184); 1 ♂, Ilan Co. 5 km N. Nanao, 100 m, 11-13-X-1984, J. Heppner and H. Wang; 1 ♀, Ilan Co. Chilan Nsy., Tuchan, 480 m, 15-17-X-1984, J. Heppner and H. Wang (TORT12081 ♀, FL185) [FSCA].

Hainan: Mt. Limu: 1 ♂, Forest Park (19.17° N, 109.73° E), 607 m, 15-V-2015, Peixin Cong, Wei Guan, and Sha Hu (BX16368) [NKU].

**Host plant.** Lauraceae: *Cinnamomum camphora* Linn.

**Distribution.** China (Hainan, Taiwan), India, Indonesia, Thailand, and Vietnam.

#### 3.29.8. *Sorolopha liochlora* (Meyrick, 1914)

*Argyroploce liochlora* Meyrick, 1914: 771. TL: India (Ganesh Gudi, Kanara). TD: BMNH.

*Olethreutes liochlora* (Meyrick): Clarke, 1958: 559.

*Sorolopha liochlora* (Meyrick): Diakonoff, 1973: 64.

**Material examined.** Hainan: Mt. Limu: 1 ♂, Forest Park (19.17° N, 109.73° E), 610 m, 5-V-2017, Xiaofei Yang. Wuzhishan City: 1 ♂, Nature Reserves (18.88° N, 109.65° E), 742 m, 20-V-2015, Peixin Cong, Wei Guan, and Sha Hu (BX15271) [NKU].

**Distribution.** China (Hainan, Taiwan) and India. 

**Remarks.** The authors have not seen the specimens collected from Taiwan.

#### 3.29.9. *Sorolopha muscida* (Wileman et Stringer, 1929)

*Argyroploce muscida* Wileman et Stringer, 1929: 67. TL: Taiwan, Formosa [Taiwan] (Rantaizan). Holotype: male, BMNH. 

*Sorolopha muscida* (Wileman et Stringer): Kawabe, 1992: 106.

**Material examined.** Taiwan: 3 ♂♂, 11 ♀♀, Nantou Hsien, Tsuifeng, 2400 m, 29-XII-1989, A. Kawabe; 5 ♂♂, 7 ♀♀, Chiai Hsien, Alishan, 2270 m, 8-10-VIII-1985, A. Kawabe; 1 ♂, 1 ♀, (Alishan) Chiai Hsien, Formosa, 12-VIII-1974, Y. Kishida; 1 ♂, Chiai Hsien, Fenchihu, 1400 m, 11-12-VIII-1985, A. Kawabe; 1 ♂, Nantow Co. Tayuling, 2500 m, 9-18-VI-1980, D. R. Davis; 4 ♂♂, 1 ♀, Nantou Hsien, Lushan spa, 1200 m, 7-8-VI-1988, K. Fujisawa; 1 ♀, Nantou Hsien, Tsingjing Farm, 1900 m, 9-VI-1988, K. Fujisawa; 1 ♀, Hualien Hsien, Houhuanshan, 3100 m, 10-11-VI-1988, K. Fujisawa; 1 ♀, Tattaka, 19-VII-1925, S. Issiki; 1 ♂, Rantaisan, 4-VI-1927, S. Issiki; 1 ♂, Taihoku, May 9, 1935, S. Issiki; 1 ♀, Taihoku, 6-IX-1934, S. Issiki; 1 ♀, Taihoku, 20-IX-1933, S. Issiki [USNM]. 

114 ♂♂, 52 ♀♀, Taitung Co. Yakou, 2750 m, 25-X-1984, J. Heppner and H. Wang (no photo, FL070 ♀); 6 ♂♂, 8 ♀♀, Nantou Co., Meifeng, 2100 m, 6-9-IX-1988, J. Heppner (TORT11841 ♂, FL067; TORT11842 ♂, FL068); 1 ♂, Ilan Co., Taipingshan, 1900 m, 13-V-1989, J. Heppner and H. Wang; 1 ♀, Kaohsiung Co. Liukuei, Shanpin For., 750 m, 16-23-III-1990, J. Heppner and H. Wang; 1 ♂, Ilan Co. Mingchyr Rec. Area, 1250 m, 9-10-VII-1996, J. Heppner and H. Wang; 1 ♂, Ilan Co. Fushan For. Sta., 650 m, 4-11-IV-1990, J. Heppner; 1 ♂, Hsinchu Co. Litonshan, 1450-1914 m, 13-VII-2005, J. Heppner; 1 ♂, 1 ♀, Nantou Co. Meifeng Hort. Sta., 2100 m, 6-10-IX-1988, J. Heppner and H. Wang; 3 ♀♀, Kaohsiung Co. Dona, 16 km NE. Maolin, 500 m, 3-XI-1984, J. Heppner and H. Wang; 2 ♂♂, 2 ♀♀, Hsinchu/Miaoli Kuangwu, 2000 m, 24-25-VI-1985, J. Heppner and H. Wang; 2 ♂♂, 1 ♀, Kaohsiung Co. Tienchih, 2210 m, 24-X-1984, J. Heppner and H. Wang; 1 ♀, Taipei Co. Wulai, 200 m, 17-19-VI-1985, J. Heppner and H. Wang; 1 ♂, Hsinchu Co. Kuangwu For. Sta., 2000 m, 18-25-VIII-1988, J. Heppner and H. Wang; 1 ♂, Taichung Co. Chingshan, 1100 m, 31-VIII/4-IX-1988, J. Heppner and H. Wang; 1 ♀, Chiayi Co. Fennchihwu, 1450 m, 2-4-VII-1985, J. Heppner and H. Wang; 1 ♀, Taitung Co. Bishan Hot Spgs., 720 m, 26-29-X-1984, J. Heppner and H. Wang; 1 ♀, Ilan Co. Tuchan, 480 m, 1-2-VII-1982, J. Heppner [FSCA].

**Distribution.** China (Taiwan).

#### 3.29.10. *Sorolopha phyllochlora* (Meyrick, 1905)

*Platypeplus phyllochlora* Meyrick, 1905: 585. TL: Ceylon [Sri Lanka] (Haragam). Lectotype: male, BMNH. 

*Eucosma phyllochlora*: Meyrick, 1906: 137.

*Argyroploce phyllochlora* (Meyrick): Meyrick, 1909: 592.

*Argyroploce ptilosoma* Meyrick, 1916: 563.

*Olethreutes phyllochlora* (Meyrick): Clarke, 1958: 535.

*Olethreutes ptilosoma* (Meyrick): Clarke, 1958: 535.

*Eudemis (Acanthothyspoda) phyllochlora* (Meyrick): Diakonoff, 1967: 52.

*Sorolopha phyllochlora* (Meyrick): Diakonoff, 1973: 68.

**Material examined.** Taiwan: 1 ♂, Chiai Hsien, Alishan, 2270 m, 8-10-VIII-1985, A. Kawabe; 1 ♂, Pingtung Co. Kenting Bot. Garden subtropical forest, 260 m, 10-14-VII-1980, D. R. Davis (Sun-312 ♂, USNM94011, N. Pinkaew); 1 ♀, Taihoku, 13-XII-1934, S. Issiki; 1 ♂, Kagi, 19-IX-1923, Fukuda; 1 ♀, Formosa, Koshun, 25-IV-25-V-1918, J. Sonan, K. Miyake, M. Yoshino; 1 ♂, 1 ♀, Pingtung Co. Kenting Bot. Garden subtropical forest, 260 m, 10-14-VII-1980, D. R. Davis; 1 ♀, Pingtung Co. Kenting Bot. Garden subtropical forest, 260 m, 22-25-VII-1980, D. R. Davis; 1 ♀, Hualien Hsien, Houhuanshan, 3100 m, 30-VII-1-VIII-1983, A. Kawabe; 1 ♀, Chiai Hsien, Alishan, 2270 m, 8-10-VIII-1985, A. Kawabe (Sun-317 ♀, USNM122576, A. Kawabe) [USNM]. 

3 ♂♂, 1 ♀, Taipei Co. Wulai, 200 m, 17-19-VI-1985, J. Heppner and H. Wang; 1 ♂, Taitung Co. Yakou, 2750 m, 25-X-1984, J. Heppner and H. Wang; 1 ♀, Hsinchu/Miaoli Kuangwu, 2000 m, 24-25-VI-1985, J. Heppner and H. Wang; 2 ♂♂, 1 ♀, Taichung Co. Chingshan, 1100 m, 31-VIII/4-IX-1988, J. Heppner and H. Wang (Gen. Sl. Bae UFL-001 ♂; TORT11843 ♂, FL069; TORT12012 ♀, FL162); 1 ♂, Hualien Co. Fuyan, 400 m, 7-III-1990, J. Heppner and H. Wang; 1 ♂, Ilan Co. Chilan Hostel, 480 m, 3-4-VIII-1992, J. Heppner; 1 ♀, Pingtung Co. Kenting Park, 50 m, 1-5-IX-1983, J. Heppner; 1 ♀, Pingtung Co. Kenting Park, 50 m, 29-31-VIII-1983, J. Heppner; 1 ♂, Ilan Co. 5 km N. Nanao, 100 m, 11-13-X-1984, J. Heppner and H. Wang (Gen. Sl. Bae UFL-068 ♂) [FSCA].

**Distribution.** China (Taiwan, Yunnan), India, and Sri Lanka.

**Remarks.** This species is recorded for the first time in Taiwan.

#### 3.29.11. *Sorolopha plinthograpta* (Meyrick, 1931)

*Argyroploce plinthograpta* Meyrick, 1931: 135. TL: Taiwan, Formosa [Taiwan] (Taihoku). Holotype: male, BMNH.

*Eudemis plinthograpta*: Issiki, 1957: 69.

*Olethreutes plinthograpta*: Clarke, 1958: 539.

*Sorolopha plinthograpta* (Meyrick): Diakonoff, 1973: 84.

**Material examined.** Taiwan: 1 ♂, Taihoku, 6-XI-1923, S. Issiki (Sun-314 ♂) [USNM].

Hainan: Mt. Limu: 1 ♂, Forest Park (19.17° N, 109.73° E), 607 m, 15-V-2015, Peixin Cong, Wei Guan, and Sha Hu. Baisha County: 1 ♂, Yaxing Village, Nankai Town (19.02° N, 109.40° E), 321 m, 21-VI-2015, Peixin Cong, Wei Guan, and Sha Hu. Wuzhishan City: 1 ♂, Shuiman Town (18.86° N, 109.67° E), 700 m, 18-IV-2013, Yinghui Sun, Wei Guan, and Tengteng Liu (BX15226). Yunnan: 1 ♂, Rare Botanical Garden, Ruili City, 1000 m, 7-VIII-2005, Yingdang Ren; 1 ♂, Bubang Village, Mengla Town, 650 m, 22-VIII-2005, Yingdang Ren [NKU].

**Host plants.** Lauraceae: *Machilus thunbergii* Sieb. et Zucc.; *Cinnamomum camphora* Sieb.; and *C. japonicum* Linn.

**Distribution.** China (Guangdong, Hainan, Jiangxi, Taiwan, Yunnan), Korea, Japan, Russia, and Thailand.

#### 3.29.12. *Sorolopha plumboviridis* Diakonoff, 1973

*Sorolopha plumboviridis* Diakonoff, 1973: 83. TL: Indonesia, Molucca Islands, Batian Island. Holotype: male, BMNH. 

**Material examined.** Taiwan: 1 ♀, Pingtung Co. Kenting Bot. Garden subtropical forest, 260 m, 10-14-VII-1980, D. R. Davis (Sun-315 ♀, USNM145161, YH Sun); 1 ♂, Lushan Nan Tow Hsien, Formosa, 6-VIII-1974, H. Nakajima [USNM].

Yunnan: 1 ♂, Rare Botanical Garden, Ruili City, 1000 m, 5-VIII-2005, Yingdang Ren [NKU].

**Distribution.** China (Yunnan, Taiwan), Indonesia, and Thailand.

#### 3.29.13. *Sorolopha rubescens* Diakonoff, 1973

*Sorolopha rubescens* Diakonoff, 1973: 70. TL: Indonesia, Molucca Islands, Halmahera Island, Gani. Holotype: male, BMNH. 

*Sorolopha chlorotica* Liu et Bai, 1985: 136. TL: China. Yunnan Province, Xiaomenglun. Holotype: male, IZAS.

**Material examined.** Taiwan: 1 ♂, Chiai Hsien, Alishan, 2270 m, 8-10-VIII-1985, A. Kawabe; 1 ♂, Nantou Hsien, Yushih, 1700 m, 28-XII-1988, A. Kawabe [USNM]. 

1 ♂, 1 ♀, Taipei Co. Wulai, 200 m, 17-19-VI-1985, J. Heppner and H. Wang (Gen. Sl. Bae UFL-077 ♂; TORT12038 ♀, FL205); 1 ♂, Taipei Co. Wulai, 200 m, 2-3-VII-2005, J. Heppner and H. Wang (TORT12017 ♂, FL204); 1 ♀, Kaohsiung Co. Liukuei, Shanpin For., 750 m, 16-23-III-1990, J. Heppner and H. Wang (TORT12039 ♀); 1 ♂, Ilan Co. 5 km N. Nanao, 100 m, 11-13-X-1984, J. Heppner and H. Wang [FSCA].

Hainan: Mt. Wuzhi: 2 ♂, Nature Reserves (18.90° N, 109.67° E), 738 m, 29-VII~29-X-2016, Xia Bai, Shuonan Qian, and Wanding Qi. Mt. Jianfeng: 1 ♂, Nature Reserves (18.74° N, 108.87° E), 770 m, 5-VI-2015, Peixin Cong, Wei Guan, and Sha Hu (BX15268). Yunnan: 1 ♂, Bubang Village, Mengla Town, 650 m, 23-VIII-2005, Yingdang Ren; 1 ♀, Guanping Town, Mengyang, 1200 m, 20-VIII-2005, Yingdang Ren [NKU].

**Distribution.** China (Hainan, Taiwan, Yunnan), Indonesia, and Thailand.

#### 3.29.14. *Sorolopha semiculta* (Meyrick, 1909)

*Argyroploce semiculta* Meyrick, 1909: 604. TL: Sri Lanka, Ceylon [Sri Lanka] (Hakgala). Lectotype: male, BMNH. 

*Argyroploce heteraspis* Meyrick, 1936: 614. TL: Taiwan. Formosa [Taiwan] (Kagi). Holotype: female, BMNH. 

*Olethreutes semiculta*: Clarke, 1958: 547.

*Sorolopha semiculta* (Meyrick): Diakonoff, 1973: 76.

**Material examined.** Taiwan: 1 ♂, Taiwan, T. Siraki [USNM]. 

1 ♂, 1 ♀, Ilan Co. Chilan Nsy., Tuchan, 480 m, 15-17-X-1984, J. Heppner and H. Wang; 1 ♀, Nantou Co. Lien-Hua Chih For. Sta. 12 km. S. Pali, 700 m, 7-12-IX-1983, J. Heppner; 9 ♂♂, 4 ♀♀, Taipei Co. Wulai, 200 m, 17-19-VI-1985, J. Heppner and H. Wang (Gen. Sl. Bae UFL-003 ♂; Gen. Sl. Bae UFL-056 ♂; TORT12010 ♂, FL160; TORT12011 ♀, FL161) [FSCA]. 

Hainan: Mt. Limu: 1 ♀, Forest Park (19.17° N, 109.73° E), 607 m, 25-X-2016, Xia Bai, Shuonan Qian, and Wanding Qi (BX15530). Wuzhishan City: 1 ♂, Nature Reserves (18.90° N, 109.67° E), 740 m, 13-IV-2009, Qing Jin and Bingbing Hu (BX16506). Yunnan: 1 ♂, Tropical Botanical Garden, 570 m, 13-VIII-2005, Yingdang Ren [NKU].

**Host plants.** Lauraceae: Alceodaphne semicarpifolia Nees and Persea americana Mill.

**Distribution.** China (Hainan, Taiwan, Yunnan), India, Indonesia, Japan, Soloman Is., Sri Lanka, Thailand, and Vietnam.

#### 3.29.15. *Sorolopha sphaerocopa* (Meyrick, 1930)

*Argyroploce sphaerocopa* Meyrick, 1930: 719. TL: Vietnam, Vietnam (Tonkin, Cho ganh). Holotype: male, MNHN. 

*Sorolopha sphaerocopa* (Meyrick): Diakonoff, 1973: 86.

**Material examined.** Taiwan: 1 ♀, Ilan Co. 5 km N. Nanao, 100 m, 11-13-X-1984, J. Heppner and H. Wang; (TORT11847 ♀, FL033); 1 ♂, Nantou Hsien, Lushan spa, 1200 m, 3-5-VIII-1985, A. Kawabe (Sun-316 ♂, USNM122575, A. Kawabe) [FSCA].

**Distribution.** China (Taiwan), India, Indonesia, Japan, Thailand, and Vietnam.

### 3.30. Statherotis *Meyrick, 1909*

*Statherotis* Meyrick, 1909: 591. Type-species: *Statherotis decorata* Meyrick, 1909.

*Statheromeris* Diakonoff, 1973: 181. Type-species: *Statheromeris atrifracta* Diakonoff, 1973.

#### 3.30.1. *Statherotis discana* (Felder et Rogenhofer, 1875)

*Tortrix discana* Felder et Rogenhofer, 1875: pl. 137, Figure 41. TL: Molucca Islands, Amboina. Holotype: male, BMNH.

*Statherotis discana* (Felder et Rogenhofer): Diakonoff, 1973: 243.

*Statherotis discana* form *saturata* Diakonoff, 1973: 246. TL: Indonesia. West Java, Gede−Pangrango Mountains, Tjibodas. Holotype: male, RMNH. 

*Statherotis discana* ssp. *cuneata* Diakonoff, 1973: 247. TL: Indonesia. East Java, Tengger Mountains, Tretes. Holotype: male, RMNH. 

**Material examined.** Taiwan: 5 ♂♂, 9 ♀♀, Formosa, Taipei, 20-V-1959, M. F. Than (Sun-303 ♂; Sun-304 ♀); 1 ♀, Taiwan, Heito, 31-VIII-1933, S. Issiki; 2 ♂♂, 10 ♀♀, Kao Hsiung Co., 10-11 km NE Chiahsien, 300 m, 3-8-VII-1980, D. R. Davis; 1 ♀, Nantow Co. Lienhuachi Exp. For. Sta. 15 km SW Puli, 750 m, 22-26-V-1980, D. R. Davis; 1 ♂, Nantow Co. Sunmoon Lake, 760 m, 20-25-VI-1980, D. R. Davis; 1 ♀, TaiNanCo. 2-3 km S Kwantzuling, ca. 350 m, 26-28-VI-1980, D. R. Davis; 1 ♀, Taihoku, 25-IV-1946, S. Issiki; 1 ♀, Taihoku, 26-IV-1946, S. Issiki; 1 ♂, Taihoku, 29-IV-1946, S. Issiki; 1 ♀, Taihoku, 8-V-1935, S. Issiki; 1 ♂, Suiryuto, 12-X-1935, S. Issiki; 1 ♂, 1 ♀, Sirin, 26-IX-1933, S. Issiki; 1 ♂, Taitung Hsien, Chihpen spa, 200 m, 28-III-1984, A. Kawabe (USNM122578 ♂, A. Kawabe) [USNM]. 

2 ♂♂, Pingtung Co. Kenting Park, 255 m, 23-28-IV-1989, J. Heppner and H. Wang (Gen. Sl. Bae UFL-033); 1 ♂, Kaohsiung Co. Liukuei, Shanpin For., 750 m, 16-23-III-1990, J. Heppner and H. Wang; 1 ♂, Taipei Co. Wulai, 200 m, 17-19-VI-1985, J. Heppner and H. Wang; 1 ♂, Nantou Co. Lienhuachih For. Sta. 12 km. S. Puli, 700 m, 7-12-IX-1983, J. Heppner; 1 ♂, Kaohsiung Co. Dona, 16 km NE. Maolin, 500 m, 3-XI-1984, J. Heppner and H. Wang; 1 ♂, Kaohsiung Co. Baolai, 395 m, 23-X-1984, J. Heppner and H. Wang (TORT11952 ♂, FL062); 1 ♀, Taitung Co. Kukuan, 700 m, 6-VII-1985, J. Heppner and H. Wang (TORT11913 ♀, FL061); 1 ♀, Ilan Co. 5 km N. Nanao, 100 m, 11-13-X-1984, J. Heppner and H. Wang [FSCA]. 

Guangdong: 3 ♂♂, 4 ♀♀, Mt. He, 9~18-XII-2002, Guilin Liu and Binglan Zhang; 1 ♂, Milv Village, Nanping Town, Shangsi County, 770 m, 3-IV-2002, Shulian Hao and Huaijun Xue. Hainan: Haikou City: 1 ♂, Jinniuling Park (20.00° N, 110.31° E), 23 m, 31-V-2018, Ping Liu, Xia Bai, and Shuai Yu. Lingao County: 1 ♂, 1 ♀, Gaoshanling (19.93° N, 109.64° E), 168 m, 1~2-VI-2018, Ping Liu, Xia Bai, and Shuai Yu. Qiongzhong County: 1 ♂, Yangjiang Farm (19.30° N, 109.75° E), 200 m, 12-V-2013, Yinghui Sun, Wei Guan, and Tengteng Liu. Mt. Limu: 1 ♂, Forest Park (19.17° N, 109.73° E), 607 m, 18-VII-2016, Xia Bai, Shuonan Qian, and Wanding Qi. Mt. Bawang: 1 ♂, Work Station (19.12° N, 109.09° E), 1000 m, 23-IV-2009, Bingbing Hu and Qing Jin. Dongfang City: 1 ♂, Datian Nature Reserves (19.11° N, 108.79° E), 100 m, 28-IV-2009, Qing Jin and Bingbing Hu. In addition, 6 ♂♂, 1 ♀, Datian Nature Reserves (19.11° N, 108.79° E), 56 m, 4~7-VI-2018, Ping Liu, Xia Bai, and Shuai Yu (BX16435, BX16436); 4 ♂♂, Lemei Village (19.08° N, 108.84° E), 124 m, 8-VI-2018, Ping Liu, Xia Bai, and Shuai Yu. Mt. Yingge: 1 ♂, Hongkan Protection Station (19.08° N, 109.50° E), 508 m, 15-VI-2015, Peixin Cong, Wei Guan, and Sha Hu; 1 ♂, 1 ♀, Nankai Station (19.07° N, 109.40° E), 210~270 m, 15-VIII~8-XI-2016, Xia Bai, Shuonan Qian, and Wanding Qi (BX15490); 1 ♂, Yinggezui (19.05° N, 109.70° E), 599 m, 27-VII-2017, Xia Bai, Ping Liu, and Shuai Yu; 1 ♀, Yinggezui (19.05° N, 109.57° E), 623 m, 7-I-2018, Mujie Qi and Shuai Yu. Baisha County: 1 ♂, Hongxin Village, Yuanmen Town (19.07° N, 109.52° E), 445 m, 19-III-2016, Qingyun Wang, Suran Li, and Shengnan Zhao; 15 ♂♂, 2 ♀♀, Yaxing Village, Nankai Town (19.02° N, 109.40° E), 321 m, 21-VI-2015, Peixin Cong, Wei Guan, and Sha Hu (BX15211, BX15212). Wuzhishan City: 3 ♂♂, Maoyang Town (18.94° N, 109.51° E), 225 m, 19~20-IV-2009, Qing Jin and Bingbing Hu; 2 ♂♂, Shuiman Town (18.88° N, 109.67° E), 630 m, 16~17-IV-2009, Qing Jin and Bingbing Hu; 1 ♀, Ecological Tea Garden (18.88° N, 109.67° E), Shuiman Town, 690 m, 5-XI-2016, Xia Bai, Shuonan Qian, and Wanding Qi; 1 ♀, Nature Reserves (18.90° N, 109.67° E), 740 m, 12-IV-2009, Qing Jin and Bingbing Hu (BX15413); 1 ♂, Nature Reserves (8.90° N, 109.67° E), 738 m, 4-XI-2016, Xia Bai, Shuonan Qian, and Wanding Qi (BX15489). Mt. Jianfeng: 1 ♂, Mingfenggu (18.74° N108.84° E), 954 m, 9-VIII-2017, Xia Bai, Ping Liu, and Shuai Yu; 1 ♂, Tropical Arboretum, Jianfengling Town (18.70° N, 108.79° E), 40 m, 29-IV-2014, Tengteng Liu, Wei Guan, and Xuemei Hu. Taiwan: 1 ♂, Yongxing Farm, Lanyu County, 20 m, 20-VIII-2006, 1 ♂, Mt. Chai, Gaoxiong City, 50-100 m, 27-VII-2006, Houhun Li and Xicui Du [NKU].

**Distribution.** China (Guangdong, Guangxi, Hainan, Hong Kong, Taiwan), India, Indonesia, Japan, Malaysia, Papua New Guinea, the Philippines, Solomon Is., Thailand, and Vietnam.

#### 3.30.2. *Statherotis leucaspis* (Meyrick, 1902)

*Eucosma leucaspis* Meyrick, 1902: 126. TL: the Maldives, Minikoi Island. Lectotype: male, UMCE. 

*Argyroploce leucaspis* (Meyrick): Meyrick, 1909: 592.

*Olethreutes leucaspis* (Meyrick): Clarke, 1958: 524.

*Statherotis leucaspis* (Meyrick): Diakonoff, 1973: 242.

**Distribution.** China (Taiwan).

#### 3.30.3. *Statherotis olenarcha* (Meyrick, 1931)

*Argyroploce olenarcha* Meyrick, 1931: 136. TL: Taiwan, Formosa [Taiwan] (Kyuhabon). Holotype: female, BMNH. 

Olethreutes olenarcha: Clarke, 1958: 532.

*Statherotis olenarcha* (Meyrick): Diakonoff, 1968: 55.

**Material examined.** Taiwan: 1 ♀, Ilan Co. Chilan Nsy., Tuchan, 480 m, 15-17-X-1984, J. Heppner and H. Wang (TORT11844 ♀, FL054); 1 ♀, Chiayi Co. Fennchihwu, 1450 m, 2-4-VII-1985, J. Heppner and H. Wang; 1 ♂, Nantou Co., Meifeng, 2100 m, 6-9-IX-1988, J. Heppner; 2 ♂♂, Kaohsiung Co. Liukuei, Shanpin For., 750 m, 16-23-III-1990, J. Heppner and H. Wang (no photo, forewing broken, FL106 ♂); 1 ♂, Taitung Co. Bishan Hot Spgs., 720 m, 26-29-X-1984, J. Heppner and H. Wang (Gen. Sl. Bae UFL-032 ♂); 1 ♂, Taichung Co. Chingshan, 1100 m, 31-VIII/4-IX-1988, J. Heppner and H. Wang (TORT12124 ♂, FL261) [FSCA].

**Distribution.** China (Taiwan) and the Philippines.

### 3.31. Statherotmantis *Diakonoff, 1973*

*Statherotmantis* Diakonoff, 1973: 288. Type-species: *Proschistis pictana* Kuznetzov, 1969.

*Statheromantis* Razowski, 1977: 284 [misspelling of *Statherotmantis*].

#### 3.31.1. *Statherotmantis pictana* (Kuznetzov, 1969)

*Proschistis pictana* Kuznetzov, 1969: 355. TL: Russia, Kuril Islands, Kunashir Island, near Sernovodsk. Holotype: male, ZMAS.

*Statherotmantis pictana* (Kuznetzov): Diakonoff, 1973: 290.

**Material examined.** Taiwan: 1 ♂, 1 ♀, Taoyuan Hsien, Palin, 1100 m, 31-VII-2-VIII-1985, A. Kawabe (Sun-318 ♂, USNM145162, YH Sun; Sun-319 ♀, USNM145163, YH Sun); 1 ♂, Nantou Hsien, Lushan spa, 1200 m, 9-10-VIII-1987, A. Kawabe; 1 ♀, Hualien Hsien, Wenshan spa, 580 m, 4-5-VIII-1983, A. Kawabe [USNM].

**Distribution.** China (Taiwan), Japan, Korea, and Russia.

#### 3.31.2. *Stathrotmantis shicotana* (Kuznetzov, 1969)

*Proschistis shicotana* Kuznetzov, 1969: 357. TL: Russia (Kuril Islands, Shikotan Island, foot of Mt. Shikotan). Holotype: male, ZMAS. 

*Statherotmantis shicotana*: Diakonoff, 1973: 289.

**Material examined.** Taiwan: 1 ♀, Taitung Co. Bishan Hot Spgs., 720 m, 26-29-X-1984, J. Heppner and H. Wang (Gen. Sl. Bae UFL-035 ♀) [FSCA].

**Distribution.** China (Hebei, Henan, Hubei, Hunan, Shandong, Taiwan, Tianjin), Europe, Japan, and Russia (Far East).

**Remarks.** This species is recorded for the first time in Taiwan.

### 3.32. Statherotoxys *Diakonoff, 1973*

*Statherotoxys* Diakonoff, 1973: 195. Type-species: *Statherotoxys hypochrysa* Diakonoff, 1973.

*Statherotyxys* Razowski, 1977: 284 [misspelling of *Statherotoxys*].

#### 3.32.1. *Statherotoxys hedraea* (Meyrick, 1905)

*Platypeplus hedraea* Meyrick, 1905: 584. TL: Sri Lanka, Ceylon [Sri Lanka] (Kandy). Lectotype: male, BMNH. 

*Olethreutes hedraea* (Meyrick): Clarke, 1958: 515.

*Statherotoxys hedraea* (Meyrick): Diakonoff, 1973: 199.

**Material examined.** Taiwan: 1 ♂, Urai, 30-I-1926, S. Issiki (Sun-320 ♂, USNM145164, YH Sun); 1 ♀, Taihoku, 27-XII-1932, S. Issiki (Sun-321 ♀, USNM145165, YH Sun); 1 ♀, Nantow Co. Lienhuachi Exp. For. Sta. 15 km SW Puli, 750 m, 22-26-V-1980, D. R. Davis [USNM]. 

1 ♂, Kaohsiung Co. Liukuei, Shanpin For., 750 m, 16-23-III-1990, J. Heppner and H. Wang; 1 ♂, Taoyuan Co. Upper Palin, 1500 m, 7-9-VII-1985, J. Heppner and H. Wang (Gen. Sl. Bae UFL-034 ♂) [FSCA].

Hainan: Wuzhishan City: 1 ♀, Mt. Wuzhi (18.91° N, 109.68° E), 733 m, 21-XII-2017, Mujie Qi and Shuai Yu; 1 ♂, Fanye Village (18.87° N, 109.67° E), Shuiman Town, 430 m, 22-2014.IV.22, Tengteng Liu, Wei Guan and Xuemei Hu; 1 ♀, Wuzhishan Nature Reserves (18.90° N, 109.67° E), 740 m, 14-IV-2009, Qing Jin and Bingbing Hu (BX16498). Mt. Jianfeng: 1 ♂, Mingfenggu (18.74° N, 108.84° E), 954 m, 10-VIII-2017, Xia Bai, Ping Liu and Shuai Yu; 1 ♂, Nature Reserves (18.74° N, 108.84° E), 1050 m, 27-IV-2014, Tengteng Liu, Wei Guan, and Xuemei Hu (BX15272). Sichuan: 1 ♂, Qingyinge, Mt. Emei, 16-VI-1979, unknown [NKU].

**Distribution.** China (Hainan, Sichuan, Taiwan), India, Japan, Russia, and Sri Lanka.

### 3.33. Sycacantha *Diakonoff, 1959*

*Sycacantha* Diakonoff, 1959: 181. Type-species: *Phaecasiophora auriflora* Diakonoff, 1959.

**Remarks.** No species had been recorded in Taiwan prior to this study.

#### 3.33.1. *Sycacantha inodes* (Meyrick, 1911)

*Argyroploce inodes* Meyrick, 1911: 269. TL: New Guinea (Woodlark Island). Holotype: female, BMNH. 

*Argyroploce albitibiana* Meyrick, 1928: 447.

*Argyroploce conchifera* Meyrick, 1931: 130.

*Olethreutes albitibiana* (Meyrick): Bradley, 1961: 125.

*Sycacantha inodes inodes* Diakonoff, 1966: 19.

*Sycacantha inodes* (Meyrick): Kuznetzov, 2001: 237.

**Material examined.** Taiwan: 4 ♂♂, Taipei Co. Wulai, 200 m, 17-19-VI-1985, J. Heppner and H. Wang (TORT12118 ♂, FL253); 1 ♀, Ilan Co. Chilan Nsy., Tuchan, 480 m, 15-17-X-1984, J. Heppner and H. Wang (no photo, FL254 ♀) [FSCA].

**Distribution.** China (Taiwan, Yunnan), Indonesia, New Guinea, and Solomon Islands.

**Remarks.** This species is recorded for the first time in Taiwan.

### 3.34. Temnolopha *Lower, 1901*

*Temnolopha* Lower, 1901: 72. Type-species: *Temnolopha mosaica* Lower, 1901.

#### 3.34.1. *Temnolopha matura* Diakonoff, 1973

*Temnolopha matura* Diakonoff, 1973: 322. TL: Indonesia, East Borneo, Tabang, Bengen River. Holotype: female, RMNH. 

**Material examined.** Taiwan: 1 ♂, 1 ♀, Pingtung Co. Kenting Bot. Garden subtropical forest, 260 m, 10-14-VII-1980, D. R. Davis (Sun-322 ♂; Sun-323 ♀) [USNM].

**Distribution.** China (Taiwan) and Myanmar.

### 3.35. Theorica *Diakonoff, 1966*

*Theorica* Diakonoff, 1966: 58. Type-species: *Argyroploce lamyra* Meyrick, 1911.

#### 3.35.1. *Theorica lamyra* Meyrick, 1911

*Argyroploce lamyra* Meyrick, 1911: 268. TL: New Guinea, New Guinea (Woodlark Island). Lectotype: male, BMNH. 

*Theorica lamyra* (Meyrick): Diakonoff, 1966: 59.

**Distribution.** China (Taiwan) and New Guinea.

## 4. Discussion

Taiwan, a treasure island embedded in the vast Pacific Ocean, has a rich diversity of insect species with its unique geographical location, rich natural ecology, and variable climatic conditions. It is not only a natural habitat for many insect species but also a cradle for many endemic species. From tropical rainforests to alpine meadows, from humid coastlines to dry desertification, diverse ecosystems provide diverse living environments and food sources for insects, making Taiwan a sacred place for insect researchers and enthusiasts. In Taiwan, butterflies are known for their colorful and diverse species, attracting global attention.

The study of Taiwan Lepidoptera began in the mid-nineteenth century, with the publication of “*Lepidoptera of Taiwan*” in 1992 and the “*Taiwan Lepidoptera catalog: Supplement 1. Corrections and additions*” published in 2012 [3,4], which recorded 239 species of Taiwan Lepidoptera. The first author of this paper went to the National Museum of Natural History and the Florida Museum of Natural History to examine the specimens of Taiwan tortricids in their collections and identified a total of 299 species, including several new species and newly recorded species.

Since Taiwan faces the Fujian Province of China across the Taiwan Strait to the west and has similar habitats to many provinces on the Chinese mainland, this study supplemented the geographical distribution of the new tortricid moths on the Chinese mainland and provided basic data for subsequent biodiversity, ecology, and biogeography research.

**Table 1:** The Institutional abbreviations.

BMNH	The Natural History Museum, London, England [=formerly British Museum (Natural History)].
DEIB	Deutsches Entomologisches Institut, Berlin, Germany.
IZAS	Institute of Zoology, Academia Sinica, Beijing, China.
LNK	Landessammlungen fur Naturkunde, Karlsruhe, Germany (Staatichen Museum fr Naturkunde, Karlsruhe).
MGAB	Muzeul de Istoria Naturala Grigore Antipa, Bucharest, Romania.
MGCL	McGuire Center for Lepidoptera, Florida Museum of Natural History, Gainsville, Florida, USA.
MNHN	Museum National d’Histoire Naturelle, Paris, France.
MNHU	Sammlung am Museum für Naturkunde (formally Museum fur Naturkunde der Humboldt Universitat), Berlin, Germany.
NHRS	Naturhistoriska Riksmuseet, Stockholm, Sweden.
NKU	Insect Collection, College of life Sciences, Nankai University, Tianjin, China.
OPU	Entomological Laboratory, Osaka Prefecture University (formerly University of Osaka Prefecture), Sakai, Japan.
RMNH	Regional Museum of Natural History, Bhopal, India.
SAMA	South Australian Museum, Adelaide, Australia.
TD	type depository.
TL	type locality.
TMB	Termeszettudomanyi Muzeum Allattara (Hungarian Natural History Museum), Budapest, Hungary.
UMCE	University Museum, Cambridge, England.
USNM	National Museum of Natural History, Washington, DC, USA.
ZMAS	Zoological Institute, Russian Academy of Sciences, St. Petersburg (Leningrad), Russia.
ZMUC	Zoological Museum, University of Copenhagen, Denmark.

## Data Availability

The raw data supporting the conclusions of this article will be made available by the authors on request.

## References

[1] Obraztsov N.S. (1946). Versuch einer systematischen Übersicht der europäischen Eucosmini-Gattungen (Lepidoptera, Tortricidae). Z. Wien. Entomol. Ges..

[2] Gilligan T.M., Baixeras J., Brown J.W. T@RTS: Online World Catalogue of the Tortricidae. Version 4.0. http://www.tortricidae.com/catalogue.asp.

[3] Kawabe A., Komai F., Razowski J. (1992). Tortricoidea. Lepidoptera of Taiwan.

[4] Heppner J.B. (2012). Taiwan Lepidoptera catalog: Supplement 1. Corrections and additions. Lepid. Novae.

[5] Wu H., Pan C.W. (2001). Insects of Tianmushan National Nature Reserve.

[6] Huang B.K. (2001). Fauna of Insects in Fujian Province of China.

[7] Li H.H., Wang S.X. (2009). The Fauna of Hebei, China, Microlepidoptera.

[8] Li H.H. (2012). Microlepidoptera of Qinling Mountains (Insecta: Lepidoptera).

